# Supplementary material for: The multiscale self-similarity of the weighted human brain connectome
Source: PLoS Comput Biol. 2025 Apr 7;21(4):e1012848. doi: 10.1371/journal.pcbi.1012848 (PMC11991287; doi:10.1371/journal.pcbi.1012848)
Supplement: S1 File — (PDF) [file pcbi.1012848.s001.pdf]

The multiscale self-similarity of the weighted human brain connectome

Supporting Information UL Dataset

Laia Barjuan,[1,2] Muhua Zheng,[3] and M. Ángeles Serrano[1,2,4,*]

[1] *Departament de Física de la Matèria Condensada, Universitat de Barcelona, Martí i Franquès 1, Barcelona, Spain*
[2] *Universitat de Barcelona Institute of Complex Systems (UBICS), Universitat de Barcelona, Barcelona, Spain*
[3] *School of Physics and Electronic Engineering, Jiangsu University, Zhenjiang, Jiangsu, China*
[4] *ICREA, Passeig Lluís Companys 23, Barcelona, Spain*

CONTENTS

I. Flow of averages 6

II. Self-similarity 8

III. Weak ties hypothesis 13

IV. Other community detection algorithms 18

V. Fit of the model 19

VI. GR Model 20
  A. Flow of averages 20
  B. Self-similarity 21
  C. Weak ties in GR model 25

VII. Null models 28

* marian.serrano@ub.edu

Here we present all results obtained for the 40 connectomes in the UL Dataset. Regarding its multiscale structure, the following notation with respect to connectome layers is employed: layer 0 corresponds to the connectome with 1015 nodes, layer 1 to 463 nodes, layer 2 to 234 nodes, layer 3 to 129 nodes and layer 4 to 82 nodes.

Subject	layer	nodes	edges	$\langle k \rangle \pm$ SEM	$\langle w \rangle \pm$ SEM	$\langle s \rangle \pm$ SEM	η
No. 0	0	1009	14470	28.7 ± 0.7	0.0081 ± 0.0002	0.231 ± 0.006	0.89
	1	461	7177	31.1 ± 0.8	0.0072 ± 0.0002	0.225 ± 0.008	0.84
	2	233	3586	30.8 ± 0.9	0.0071 ± 0.0003	0.218 ± 0.010	0.72
	3	128	1744	27.2 ± 1.0	0.0076 ± 0.0004	0.208 ± 0.012	0.75
	4	82	962	23.5 ± 1.1	0.0079 ± 0.0005	0.186 ± 0.012	0.66
No. 1	0	1010	14406	28.5 ± 0.7	0.0069 ± 0.0001	0.196 ± 0.006	0.76
	1	461	7195	31.2 ± 0.9	0.0061 ± 0.0002	0.191 ± 0.007	0.73
	2	233	3893	33.4 ± 1.2	0.0056 ± 0.0002	0.187 ± 0.009	0.68
	3	128	1992	31.1 ± 1.3	0.0059 ± 0.0003	0.182 ± 0.012	0.88
	4	82	1129	27.5 ± 1.3	0.0060 ± 0.0004	0.166 ± 0.013	0.77
No. 2	0	1014	13671	27.0 ± 0.6	0.0075 ± 0.0002	0.202 ± 0.006	0.87
	1	462	6833	29.6 ± 0.8	0.0066 ± 0.0002	0.195 ± 0.007	0.91
	2	233	3599	30.9 ± 1.0	0.0061 ± 0.0002	0.189 ± 0.010	0.93
	3	128	1806	28.2 ± 1.0	0.0066 ± 0.0004	0.185 ± 0.013	0.79
	4	82	978	23.9 ± 1.1	0.0069 ± 0.0005	0.165 ± 0.014	1.17
No. 3	0	1011	12991	25.7 ± 0.6	0.0079 ± 0.0002	0.203 ± 0.006	0.81
	1	462	6642	28.8 ± 0.8	0.0069 ± 0.0002	0.198 ± 0.008	0.75
	2	233	3581	30.7 ± 1.0	0.0064 ± 0.0003	0.198 ± 0.010	0.72
	3	128	1888	29.5 ± 1.1	0.0066 ± 0.0004	0.195 ± 0.013	0.69
	4	82	1074	26.2 ± 1.3	0.0064 ± 0.0005	0.169 ± 0.014	0.91
No. 4	0	1014	15879	31.3 ± 0.8	0.0068 ± 0.0001	0.214 ± 0.006	0.70
	1	462	7896	34.2 ± 1.0	0.0061 ± 0.0002	0.207 ± 0.007	0.90
	2	233	4069	34.9 ± 1.2	0.0059 ± 0.0002	0.206 ± 0.009	0.74
	3	128	2077	32.5 ± 1.3	0.0062 ± 0.0003	0.200 ± 0.012	0.89
	4	82	1177	28.7 ± 1.4	0.0062 ± 0.0004	0.177 ± 0.012	0.98
No. 5	0	1014	14340	28.3 ± 0.7	0.0070 ± 0.0001	0.198 ± 0.005	0.80
	1	462	7175	31.1 ± 0.9	0.0062 ± 0.0002	0.191 ± 0.007	0.75
	2	233	3719	31.9 ± 1.0	0.0059 ± 0.0002	0.187 ± 0.009	0.86
	3	128	1919	30.0 ± 1.1	0.0061 ± 0.0003	0.183 ± 0.012	0.86
	4	82	1137	27.7 ± 1.3	0.0058 ± 0.0004	0.162 ± 0.012	0.82
No. 6	0	1013	12660	25.0 ± 0.7	0.0077 ± 0.0002	0.193 ± 0.006	0.97
	1	462	6519	28.2 ± 0.9	0.0067 ± 0.0002	0.190 ± 0.007	0.90
	2	233	3375	29.0 ± 1.0	0.0065 ± 0.0003	0.189 ± 0.010	0.87
	3	128	1712	26.8 ± 1.1	0.0069 ± 0.0004	0.185 ± 0.012	1.06
	4	82	1000	24.4 ± 1.2	0.0068 ± 0.0005	0.166 ± 0.012	1.14
No. 7	0	1014	13474	26.6 ± 0.7	0.0068 ± 0.0002	0.180 ± 0.006	0.95
	1	462	6955	30.1 ± 0.9	0.0059 ± 0.0002	0.177 ± 0.008	0.92
	2	233	3651	31.3 ± 1.0	0.0057 ± 0.0002	0.179 ± 0.010	1.12
	3	128	1934	30.2 ± 1.1	0.0059 ± 0.0003	0.177 ± 0.013	1.27
	4	82	1076	26.2 ± 1.2	0.0061 ± 0.0004	0.160 ± 0.013	1.25
No. 8	0	1002	13910	27.8 ± 0.7	0.0070 ± 0.0001	0.194 ± 0.005	0.88
	1	462	7041	30.5 ± 0.8	0.0062 ± 0.0002	0.190 ± 0.007	0.96
	2	233	3723	32.0 ± 1.1	0.0060 ± 0.0002	0.193 ± 0.010	0.95
	3	128	1960	30.6 ± 1.2	0.0063 ± 0.0003	0.192 ± 0.014	1.04
	4	82	1124	27.4 ± 1.3	0.0063 ± 0.0004	0.172 ± 0.014	1.17
No. 9	0	1010	13496	26.7 ± 0.6	0.0069 ± 0.0002	0.184 ± 0.005	0.87
	1	462	6658	28.8 ± 0.8	0.0061 ± 0.0002	0.176 ± 0.007	0.86
	2	233	3532	30.3 ± 0.9	0.0058 ± 0.0002	0.176 ± 0.009	0.84
	3	128	1774	27.7 ± 1.0	0.0063 ± 0.0003	0.175 ± 0.011	0.86
	4	82	977	23.8 ± 1.1	0.0068 ± 0.0005	0.162 ± 0.012	0.97
No. 10	0	1014	15222	30.0 ± 0.7	0.0071 ± 0.0001	0.214 ± 0.006	0.85
	1	462	7414	32.1 ± 0.9	0.0065 ± 0.0002	0.209 ± 0.008	0.77
	2	233	3754	32.2 ± 0.9	0.0065 ± 0.0003	0.210 ± 0.010	0.65
	3	128	1954	30.5 ± 1.0	0.0066 ± 0.0004	0.202 ± 0.013	0.69
	4	82	1102	26.9 ± 1.2	0.0067 ± 0.0005	0.180 ± 0.014	0.78

Subject	layer	nodes	edges	$\langle k \rangle \pm$ SEM	$\langle w \rangle \pm$ SEM	$\langle s \rangle \pm$ SEM	η
No. 11	0	1013	14695	29.0 ± 0.7	0.0074 ± 0.0002	0.215 ± 0.006	0.84
	1	462	7577	32.8 ± 0.9	0.0064 ± 0.0002	0.209 ± 0.008	0.77
	2	233	3872	33.2 ± 1.0	0.0063 ± 0.0003	0.210 ± 0.011	0.57
	3	128	2023	31.6 ± 1.1	0.0067 ± 0.0004	0.211 ± 0.014	0.78
	4	82	1088	26.5 ± 1.1	0.0076 ± 0.0006	0.200 ± 0.016	0.82
No. 12	0	1001	13933	27.8 ± 0.7	0.0081 ± 0.0002	0.226 ± 0.006	0.60
	1	460	7112	30.9 ± 0.9	0.0069 ± 0.0002	0.214 ± 0.007	0.79
	2	233	3655	31.4 ± 1.0	0.0066 ± 0.0003	0.206 ± 0.010	0.70
	3	128	1875	29.3 ± 1.2	0.0066 ± 0.0004	0.194 ± 0.013	0.74
	4	82	1049	25.6 ± 1.4	0.0064 ± 0.0005	0.164 ± 0.013	0.84
No. 13	0	1013	14409	28.4 ± 0.8	0.0073 ± 0.0002	0.207 ± 0.006	1.01
	1	462	7409	32.1 ± 1.0	0.0063 ± 0.0002	0.203 ± 0.008	0.78
	2	233	3845	33.0 ± 1.1	0.0061 ± 0.0002	0.201 ± 0.010	0.79
	3	128	2009	31.4 ± 1.3	0.0062 ± 0.0003	0.196 ± 0.012	0.74
	4	82	1163	28.4 ± 1.4	0.0064 ± 0.0005	0.180 ± 0.013	0.70
No. 14	0	1013	14959	29.5 ± 0.7	0.0069 ± 0.0001	0.203 ± 0.006	0.86
	1	462	7382	32.0 ± 0.9	0.0061 ± 0.0002	0.196 ± 0.007	0.72
	2	233	3808	32.7 ± 1.0	0.0059 ± 0.0002	0.192 ± 0.009	0.89
	3	128	1952	30.5 ± 1.1	0.0059 ± 0.0003	0.181 ± 0.011	1.09
	4	82	1071	26.1 ± 1.3	0.0059 ± 0.0004	0.155 ± 0.012	1.46
No. 15	0	1007	16208	32.2 ± 0.7	0.0056 ± 0.0001	0.179 ± 0.005	0.68
	1	462	8003	34.6 ± 1.0	0.0050 ± 0.0001	0.175 ± 0.007	0.74
	2	233	4102	35.2 ± 1.1	0.0048 ± 0.0002	0.170 ± 0.009	0.75
	3	128	2092	32.7 ± 1.2	0.0051 ± 0.0003	0.165 ± 0.011	0.78
	4	82	1150	28.0 ± 1.4	0.0052 ± 0.0004	0.145 ± 0.011	0.90
No. 16	0	1014	14539	28.7 ± 0.7	0.0065 ± 0.0001	0.187 ± 0.005	0.83
	1	462	7352	31.8 ± 1.0	0.0058 ± 0.0002	0.184 ± 0.007	0.82
	2	233	3801	32.6 ± 1.1	0.0057 ± 0.0002	0.186 ± 0.010	0.79
	3	128	1999	31.2 ± 1.3	0.0058 ± 0.0004	0.181 ± 0.012	0.84
	4	82	1198	29.2 ± 1.4	0.0055 ± 0.0004	0.161 ± 0.012	0.87
No. 17	0	1014	13901	27.4 ± 0.7	0.0074 ± 0.0002	0.202 ± 0.005	0.77
	1	462	7030	30.4 ± 0.9	0.0063 ± 0.0002	0.191 ± 0.006	0.70
	2	233	3642	31.3 ± 1.0	0.0060 ± 0.0002	0.187 ± 0.008	0.78
	3	128	1821	28.5 ± 1.1	0.0063 ± 0.0003	0.179 ± 0.010	0.81
	4	82	994	24.2 ± 1.2	0.0067 ± 0.0005	0.163 ± 0.011	0.75
No. 18	0	1014	12418	24.5 ± 0.7	0.0081 ± 0.0002	0.198 ± 0.006	1.04
	1	462	6510	28.2 ± 0.8	0.0068 ± 0.0002	0.192 ± 0.008	1.04
	2	233	3497	30.0 ± 1.0	0.0063 ± 0.0002	0.190 ± 0.010	0.92
	3	128	1847	28.9 ± 1.1	0.0065 ± 0.0003	0.188 ± 0.012	0.97
	4	82	1008	24.6 ± 1.1	0.0069 ± 0.0005	0.170 ± 0.013	1.02
No. 19	0	1011	14883	29.4 ± 0.7	0.0065 ± 0.0001	0.190 ± 0.005	0.91
	1	462	7633	33.0 ± 0.9	0.0056 ± 0.0002	0.185 ± 0.007	0.77
	2	233	4042	34.7 ± 1.1	0.0052 ± 0.0002	0.182 ± 0.009	0.88
	3	128	2032	31.8 ± 1.2	0.0056 ± 0.0003	0.177 ± 0.011	0.61
	4	82	1117	27.2 ± 1.4	0.0056 ± 0.0004	0.152 ± 0.011	0.78
No. 20	0	1011	13201	26.1 ± 0.7	0.0078 ± 0.0002	0.204 ± 0.006	0.86
	1	462	6749	29.2 ± 0.9	0.0066 ± 0.0002	0.194 ± 0.007	0.86
	2	233	3589	30.8 ± 1.0	0.0062 ± 0.0002	0.190 ± 0.008	0.77
	3	128	1816	28.4 ± 1.0	0.0063 ± 0.0003	0.180 ± 0.010	0.72
	4	82	975	23.8 ± 1.2	0.0067 ± 0.0005	0.159 ± 0.011	0.53
No. 21	0	1012	12004	23.7 ± 0.6	0.0093 ± 0.0002	0.220 ± 0.007	0.92
	1	462	6241	27.0 ± 0.8	0.0081 ± 0.0002	0.220 ± 0.009	0.95
	2	233	3200	27.5 ± 0.9	0.0080 ± 0.0003	0.219 ± 0.012	0.80
	3	128	1576	24.6 ± 1.0	0.0089 ± 0.0005	0.220 ± 0.016	0.84
	4	82	859	21.0 ± 1.1	0.0093 ± 0.0007	0.194 ± 0.016	0.99
No. 22	0	1014	13519	26.7 ± 0.7	0.0067 ± 0.0001	0.178 ± 0.005	0.76
	1	462	6669	28.9 ± 0.8	0.0061 ± 0.0002	0.176 ± 0.007	0.76
	2	233	3429	29.4 ± 0.9	0.0059 ± 0.0002	0.174 ± 0.009	0.82
	3	128	1758	27.5 ± 1.0	0.0063 ± 0.0003	0.173 ± 0.012	0.83
	4	82	989	24.1 ± 1.1	0.0064 ± 0.0004	0.155 ± 0.012	0.97

Subject	layer	nodes	edges	$\langle k \rangle \pm$ SEM	$\langle w \rangle \pm$ SEM	$\langle s \rangle \pm$ SEM	η
No. 23	0	1014	14709	29.0 ± 0.7	0.0078 ± 0.0002	0.227 ± 0.006	0.88
	1	462	7147	30.9 ± 0.9	0.0071 ± 0.0002	0.218 ± 0.008	0.71
	2	233	3783	32.5 ± 1.1	0.0066 ± 0.0002	0.213 ± 0.010	0.73
	3	128	1864	29.1 ± 1.1	0.0070 ± 0.0004	0.205 ± 0.013	0.93
	4	82	1031	25.1 ± 1.3	0.0072 ± 0.0005	0.182 ± 0.014	0.84
No. 24	0	1014	13661	26.9 ± 0.7	0.0069 ± 0.0001	0.187 ± 0.005	0.86
	1	462	6868	29.7 ± 0.8	0.0061 ± 0.0002	0.182 ± 0.007	1.02
	2	233	3587	30.8 ± 1.0	0.0059 ± 0.0002	0.183 ± 0.009	0.92
	3	128	1858	29.0 ± 1.1	0.0063 ± 0.0003	0.182 ± 0.011	0.74
	4	82	1033	25.2 ± 1.2	0.0064 ± 0.0004	0.162 ± 0.012	0.73
No. 25	0	1013	14802	29.2 ± 0.7	0.0080 ± 0.0002	0.233 ± 0.006	0.73
	1	462	7428	32.2 ± 1.0	0.0068 ± 0.0002	0.218 ± 0.008	0.67
	2	233	3893	33.4 ± 1.2	0.0063 ± 0.0002	0.211 ± 0.009	0.70
	3	128	2039	31.9 ± 1.3	0.0063 ± 0.0003	0.199 ± 0.011	0.66
	4	82	1200	29.3 ± 1.4	0.0059 ± 0.0004	0.171 ± 0.012	0.84
No. 26	0	1013	12942	25.6 ± 0.7	0.0083 ± 0.0002	0.213 ± 0.007	0.88
	1	462	6709	29.0 ± 0.9	0.0070 ± 0.0002	0.203 ± 0.008	0.70
	2	233	3445	29.6 ± 1.0	0.0067 ± 0.0003	0.197 ± 0.011	0.77
	3	128	1732	27.1 ± 1.1	0.0072 ± 0.0004	0.194 ± 0.013	0.82
	4	82	975	23.8 ± 1.2	0.0075 ± 0.0005	0.177 ± 0.015	0.83
No. 27	0	1014	12483	24.6 ± 0.6	0.0079 ± 0.0002	0.195 ± 0.006	0.83
	1	462	6352	27.5 ± 0.8	0.0069 ± 0.0002	0.190 ± 0.007	0.87
	2	233	3308	28.4 ± 0.9	0.0066 ± 0.0003	0.189 ± 0.009	0.74
	3	128	1766	27.6 ± 1.1	0.0068 ± 0.0004	0.187 ± 0.012	1.05
	4	82	981	23.9 ± 1.2	0.0069 ± 0.0005	0.165 ± 0.012	1.13
No. 28	0	1012	14812	29.3 ± 0.8	0.0066 ± 0.0001	0.194 ± 0.005	0.71
	1	462	7440	32.2 ± 1.0	0.0058 ± 0.0002	0.187 ± 0.007	0.64
	2	233	3995	34.3 ± 1.2	0.0053 ± 0.0002	0.181 ± 0.009	0.64
	3	128	2039	31.9 ± 1.3	0.0055 ± 0.0003	0.175 ± 0.011	0.74
	4	82	1151	28.1 ± 1.4	0.0058 ± 0.0004	0.162 ± 0.012	0.70
No. 29	0	1012	13601	26.9 ± 0.7	0.0068 ± 0.0001	0.183 ± 0.005	0.95
	1	462	6998	30.3 ± 0.9	0.0059 ± 0.0002	0.180 ± 0.007	0.77
	2	233	3694	31.7 ± 1.0	0.0056 ± 0.0002	0.179 ± 0.009	0.64
	3	128	1813	28.3 ± 1.0	0.0061 ± 0.0003	0.173 ± 0.011	0.69
	4	82	966	23.6 ± 1.1	0.0066 ± 0.0005	0.157 ± 0.011	0.86
No. 30	0	1013	14177	28.0 ± 0.7	0.0077 ± 0.0002	0.216 ± 0.006	0.79
	1	462	7246	31.4 ± 0.9	0.0067 ± 0.0002	0.211 ± 0.008	0.76
	2	233	3778	32.4 ± 1.1	0.0064 ± 0.0002	0.209 ± 0.010	0.80
	3	128	1960	30.6 ± 1.1	0.0066 ± 0.0003	0.203 ± 0.013	0.79
	4	82	1131	27.6 ± 1.3	0.0066 ± 0.0005	0.183 ± 0.013	0.97
No. 31	0	1014	15641	30.9 ± 0.8	0.0069 ± 0.0001	0.214 ± 0.005	0.71
	1	462	7983	34.6 ± 1.0	0.0059 ± 0.0002	0.205 ± 0.007	0.64
	2	233	4220	36.2 ± 1.1	0.0056 ± 0.0002	0.201 ± 0.009	0.64
	3	128	2141	33.5 ± 1.2	0.0058 ± 0.0003	0.195 ± 0.011	0.30
	4	82	1231	30.0 ± 1.4	0.0057 ± 0.0004	0.172 ± 0.011	0.24
No. 32	0	1013	12875	25.4 ± 0.7	0.0075 ± 0.0002	0.192 ± 0.005	0.74
	1	462	6409	27.7 ± 0.9	0.0068 ± 0.0002	0.189 ± 0.007	0.92
	2	233	3351	28.8 ± 1.1	0.0065 ± 0.0003	0.187 ± 0.009	0.72
	3	128	1795	28.0 ± 1.2	0.0067 ± 0.0004	0.188 ± 0.012	0.96
	4	82	1031	25.1 ± 1.3	0.0068 ± 0.0005	0.171 ± 0.013	1.09
No. 33	0	1014	14510	28.6 ± 0.7	0.0078 ± 0.0002	0.222 ± 0.006	0.71
	1	462	7427	32.2 ± 0.9	0.0066 ± 0.0002	0.212 ± 0.007	0.68
	2	233	3876	33.3 ± 1.1	0.0060 ± 0.0002	0.201 ± 0.009	0.70
	3	128	2004	31.3 ± 1.1	0.0061 ± 0.0003	0.191 ± 0.011	0.88
	4	82	1106	27.0 ± 1.3	0.0062 ± 0.0005	0.168 ± 0.012	0.81
No. 34	0	1012	14344	28.3 ± 0.7	0.0068 ± 0.0001	0.193 ± 0.005	0.77
	1	461	7292	31.6 ± 0.9	0.0060 ± 0.0002	0.188 ± 0.007	0.79
	2	233	3814	32.7 ± 1.0	0.0057 ± 0.0002	0.186 ± 0.009	0.70
	3	128	1921	30.0 ± 1.0	0.0062 ± 0.0003	0.185 ± 0.011	1.11
	4	82	1053	25.7 ± 1.2	0.0067 ± 0.0005	0.171 ± 0.012	1.14

Subject	layer	nodes	edges	$\langle k \rangle \pm$ SEM	$\langle w \rangle \pm$ SEM	$\langle s \rangle \pm$ SEM	η
	0	1009	12460	24.7 ± 0.7	0.0074 ± 0.0002	0.183 ± 0.006	0.88
	1	462	6467	28.0 ± 0.9	0.0065 ± 0.0002	0.181 ± 0.007	0.82
No. 35	2	233	3439	29.5 ± 1.0	0.0061 ± 0.0003	0.179 ± 0.010	0.79
	3	128	1808	28.2 ± 1.1	0.0062 ± 0.0004	0.174 ± 0.012	1.07
	4	82	1064	26.0 ± 1.3	0.0059 ± 0.0004	0.154 ± 0.013	1.03
	0	1014	13978	27.6 ± 0.7	0.0067 ± 0.0001	0.185 ± 0.006	0.87
	1	462	7053	30.5 ± 0.9	0.0059 ± 0.0002	0.181 ± 0.007	0.89
No. 36	2	233	3775	32.4 ± 1.1	0.0056 ± 0.0002	0.181 ± 0.010	1.06
	3	128	1915	29.9 ± 1.2	0.0059 ± 0.0003	0.176 ± 0.012	1.06
	4	82	1083	26.4 ± 1.3	0.0061 ± 0.0004	0.160 ± 0.012	1.38
	0	1014	14282	28.2 ± 0.7	0.0082 ± 0.0002	0.232 ± 0.007	0.87
	1	462	7216	31.2 ± 0.9	0.0073 ± 0.0002	0.227 ± 0.008	0.74
No. 37	2	233	3783	32.5 ± 1.0	0.0069 ± 0.0003	0.225 ± 0.011	0.74
	3	128	1940	30.3 ± 1.1	0.0073 ± 0.0004	0.220 ± 0.014	0.83
	4	82	1105	27.0 ± 1.3	0.0075 ± 0.0006	0.203 ± 0.015	0.81
	0	1011	14001	27.7 ± 0.7	0.0077 ± 0.0002	0.212 ± 0.005	0.76
	1	462	7129	30.9 ± 0.9	0.0066 ± 0.0002	0.203 ± 0.007	0.71
No. 38	2	233	3788	32.5 ± 1.1	0.0061 ± 0.0002	0.199 ± 0.009	0.71
	3	128	1952	30.5 ± 1.2	0.0062 ± 0.0003	0.188 ± 0.011	0.70
	4	82	1115	27.2 ± 1.4	0.0059 ± 0.0004	0.160 ± 0.011	0.70
	0	1013	12599	24.9 ± 0.7	0.0069 ± 0.0002	0.173 ± 0.006	0.82
	1	462	6468	28.0 ± 0.9	0.0061 ± 0.0002	0.172 ± 0.008	0.80
No. 39	2	233	3433	29.5 ± 1.1	0.0058 ± 0.0003	0.171 ± 0.010	0.98
	3	128	1808	28.2 ± 1.2	0.0059 ± 0.0004	0.168 ± 0.013	1.23
	4	82	1026	25.0 ± 1.3	0.0061 ± 0.0005	0.152 ± 0.013	1.11
	0	1014	14418	28.4 ± 0.7	0.0095 ± 0.0002	0.271 ± 0.006	0.84
	1	462	7013	30.4 ± 0.9	0.0077 ± 0.0002	0.233 ± 0.008	0.84
Frankie	2	233	3773	32.4 ± 1.0	0.0064 ± 0.0002	0.208 ± 0.010	0.92
	3	128	1860	29.1 ± 1.1	0.0068 ± 0.0004	0.197 ± 0.012	0.97
	4	82	1056	25.8 ± 1.3	0.0067 ± 0.0005	0.172 ± 0.012	1.01

A: Overview of the 40 connectomes in the UL dataset. Number of nodes, number of edges, average degree, average weight, average strength and corresponding ±1 standard error interval around the mean (SEM), and exponent of the strength-degree power-law relationship, $s \propto k^{\eta}$.

I. FLOW OF AVERAGES

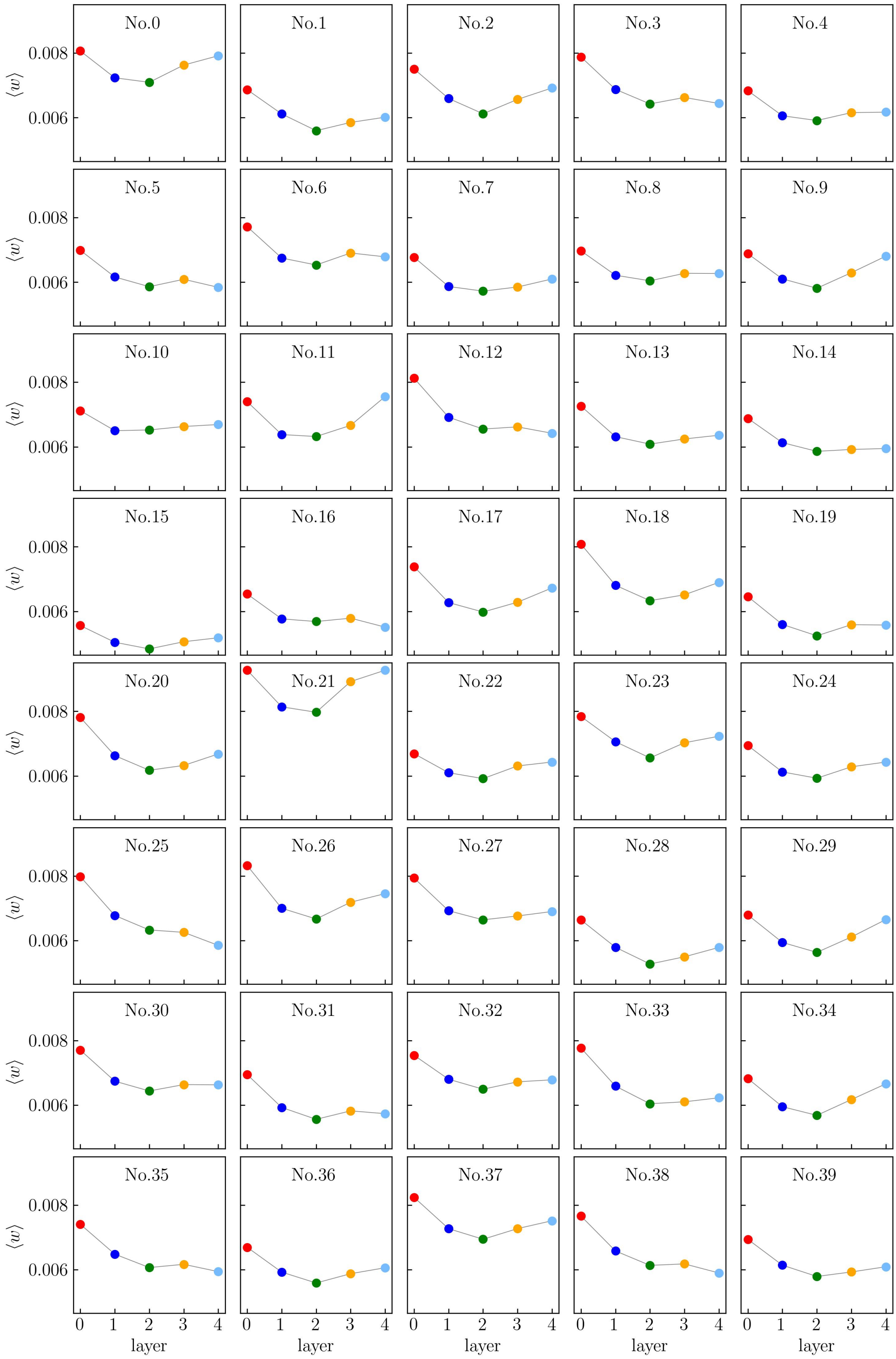

A: Evolution of average weight in consecutive layers.

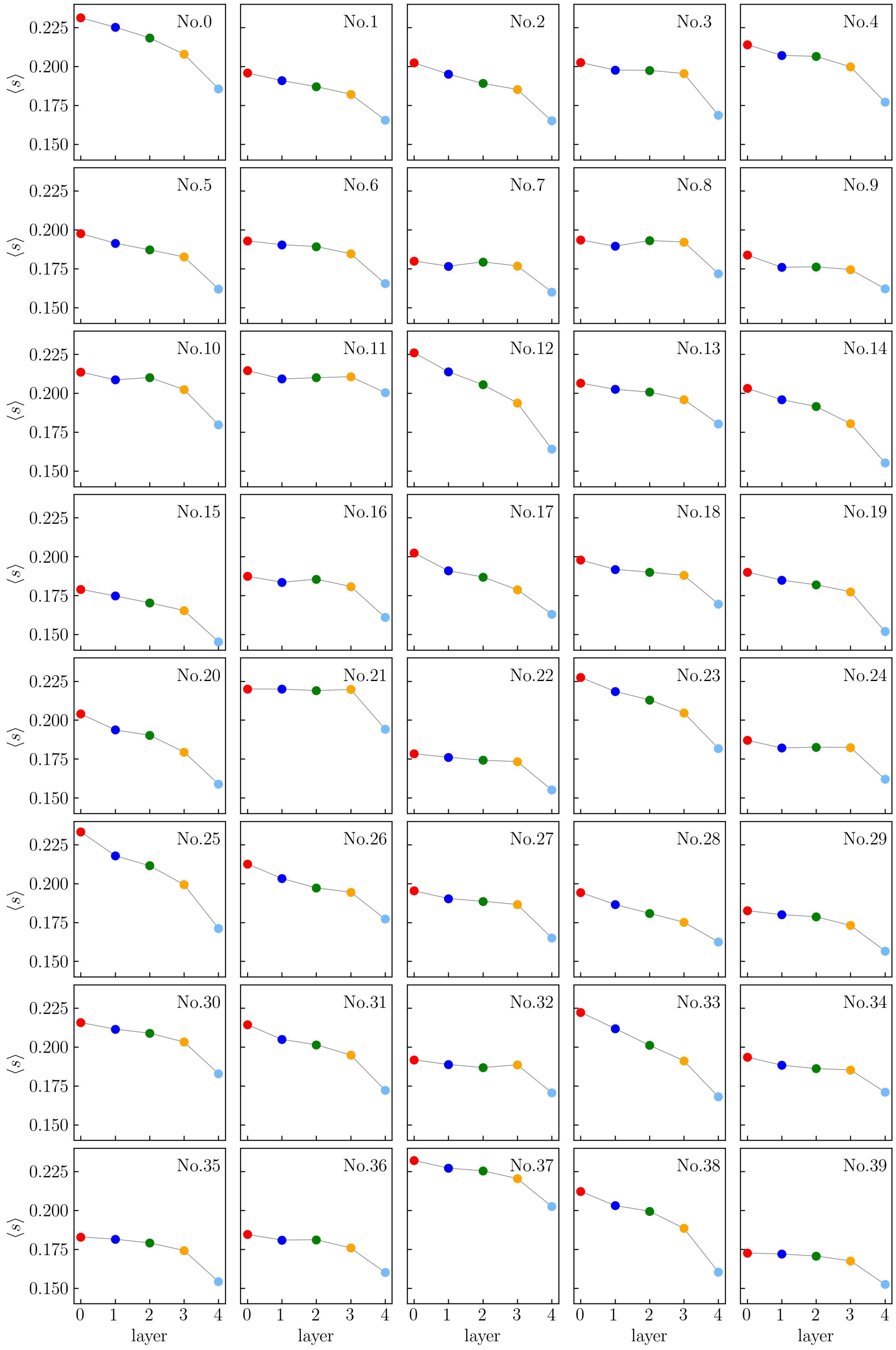

B: Evolution of average strength in consecutive layers.

II. SELF-SIMILARITY

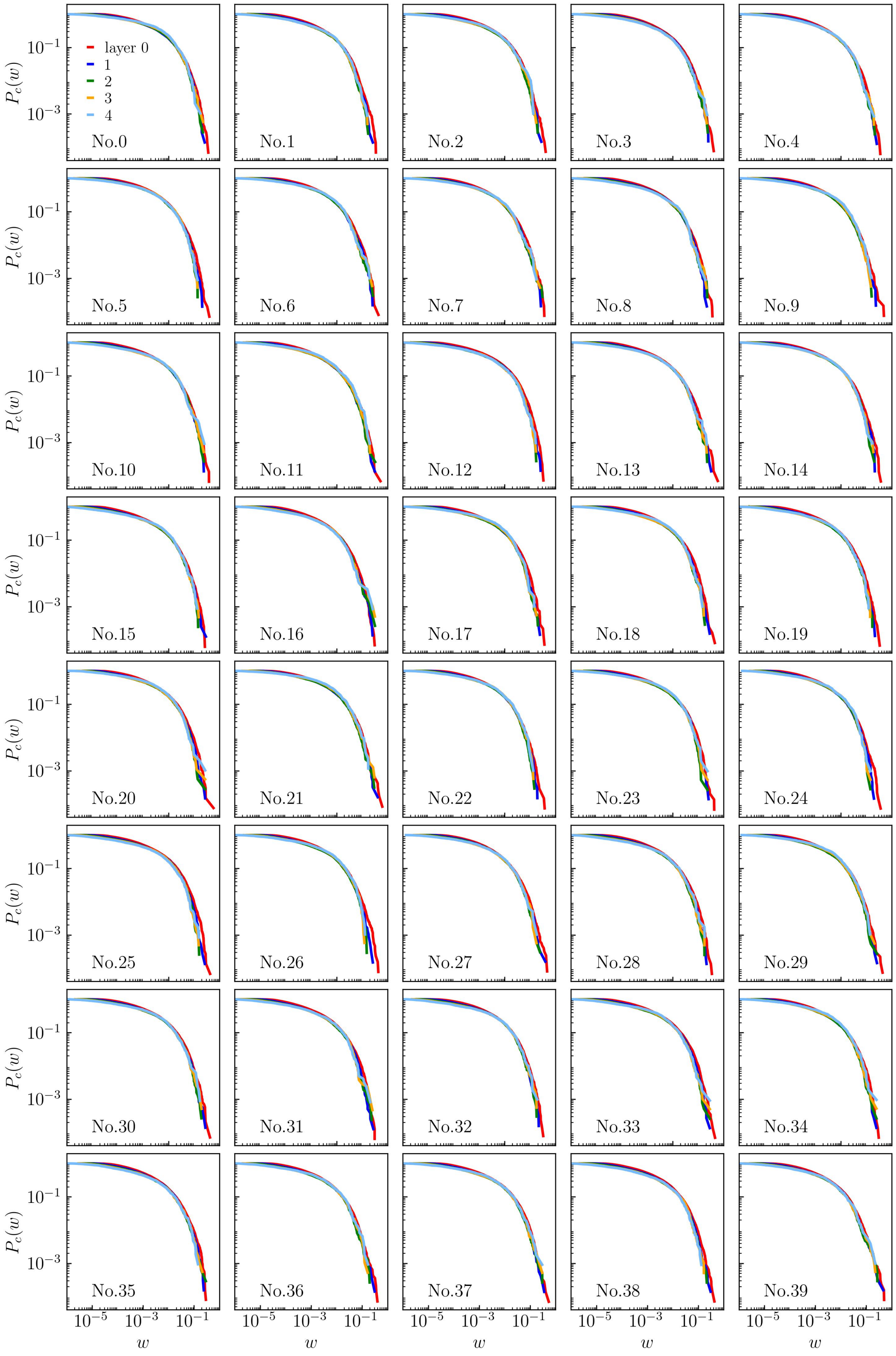

C: Complementary cumulative weight distribution in consecutive layers..

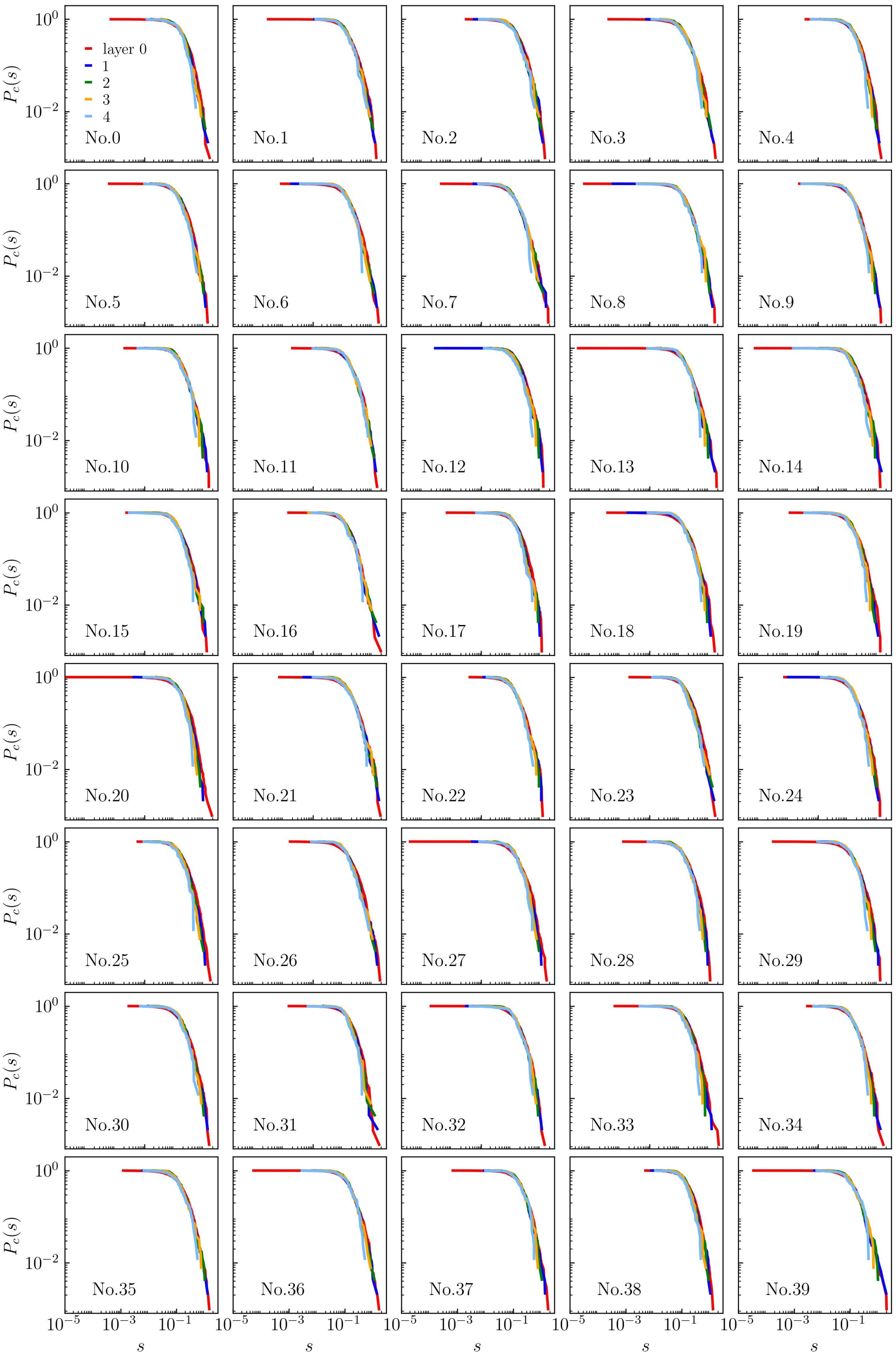

D: Complementary cumulative strength distribution in consecutive layers.

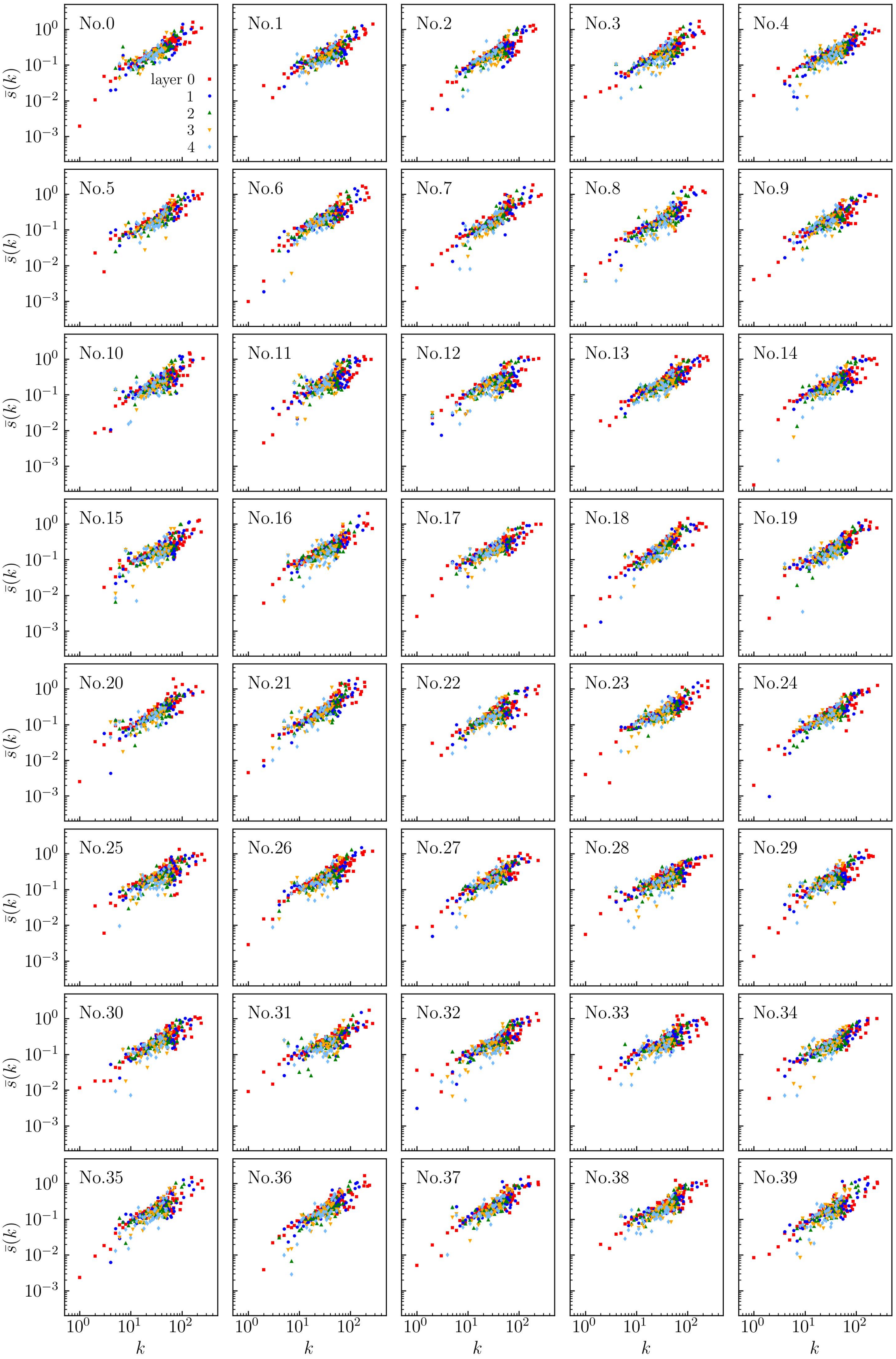

E: Strength-degree correlation in consecutive layers.

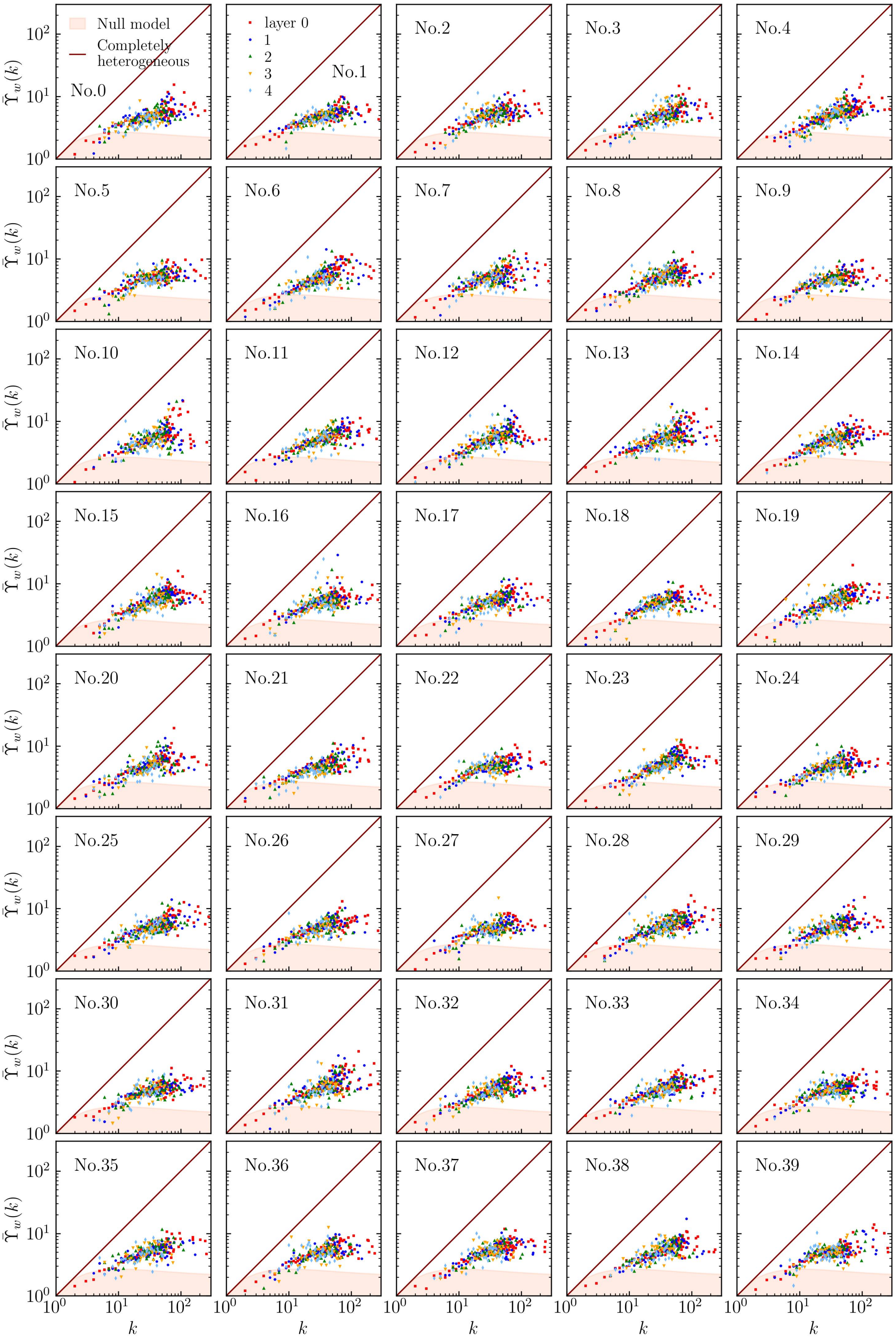

F: Disparity measure in consecutive layers.

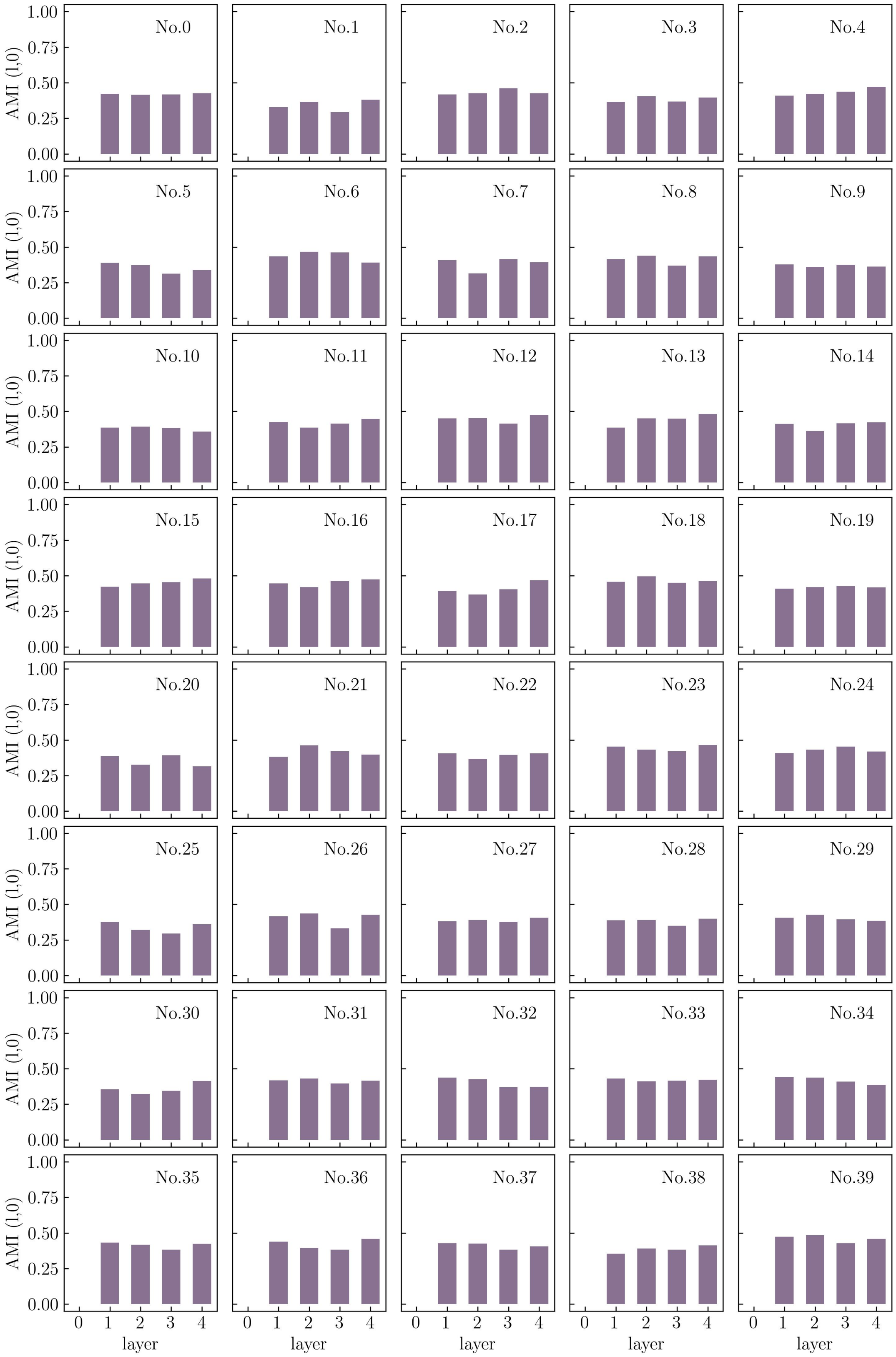

G: Adjusted Mutual Information between communities in layer l (x-axis) and communities in layer 0.

III. WEAK TIES HYPOTHESIS

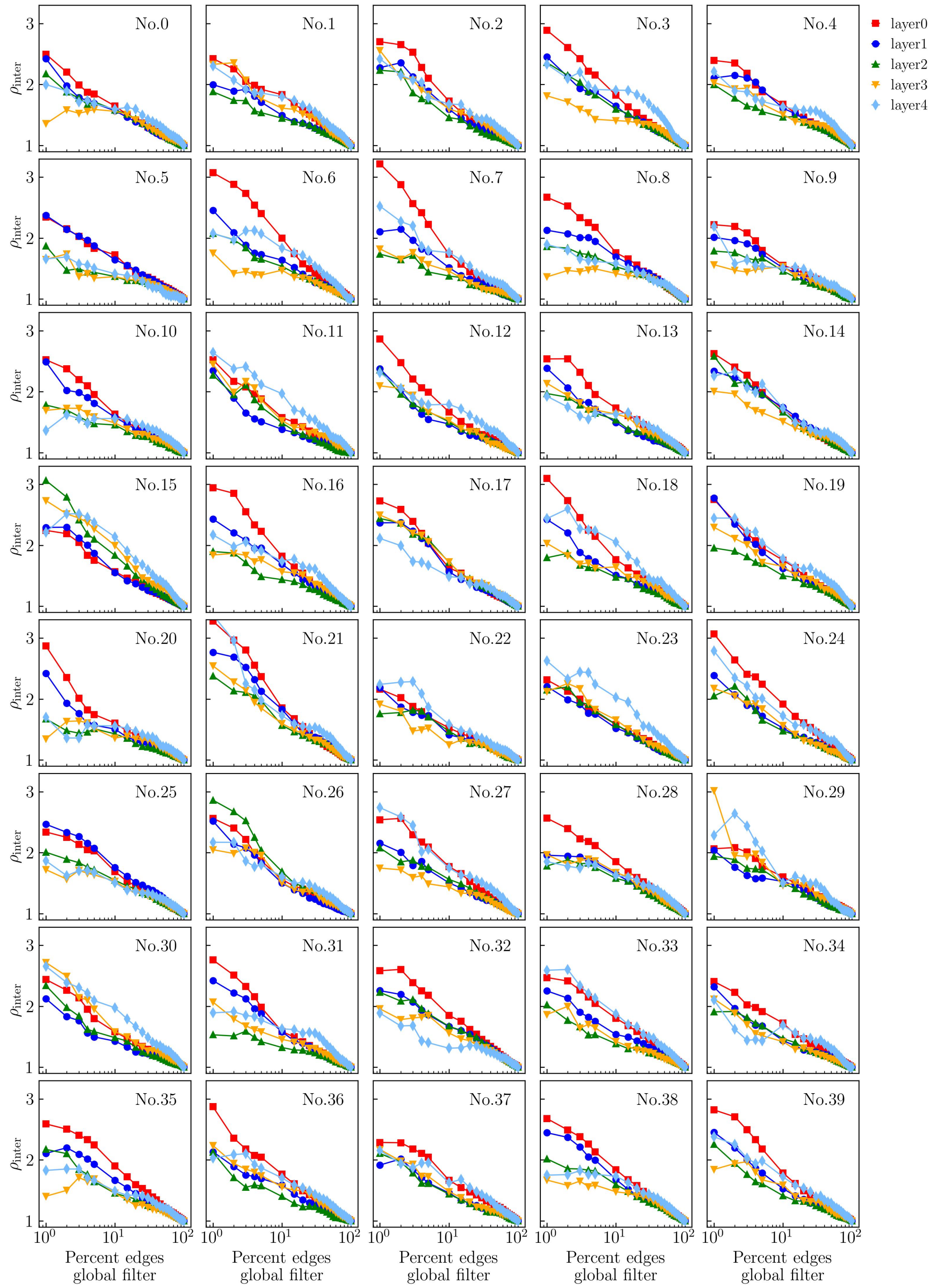

H: Normalized density of intermodular connections versus the percent of edges considered. The x axis X% means we are taking into account the X% of connections with lowest weight. X-axis in log scale.

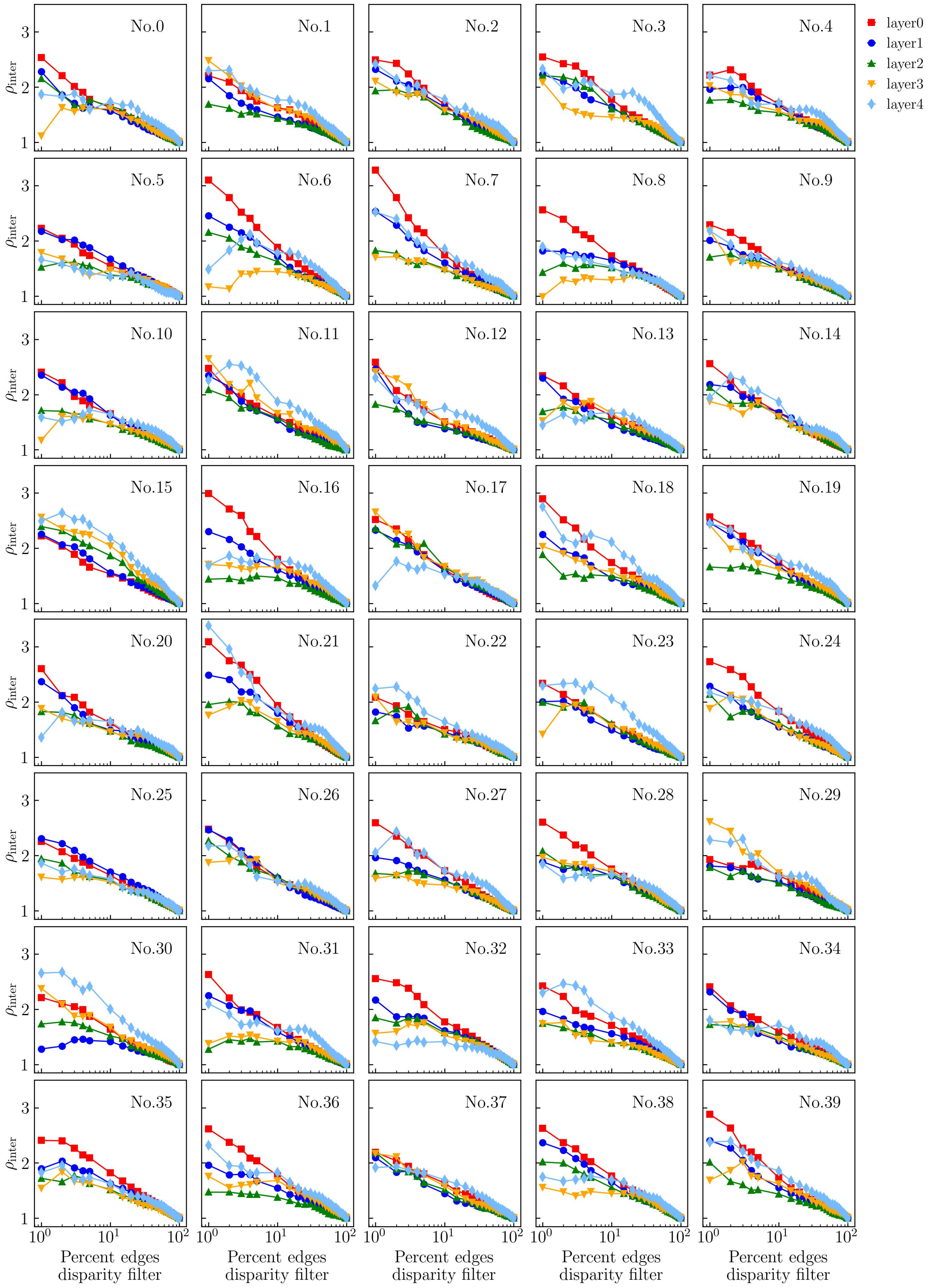

I: Normalized density of intermodular connections versus the percent of edges considered. The x axis X% means we are taking into account the X% of connections with lowest $1-\alpha$. X-axis in logscale.

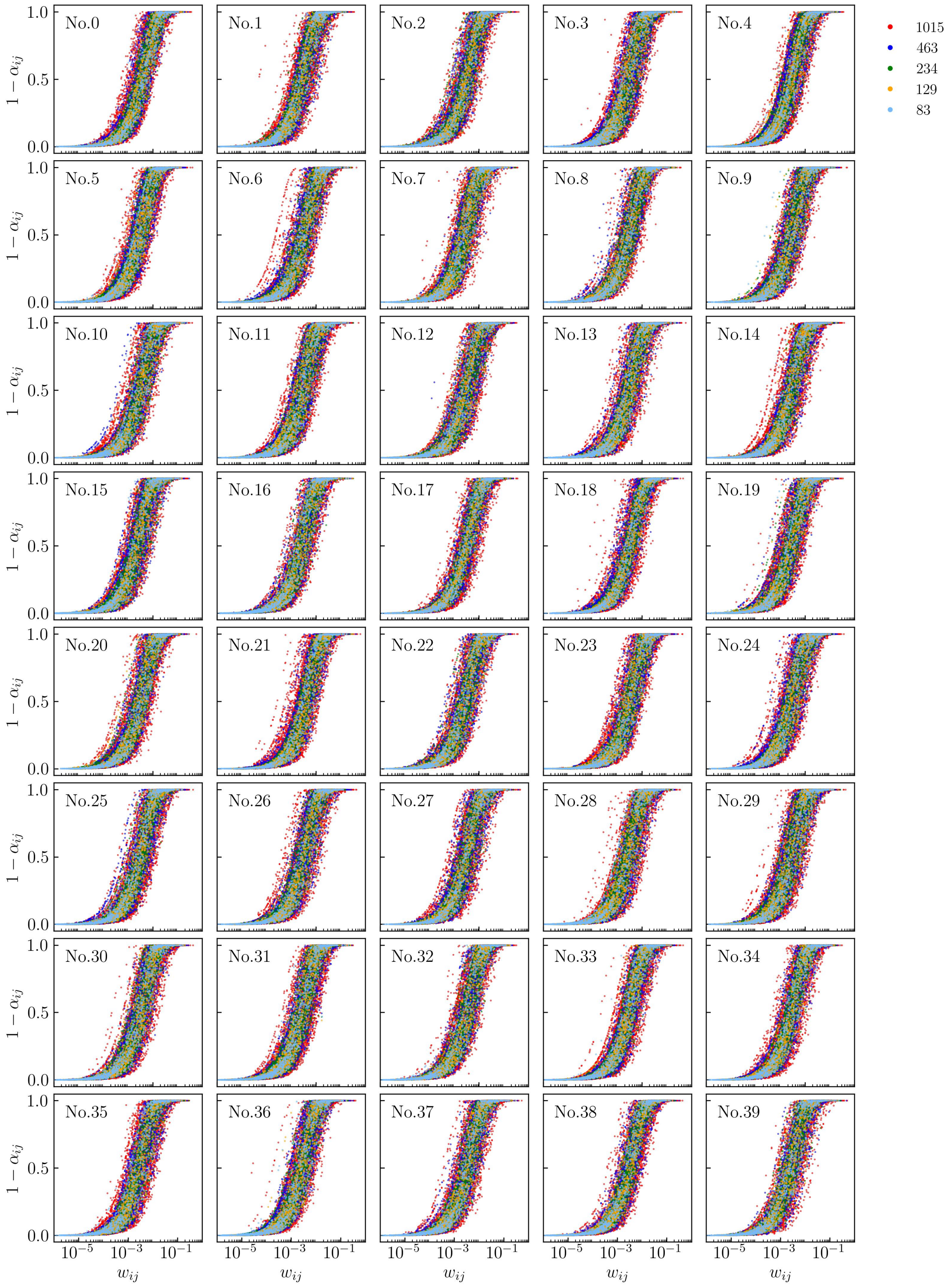

J: Relationship between weight and the confidence of the edge, $1-\alpha$. Legend corresponds to the number of nodes in that layer.

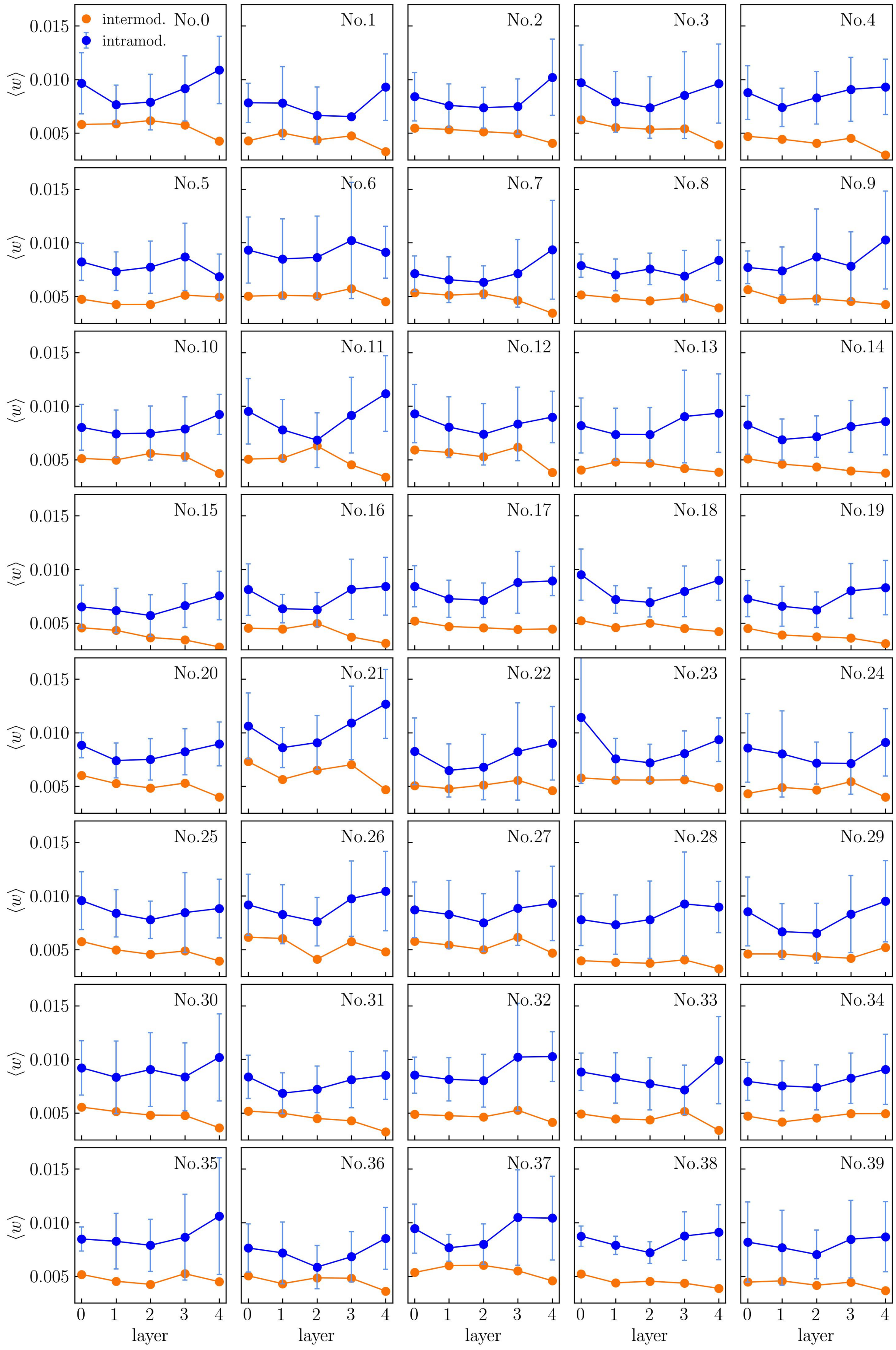

K: Average weight inside modules (intramodular links) compared to average weight outside modules (intermodular links), when modules are computed using the Louvain algorithm. The average weight inside modules is computed as the mean of the average weights in the different modules.

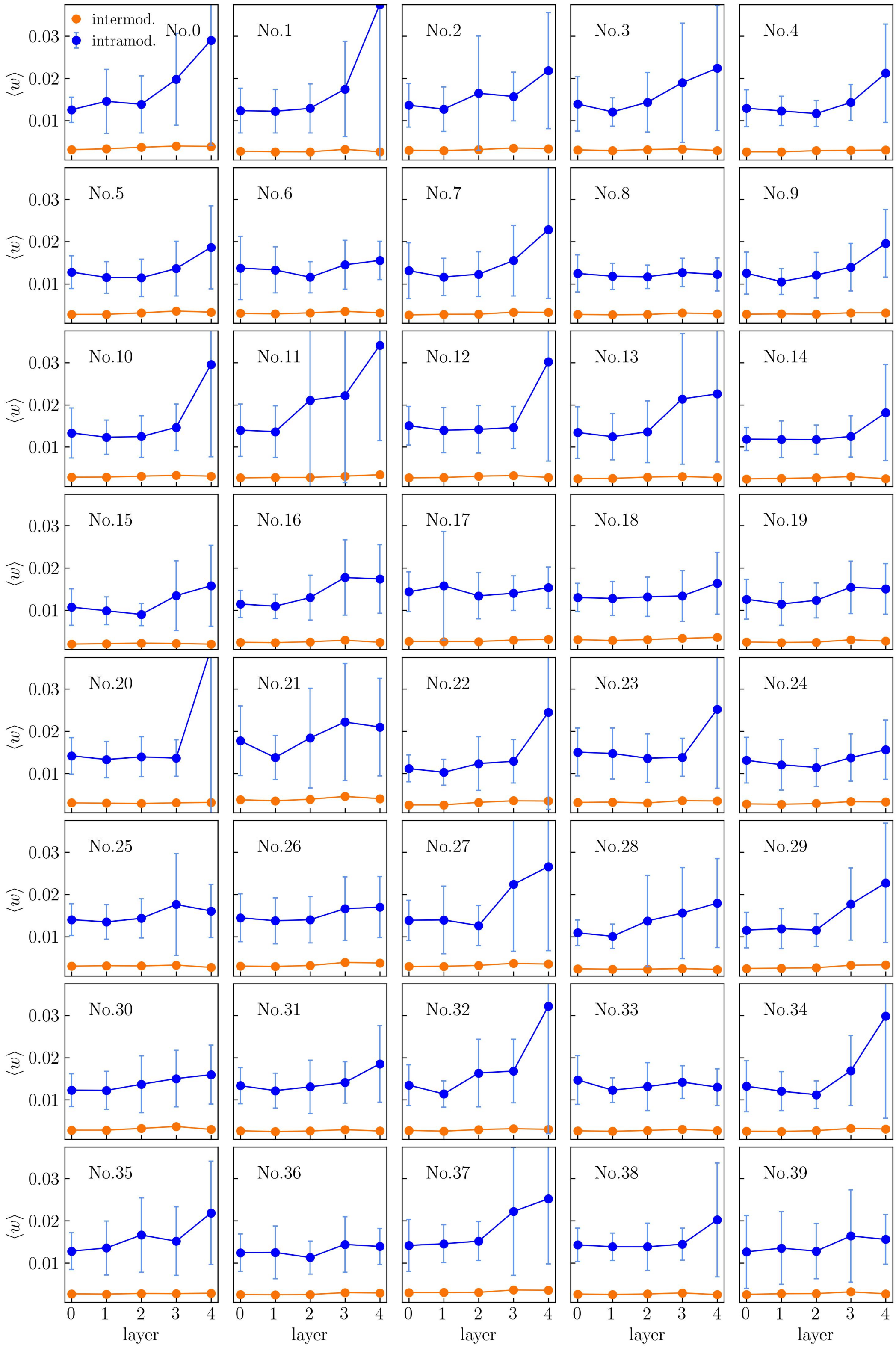

L: Average weight inside modules (intramodular links) compared to average weight outside modules (intermodular links), when modules are computed using the weighted Louvain algorithm. The average weight inside modules is computed as the mean of the average weights in the different modules.

IV. OTHER COMMUNITY DETECTION ALGORITHMS

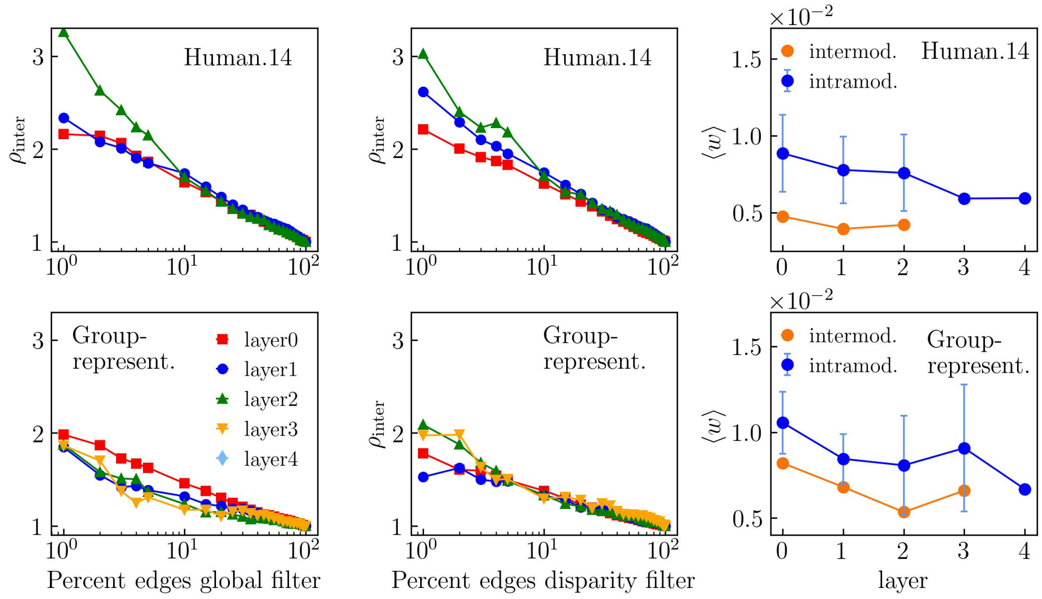

M: Weak ties spectra obtained using the Infomap algorithm.

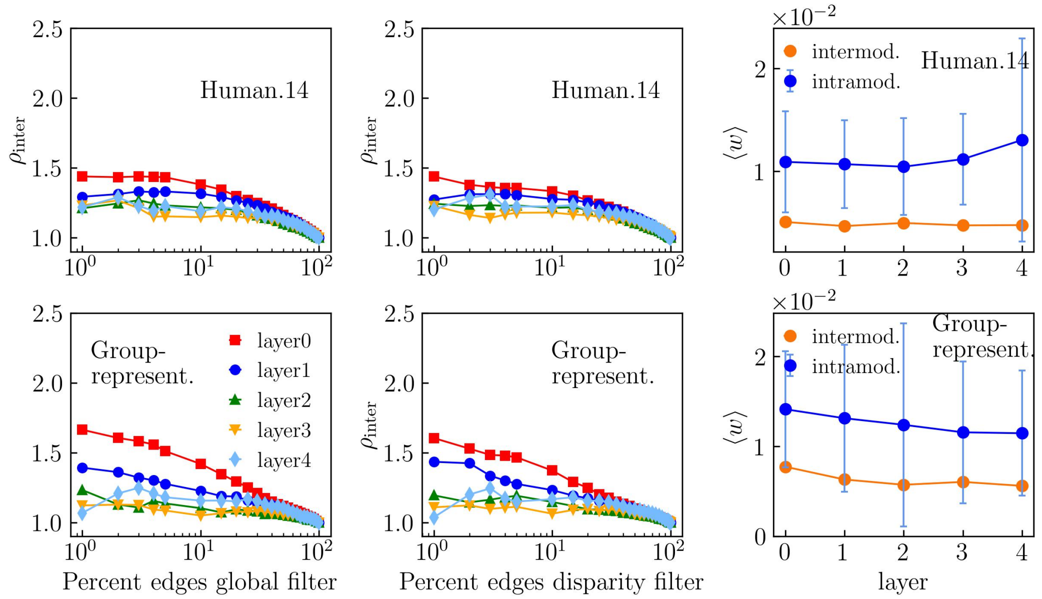

N: Weak ties spectra obtained using the SBM algorithm.

V. FIT OF THE MODEL

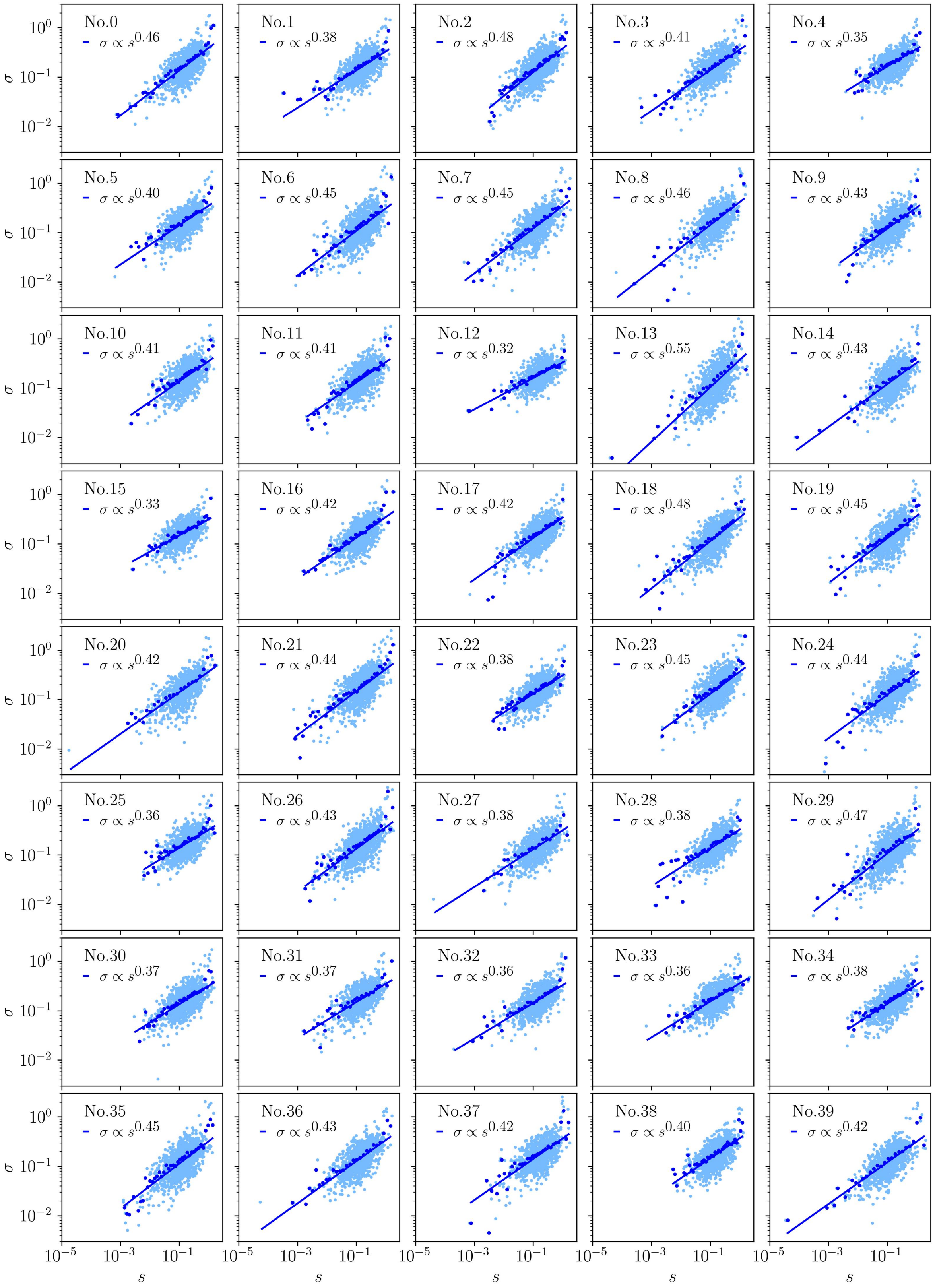

O: For all humans in the UL dataset, value of σ obtained for each node with strength s.

VI. GR MODEL

A. Flow of averages

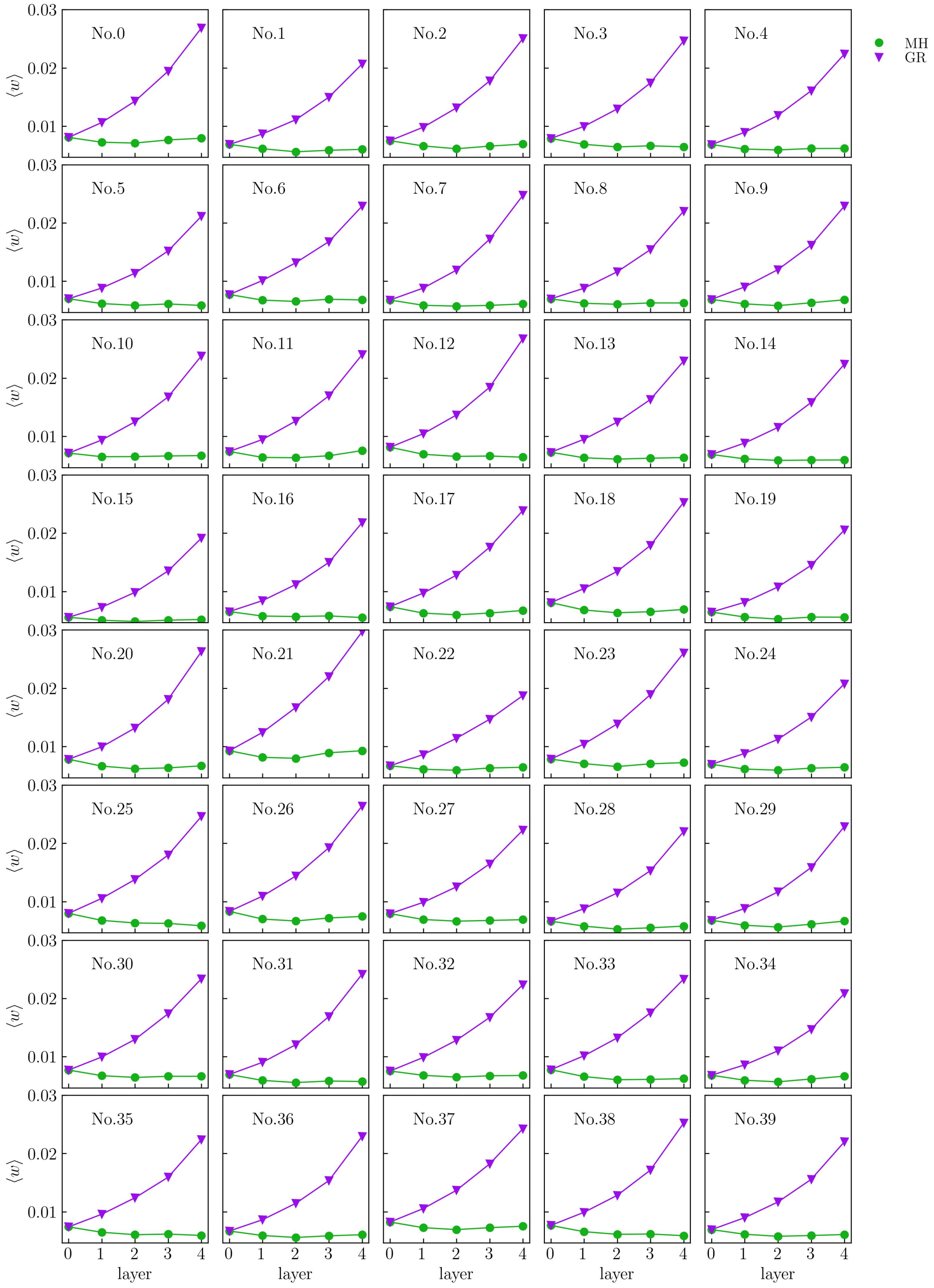

P: Comparison of the evolution of average weight in consecutive layers for the experimental data (in green) and the GR model (in purple).

B. Self-similarity

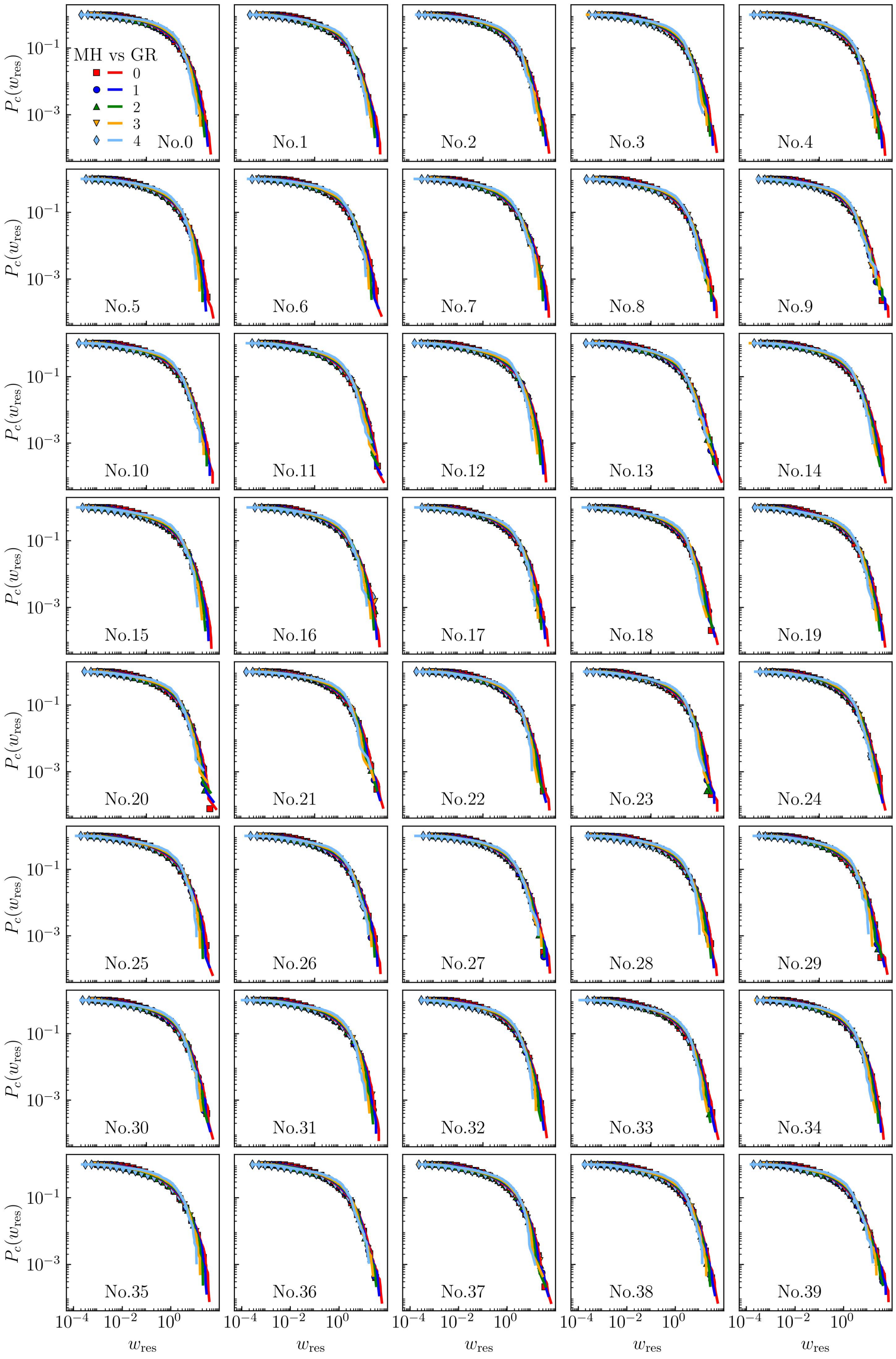

Q: Comparison of the complementary cumulative weight distributions obtained from the experimental data (points) and our model (solid lines) for the five layers considered. Results rescaled by the average weight of each layer.

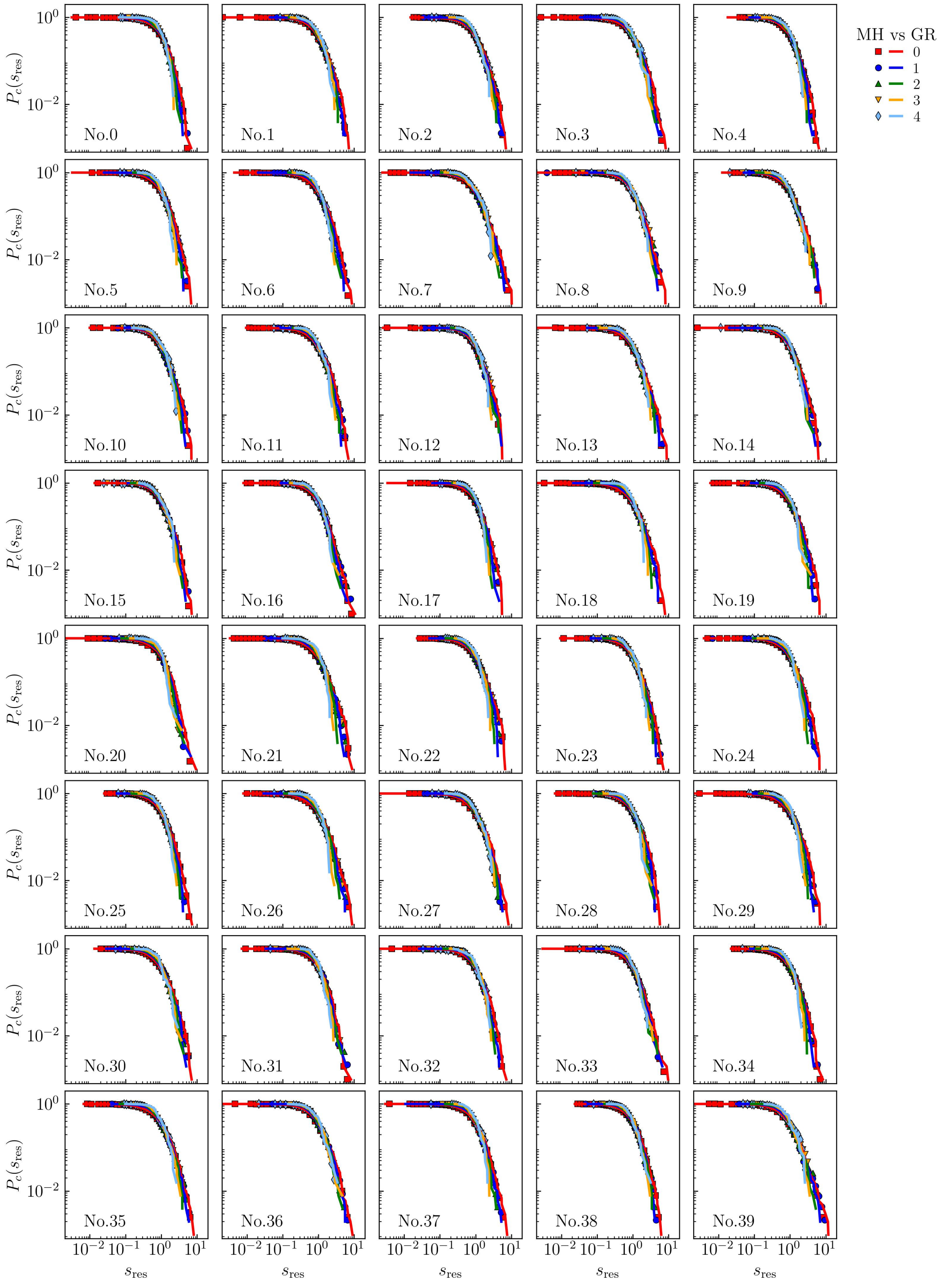

R: Comparison of the complementary cumulative strength distributions obtained from the experimental data (points) and our model (solid lines) for the five layers considered. Results rescaled by the average strength of each layer.

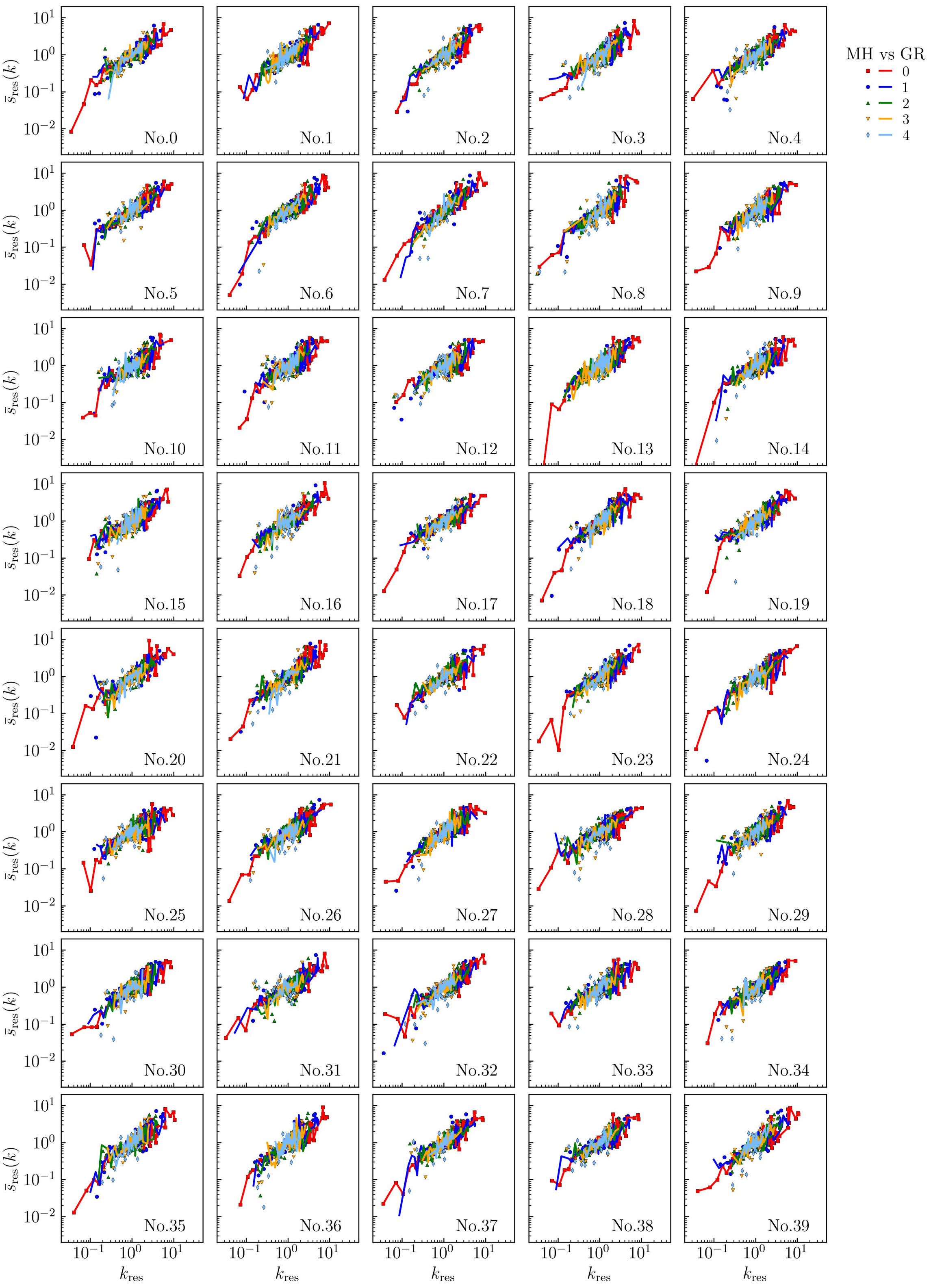

S: Comparison of the strength-degree relationship obtained from the experimental data (points) and our model (solid lines) for the five layers considered. Results rescaled by the average degree and strength of each layer.

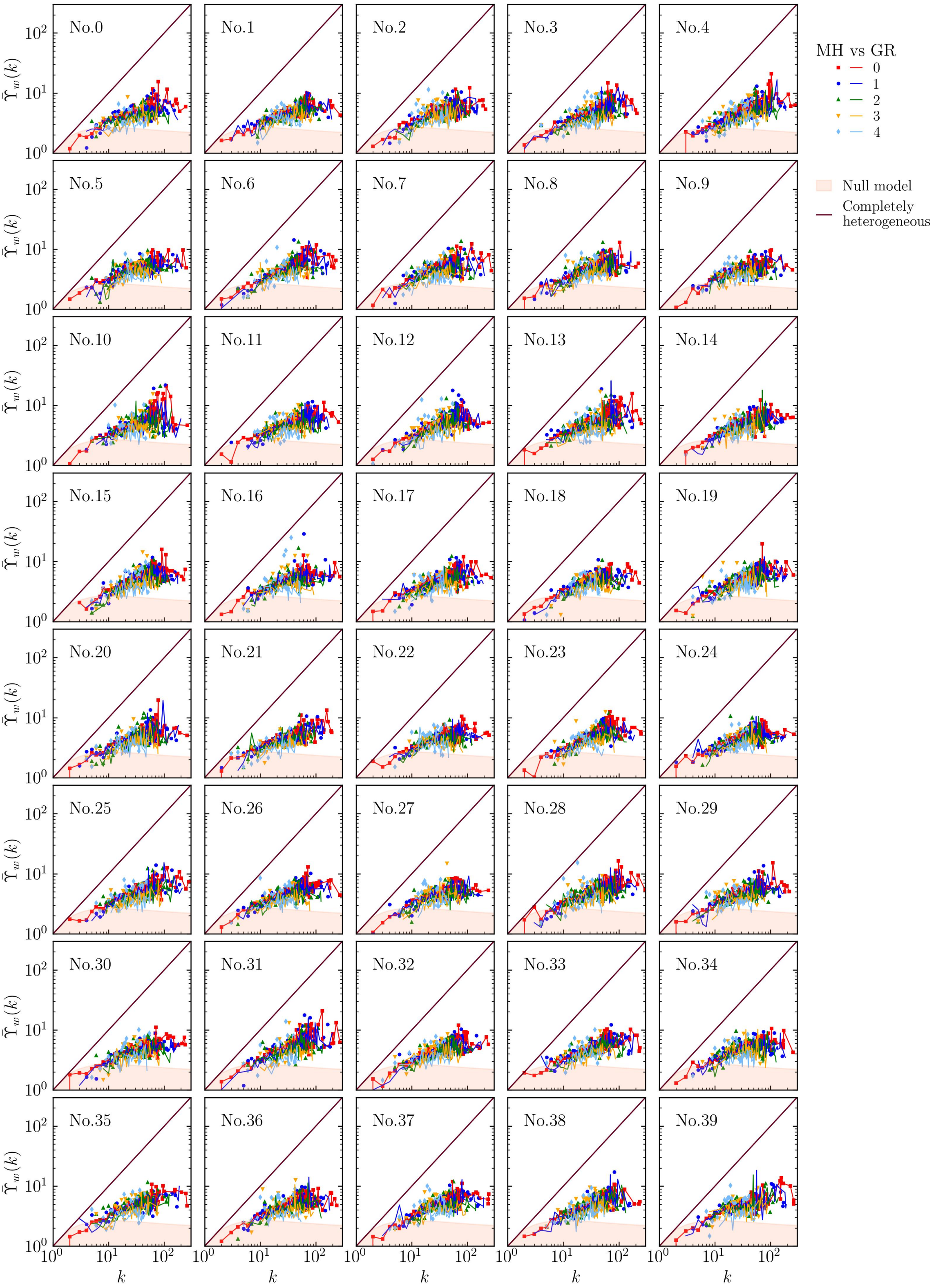

T: Comparison of the disparity measure obtained from the experimental data (points) and our model (solid lines) for the five layers considered.

C. Weak ties in GR model

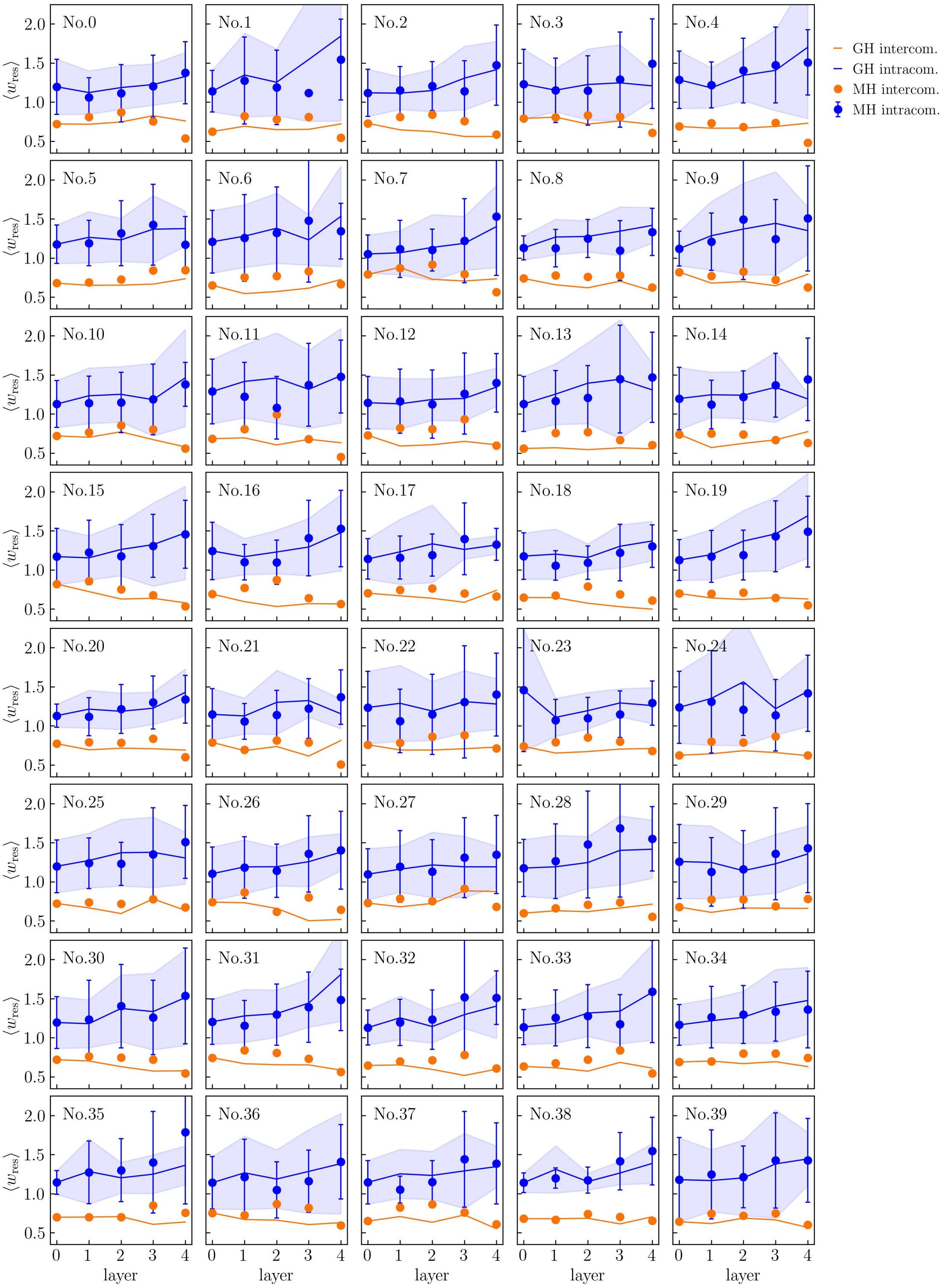

U: Comparison between results of the renormalized networks (lines and dashed area) and the experimental connectomes (dots and errorbars). Average weight inside modules (intramodular links) compared to average weight outside modules (intermodular links), when modules are computed using the Louvain algorithm. The average weight inside modules is computed as the mean of the average weights in the different modules. Results rescaled by the average weight of each layer.

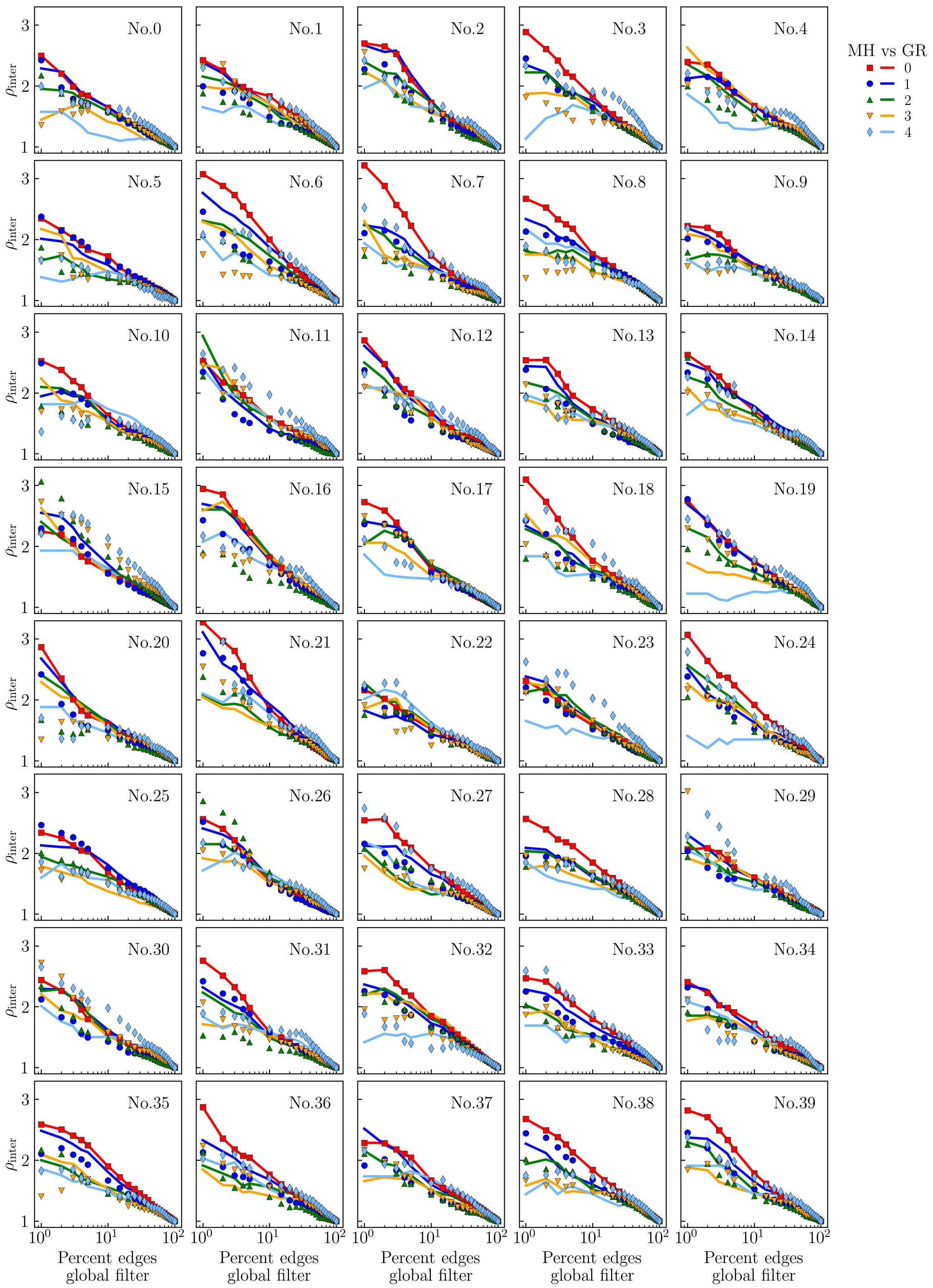

V: Comparison between results of the renormalized layers using the GR model (lines) and the experimental results (points). Normalized density of intermodular connections versus the percent of edges considered. The x axis X% means we are taking into account the X% of connections with lowest weight. X-axis in logscale.

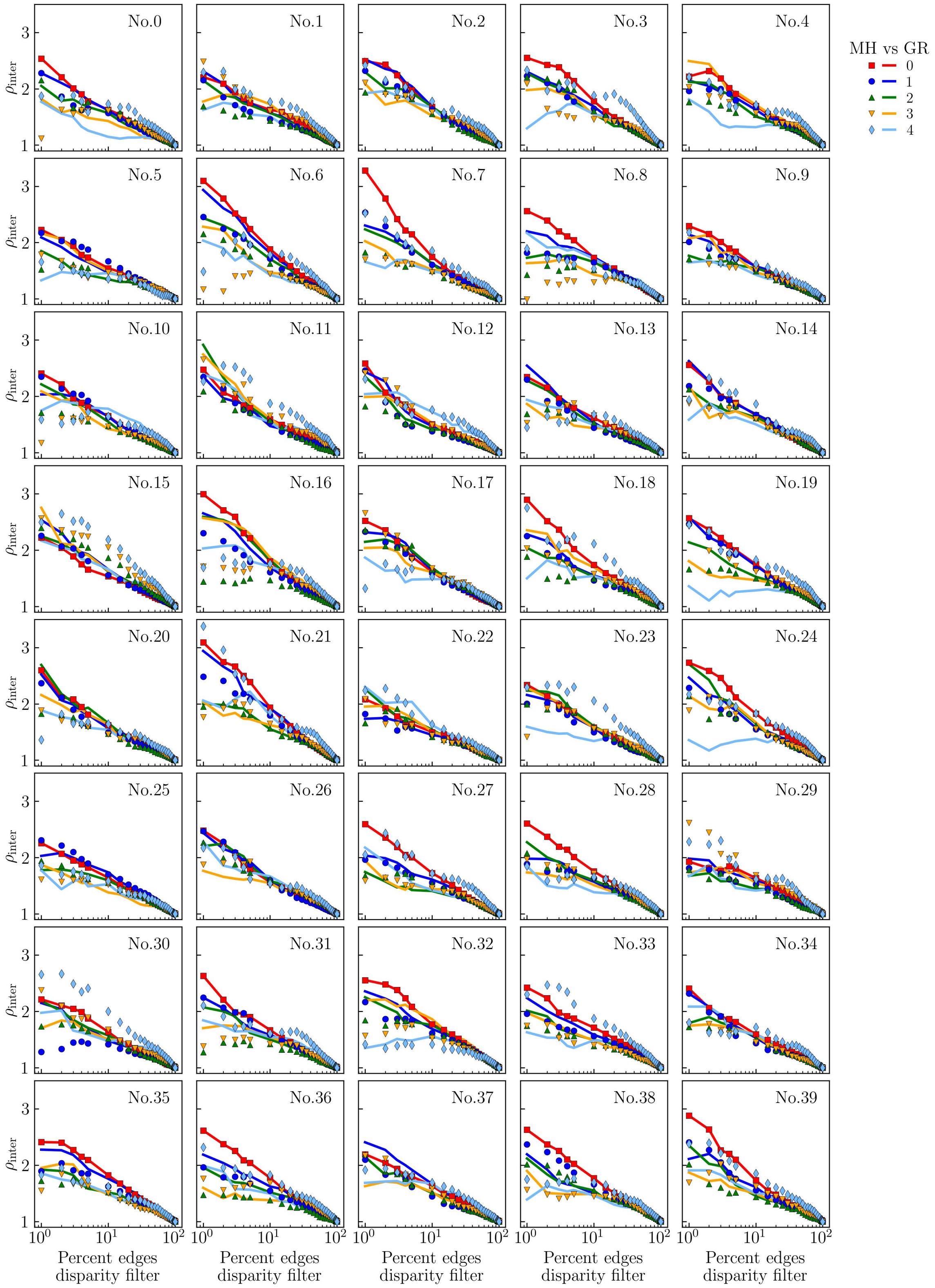

W: Comparison between results of the renormalized layers using the GR model (lines) and the experimental results (points). Normalized density of intermodular connections versus the percent of edges considered. The x axis X% means we are taking into account the X% of connections with lowest $1-\alpha$. X-axis in logscale.

VII. NULL MODELS

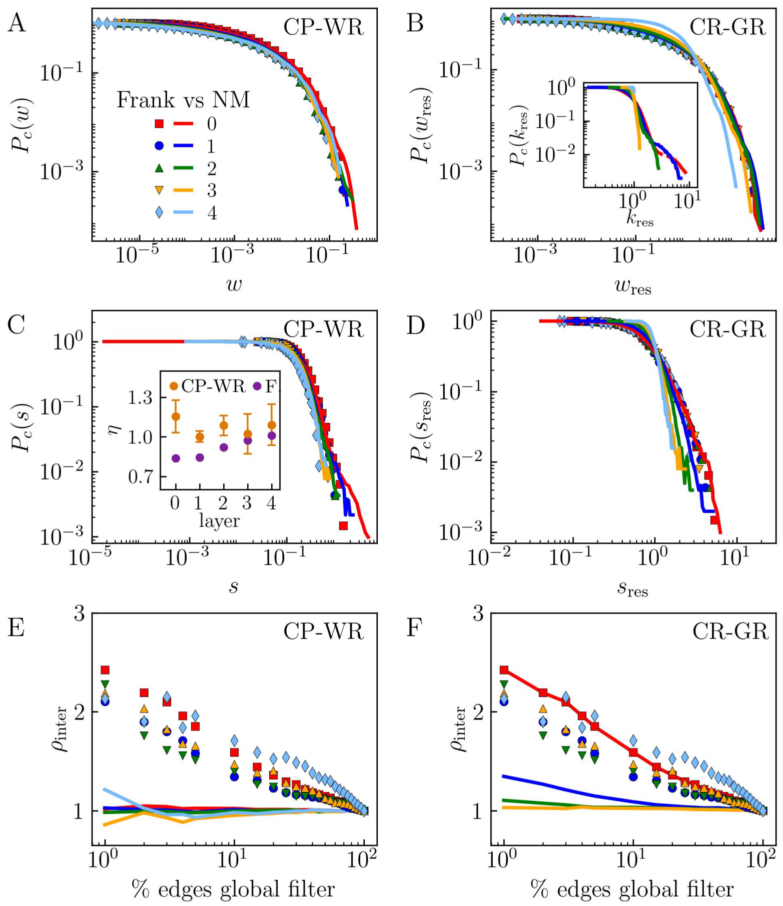

X: Null models for group-representative UL, here referred as Frankie. In all plots the dots correspond to experimental data and the curves to the null models. A, B) Complementary cumulative weight distributions. Inset in B: complementary cumulative degree distributions. C, D) Complementary cumulative strength distributions. Inset in C: exponent of the $s \sim k^{\eta}$ relationship. F states for the original group-representative. The mean and standard deviation of the ensemble is represented by the solid line and the shadowed area. E, F) Normalized density of intermodular links versus percent of edges considered by global thresholding.In the CR-GR, weight and strength have been rescaled by the average weight and strength of the respective layer.
