# Supplementary material for: The multiscale self-similarity of the weighted human brain connectome
Source: PLoS Comput Biol. 2025 Apr 7;21(4):e1012848. doi: 10.1371/journal.pcbi.1012848 (PMC11991287; doi:10.1371/journal.pcbi.1012848)
Supplement: S2 File — (PDF) [file pcbi.1012848.s002.pdf]

The multiscale self-similarity of the weighted human brain connectome

Supporting Information HCP Dataset

Laia Barjuan,[1,2] Muhua Zheng,[3] and M. Ángeles Serrano[1,2,4,*]

[1]*Departament de Física de la Matèria Condensada, Universitat de Barcelona, Martí i Franquès 1, Barcelona, Spain*
[2]*Universitat de Barcelona Institute of Complex Systems (UBICS), Universitat de Barcelona, Barcelona, Spain*
[3]*School of Physics and Electronic Engineering, Jiangsu University, Zhenjiang, Jiangsu, China*
[4]*ICREA, Passeig Lluís Companys 23, Barcelona, Spain*

CONTENTS

I. Flow of averages 6

II. Self-similarity 8

III. Weak ties hypothesis 13

IV. Frankie 18

V. GR Model 20
- A. Flow of averages 20
- B. Self-similarity 21
- C. Weak ties in GR model 25

VI. Null models 28

* marian.serrano@ub.edu

Here we present all results obtained for the 44 connectomes in the HCP Dataset. Regarding its multiscale structure, the following notation with respect to connectome layers is employed: layer 0 corresponds to the connectome with 1015 nodes, layer 1 to 463 nodes, layer 2 to 234 nodes, layer 3 to 129 nodes and layer 4 to 82 nodes.

Subject	layer	nodes	edges	$\langle k \rangle \pm$ SEM	$\langle w \rangle \pm$ SEM	$\langle s \rangle \pm$ SEM	η
	0	1014	37910	74.8 ± 1.5	0.00124 ± 0.00002	0.093 ± 0.002	0.74
	1	462	18024	78.0 ± 1.8	0.00100 ± 0.00002	0.078 ± 0.002	0.59
No. 0	2	233	8540	73.3 ± 1.8	0.00093 ± 0.00003	0.068 ± 0.003	0.58
	3	128	3857	60.3 ± 1.6	0.00105 ± 0.00006	0.063 ± 0.005	0.80
	4	82	1819	44.4 ± 1.5	0.00138 ± 0.00012	0.061 ± 0.006	0.82
	0	1014	39210	77.3 ± 1.7	0.00105 ± 0.00002	0.081 ± 0.001	0.73
	1	462	18776	81.3 ± 1.9	0.00084 ± 0.00002	0.068 ± 0.002	0.64
No. 1	2	233	8874	76.2 ± 1.9	0.00078 ± 0.00003	0.060 ± 0.003	0.65
	3	128	3985	62.3 ± 1.6	0.00089 ± 0.00005	0.055 ± 0.004	0.81
	4	82	1909	46.6 ± 1.4	0.00116 ± 0.00010	0.054 ± 0.006	1.02
	0	1014	39609	78.1 ± 1.5	0.00131 ± 0.00002	0.102 ± 0.002	0.66
	1	462	18603	80.5 ± 1.8	0.00106 ± 0.00002	0.086 ± 0.002	0.63
No. 2	2	233	8671	74.4 ± 1.7	0.00099 ± 0.00004	0.074 ± 0.003	0.71
	3	128	3785	59.1 ± 1.5	0.00113 ± 0.00006	0.067 ± 0.005	0.92
	4	82	1822	44.4 ± 1.4	0.00142 ± 0.00012	0.063 ± 0.006	1.05
	0	1014	40309	79.5 ± 1.7	0.00115 ± 0.00002	0.092 ± 0.002	0.72
	1	462	19158	82.9 ± 2.0	0.00093 ± 0.00002	0.077 ± 0.002	0.53
No. 3	2	233	9033	77.5 ± 1.9	0.00086 ± 0.00003	0.067 ± 0.003	0.56
	3	128	4038	63.1 ± 1.6	0.00098 ± 0.00006	0.062 ± 0.005	0.89
	4	82	1929	47.0 ± 1.5	0.00129 ± 0.00011	0.060 ± 0.006	0.93
	0	1014	43276	85.4 ± 1.7	0.00121 ± 0.00002	0.103 ± 0.002	0.78
	1	462	20470	88.6 ± 1.9	0.00099 ± 0.00002	0.088 ± 0.002	0.66
No. 4	2	233	9609	82.5 ± 1.8	0.00093 ± 0.00003	0.077 ± 0.003	0.96
	3	128	4428	69.2 ± 1.6	0.00101 ± 0.00006	0.070 ± 0.005	1.57
	4	82	2102	51.3 ± 1.5	0.00130 ± 0.00011	0.067 ± 0.007	1.83
	0	1014	40165	79.2 ± 1.5	0.00097 ± 0.00001	0.077 ± 0.001	0.62
	1	462	19100	82.7 ± 1.8	0.00077 ± 0.00002	0.064 ± 0.002	0.58
No. 5	2	233	9093	78.1 ± 1.7	0.00071 ± 0.00003	0.055 ± 0.002	0.70
	3	128	4075	63.7 ± 1.5	0.00079 ± 0.00005	0.051 ± 0.004	1.07
	4	82	1945	47.4 ± 1.4	0.00102 ± 0.00009	0.048 ± 0.005	1.21
	0	1014	39638	78.2 ± 1.6	0.00126 ± 0.00002	0.099 ± 0.002	0.71
	1	462	18692	80.9 ± 1.8	0.00103 ± 0.00002	0.083 ± 0.002	0.60
No. 6	2	233	8821	75.7 ± 1.7	0.00095 ± 0.00003	0.072 ± 0.003	0.85
	3	128	3952	61.8 ± 1.6	0.00107 ± 0.00006	0.066 ± 0.005	1.31
	4	82	1895	46.2 ± 1.4	0.00137 ± 0.00011	0.063 ± 0.006	1.65
	0	1014	44089	87.0 ± 1.7	0.00113 ± 0.00002	0.098 ± 0.002	0.62
	1	462	20383	88.2 ± 1.9	0.00094 ± 0.00002	0.083 ± 0.002	0.62
No. 7	2	233	9312	79.9 ± 1.8	0.00091 ± 0.00003	0.073 ± 0.003	0.67
	3	128	4182	65.3 ± 1.6	0.00101 ± 0.00006	0.066 ± 0.005	1.07
	4	82	2010	49.0 ± 1.4	0.00130 ± 0.00010	0.064 ± 0.006	1.24
	0	1014	36557	72.1 ± 1.6	0.00172 ± 0.00002	0.124 ± 0.003	0.81
	1	462	17316	75.0 ± 1.8	0.00143 ± 0.00003	0.107 ± 0.003	0.78
No. 8	2	233	8296	71.2 ± 1.8	0.00134 ± 0.00005	0.096 ± 0.004	0.82
	3	128	3745	58.5 ± 1.6	0.00151 ± 0.00009	0.088 ± 0.007	1.01
	4	82	1828	44.6 ± 1.5	0.00191 ± 0.00016	0.085 ± 0.009	1.10
	0	1014	38249	75.4 ± 1.6	0.00153 ± 0.00002	0.115 ± 0.002	0.82
	1	462	18352	79.4 ± 1.9	0.00125 ± 0.00003	0.099 ± 0.003	0.84
No. 9	2	233	8702	74.7 ± 1.9	0.00116 ± 0.00004	0.087 ± 0.004	0.74
	3	128	3955	61.8 ± 1.7	0.00127 ± 0.00007	0.079 ± 0.005	0.94
	4	82	1893	46.2 ± 1.6	0.00161 ± 0.00012	0.074 ± 0.007	1.03
	0	1014	39578	78.1 ± 1.5	0.00085 ± 0.00001	0.067 ± 0.001	0.58
	1	462	18409	79.7 ± 1.7	0.00069 ± 0.00002	0.055 ± 0.001	0.54
No. 10	2	233	8586	73.7 ± 1.7	0.00064 ± 0.00002	0.047 ± 0.002	0.64
	3	128	3767	58.9 ± 1.5	0.00072 ± 0.00004	0.043 ± 0.003	0.95
	4	82	1785	43.5 ± 1.4	0.00093 ± 0.00008	0.041 ± 0.004	1.11

Subject	layer	nodes	edges	$\langle k \rangle \pm$ SEM	$\langle w \rangle \pm$ SEM	$\langle s \rangle \pm$ SEM	η
No. 11	0	1013	34436	68.0 ± 1.3	0.00136 ± 0.00002	0.093 ± 0.002	0.72
	1	462	16289	70.5 ± 1.5	0.00110 ± 0.00002	0.077 ± 0.002	0.57
	2	233	7718	66.2 ± 1.5	0.00100 ± 0.00004	0.066 ± 0.003	0.60
	3	128	3521	55.0 ± 1.4	0.00107 ± 0.00006	0.059 ± 0.004	0.84
	4	82	1698	41.4 ± 1.4	0.00136 ± 0.00011	0.057 ± 0.006	0.90
No. 12	0	1014	39631	78.2 ± 1.5	0.00140 ± 0.00002	0.109 ± 0.002	0.77
	1	462	18863	81.7 ± 1.7	0.00111 ± 0.00002	0.091 ± 0.002	0.57
	2	233	8793	75.5 ± 1.7	0.00103 ± 0.00004	0.078 ± 0.003	0.60
	3	128	3956	61.8 ± 1.6	0.00110 ± 0.00006	0.068 ± 0.005	0.92
	4	82	1900	46.3 ± 1.5	0.00137 ± 0.00011	0.063 ± 0.006	1.25
No. 13	0	1014	40358	79.6 ± 1.7	0.00098 ± 0.00001	0.078 ± 0.001	0.71
	1	462	19173	83.0 ± 1.9	0.00078 ± 0.00002	0.065 ± 0.002	0.67
	2	233	9043	77.6 ± 1.8	0.00074 ± 0.00003	0.057 ± 0.003	0.74
	3	128	4012	62.7 ± 1.7	0.00085 ± 0.00005	0.053 ± 0.004	0.96
	4	82	1928	47.0 ± 1.5	0.00113 ± 0.00010	0.053 ± 0.006	1.30
No. 14	0	1014	40821	80.5 ± 1.6	0.00113 ± 0.00002	0.091 ± 0.002	0.68
	1	462	19050	82.5 ± 1.8	0.00092 ± 0.00002	0.076 ± 0.002	0.52
	2	233	8767	75.3 ± 1.7	0.00086 ± 0.00003	0.065 ± 0.002	0.55
	3	128	3924	61.3 ± 1.6	0.00096 ± 0.00005	0.059 ± 0.004	0.72
	4	82	1876	45.8 ± 1.5	0.00120 ± 0.00010	0.055 ± 0.005	0.86
No. 15	0	1014	37896	74.7 ± 1.6	0.00100 ± 0.00001	0.075 ± 0.001	0.65
	1	462	18119	78.4 ± 1.8	0.00079 ± 0.00002	0.062 ± 0.002	0.67
	2	233	8732	75.0 ± 1.8	0.00072 ± 0.00003	0.054 ± 0.002	0.84
	3	128	3982	62.2 ± 1.6	0.00081 ± 0.00005	0.050 ± 0.004	1.14
	4	82	1969	48.0 ± 1.5	0.00103 ± 0.00008	0.049 ± 0.005	1.43
No. 16	0	1014	39589	78.1 ± 1.6	0.00096 ± 0.00001	0.075 ± 0.001	0.73
	1	462	18938	82.0 ± 1.8	0.00076 ± 0.00002	0.063 ± 0.002	0.61
	2	233	9016	77.4 ± 1.8	0.00070 ± 0.00003	0.054 ± 0.002	0.76
	3	128	4026	62.9 ± 1.5	0.00080 ± 0.00005	0.050 ± 0.004	1.03
	4	82	1934	47.2 ± 1.3	0.00102 ± 0.00009	0.048 ± 0.005	1.11
No. 17	0	1014	38009	75.0 ± 1.6	0.00143 ± 0.00002	0.107 ± 0.002	0.75
	1	462	17734	76.8 ± 1.8	0.00118 ± 0.00003	0.091 ± 0.002	0.65
	2	233	8280	71.1 ± 1.8	0.00112 ± 0.00004	0.080 ± 0.003	0.69
	3	128	3810	59.5 ± 1.5	0.00121 ± 0.00007	0.072 ± 0.005	1.31
	4	82	1871	45.6 ± 1.4	0.00150 ± 0.00012	0.068 ± 0.007	1.48
No. 18	0	1014	46070	90.9 ± 1.7	0.00086 ± 0.00001	0.078 ± 0.001	0.59
	1	462	21730	94.1 ± 1.9	0.00070 ± 0.00002	0.066 ± 0.002	0.61
	2	233	10018	86.0 ± 1.8	0.00067 ± 0.00002	0.057 ± 0.002	0.65
	3	128	4407	68.9 ± 1.5	0.00076 ± 0.00004	0.052 ± 0.004	1.03
	4	82	2080	50.7 ± 1.3	0.00098 ± 0.00008	0.050 ± 0.005	1.14
No. 19	0	1014	41676	82.2 ± 1.6	0.00118 ± 0.00002	0.097 ± 0.002	0.74
	1	462	19773	85.6 ± 1.9	0.00097 ± 0.00002	0.083 ± 0.002	0.50
	2	233	9225	79.2 ± 1.8	0.00090 ± 0.00003	0.072 ± 0.003	0.71
	3	128	4224	66.0 ± 1.6	0.00098 ± 0.00005	0.065 ± 0.004	1.12
	4	82	2036	49.7 ± 1.5	0.00123 ± 0.00010	0.061 ± 0.006	1.39
No. 20	0	1013	41727	82.4 ± 1.6	0.00141 ± 0.00002	0.116 ± 0.002	0.81
	1	462	19757	85.5 ± 1.8	0.00117 ± 0.00003	0.100 ± 0.002	0.68
	2	233	9241	79.3 ± 1.7	0.00108 ± 0.00004	0.086 ± 0.003	0.62
	3	128	4074	63.7 ± 1.5	0.00121 ± 0.00007	0.077 ± 0.005	0.89
	4	82	1933	47.1 ± 1.4	0.00152 ± 0.00012	0.072 ± 0.007	0.99
No. 21	0	1014	36659	72.3 ± 1.6	0.00146 ± 0.00002	0.106 ± 0.002	0.68
	1	462	17485	75.7 ± 1.8	0.00118 ± 0.00003	0.089 ± 0.002	0.64
	2	233	8392	72.0 ± 1.8	0.00108 ± 0.00004	0.078 ± 0.003	0.74
	3	128	3874	60.5 ± 1.5	0.00116 ± 0.00007	0.070 ± 0.005	1.02
	4	82	1932	47.1 ± 1.4	0.00140 ± 0.00012	0.066 ± 0.007	1.30
No. 22	0	1014	38855	76.6 ± 1.5	0.00081 ± 0.00001	0.062 ± 0.001	0.70
	1	462	18783	81.3 ± 1.7	0.00063 ± 0.00002	0.051 ± 0.001	0.58
	2	233	9069	77.8 ± 1.7	0.00057 ± 0.00002	0.045 ± 0.002	0.66
	3	128	3966	62.0 ± 1.5	0.00065 ± 0.00004	0.040 ± 0.003	0.92
	4	82	1866	45.5 ± 1.5	0.00085 ± 0.00007	0.039 ± 0.004	1.19

Subject	layer	nodes	edges	$\langle k \rangle \pm$ SEM	$\langle w \rangle \pm$ SEM	$\langle s \rangle \pm$ SEM	η
No. 23	0	1014	37235	73.4 ± 1.4	0.00107 ± 0.00002	0.078 ± 0.001	0.64
	1	462	17532	75.9 ± 1.6	0.00085 ± 0.00002	0.065 ± 0.001	0.57
	2	233	8467	72.7 ± 1.6	0.00075 ± 0.00003	0.055 ± 0.002	0.80
	3	128	3882	60.7 ± 1.4	0.00081 ± 0.00005	0.049 ± 0.003	1.14
	4	82	1878	45.8 ± 1.2	0.00101 ± 0.00009	0.046 ± 0.005	1.17
No. 24	0	1014	38407	75.8 ± 1.5	0.00112 ± 0.00002	0.085 ± 0.001	0.65
	1	462	18295	79.2 ± 1.7	0.00090 ± 0.00002	0.071 ± 0.002	0.61
	2	233	8666	74.4 ± 1.7	0.00083 ± 0.00003	0.062 ± 0.003	0.76
	3	128	3907	61.0 ± 1.5	0.00095 ± 0.00005	0.058 ± 0.004	1.13
	4	82	1862	45.4 ± 1.3	0.00124 ± 0.00010	0.056 ± 0.006	1.25
No. 25	0	1014	43631	86.1 ± 1.7	0.00113 ± 0.00002	0.097 ± 0.002	0.67
	1	462	20366	88.2 ± 1.8	0.00093 ± 0.00002	0.082 ± 0.002	0.50
	2	233	9329	80.1 ± 1.8	0.00088 ± 0.00003	0.071 ± 0.003	0.70
	3	128	4138	64.7 ± 1.6	0.00098 ± 0.00005	0.063 ± 0.004	1.04
	4	82	2005	48.9 ± 1.5	0.00123 ± 0.00010	0.060 ± 0.006	1.46
No. 26	0	1014	42351	83.5 ± 1.6	0.00120 ± 0.00002	0.100 ± 0.002	0.80
	1	462	20154	87.2 ± 1.8	0.00097 ± 0.00002	0.085 ± 0.002	0.59
	2	233	9465	81.2 ± 1.7	0.00091 ± 0.00003	0.074 ± 0.003	0.77
	3	128	4193	65.5 ± 1.5	0.00102 ± 0.00006	0.067 ± 0.005	0.98
	4	82	2038	49.7 ± 1.4	0.00127 ± 0.00010	0.063 ± 0.006	1.50
No. 27	0	1014	37875	74.7 ± 1.5	0.00108 ± 0.00002	0.080 ± 0.001	0.54
	1	462	17836	77.2 ± 1.7	0.00087 ± 0.00002	0.067 ± 0.002	0.58
	2	233	8418	72.3 ± 1.6	0.00079 ± 0.00003	0.057 ± 0.002	0.75
	3	128	3810	59.5 ± 1.4	0.00086 ± 0.00005	0.051 ± 0.004	1.11
	4	82	1828	44.6 ± 1.3	0.00110 ± 0.00009	0.049 ± 0.005	1.80
No. 28	0	1014	35737	70.5 ± 1.5	0.00130 ± 0.00002	0.092 ± 0.002	0.80
	1	462	16984	73.5 ± 1.7	0.00105 ± 0.00002	0.077 ± 0.002	0.75
	2	233	8227	70.6 ± 1.7	0.00096 ± 0.00004	0.068 ± 0.003	0.68
	3	128	3704	57.9 ± 1.6	0.00108 ± 0.00006	0.063 ± 0.005	0.83
	4	82	1789	43.6 ± 1.5	0.00138 ± 0.00011	0.060 ± 0.006	1.07
No. 29	0	1014	39125	77.2 ± 1.5	0.00126 ± 0.00002	0.097 ± 0.002	0.80
	1	462	18508	80.1 ± 1.7	0.00104 ± 0.00002	0.083 ± 0.002	0.59
	2	233	8648	74.2 ± 1.6	0.00099 ± 0.00004	0.073 ± 0.003	0.85
	3	128	3894	60.8 ± 1.4	0.00111 ± 0.00006	0.067 ± 0.005	1.69
	4	82	1893	46.2 ± 1.3	0.00140 ± 0.00011	0.065 ± 0.006	2.30
No. 30	0	1014	41041	80.9 ± 1.6	0.00129 ± 0.00002	0.104 ± 0.002	0.72
	1	462	19264	83.4 ± 1.8	0.00106 ± 0.00002	0.088 ± 0.002	0.68
	2	233	9012	77.4 ± 1.7	0.00099 ± 0.00004	0.076 ± 0.003	0.78
	3	128	4083	63.8 ± 1.5	0.00108 ± 0.00006	0.069 ± 0.005	0.96
	4	82	1973	48.1 ± 1.4	0.00138 ± 0.00012	0.067 ± 0.007	0.99
No. 31	0	1014	39813	78.5 ± 1.6	0.00100 ± 0.00001	0.079 ± 0.001	0.61
	1	462	18826	81.5 ± 1.8	0.00081 ± 0.00002	0.066 ± 0.002	0.58
	2	233	8861	76.1 ± 1.8	0.00075 ± 0.00003	0.057 ± 0.002	0.47
	3	128	3914	61.2 ± 1.6	0.00084 ± 0.00005	0.051 ± 0.004	0.62
	4	82	1827	44.6 ± 1.5	0.00111 ± 0.00010	0.050 ± 0.005	0.66
No. 32	0	1013	39161	77.3 ± 1.6	0.00087 ± 0.00001	0.067 ± 0.001	0.66
	1	462	18393	79.6 ± 1.8	0.00070 ± 0.00002	0.056 ± 0.001	0.58
	2	233	8649	74.2 ± 1.8	0.00065 ± 0.00002	0.048 ± 0.002	0.69
	3	128	3759	58.7 ± 1.6	0.00075 ± 0.00005	0.044 ± 0.003	1.41
	4	82	1819	44.4 ± 1.4	0.00097 ± 0.00009	0.043 ± 0.005	1.97
No. 33	0	1014	40400	79.7 ± 1.6	0.00101 ± 0.00001	0.080 ± 0.001	0.74
	1	462	19127	82.8 ± 1.8	0.00081 ± 0.00002	0.067 ± 0.002	0.63
	2	233	9119	78.3 ± 1.8	0.00074 ± 0.00003	0.058 ± 0.002	0.85
	3	128	4019	62.8 ± 1.6	0.00083 ± 0.00005	0.052 ± 0.004	1.28
	4	82	1913	46.7 ± 1.4	0.00105 ± 0.00009	0.049 ± 0.005	1.50
No. 34	0	1014	44274	87.3 ± 1.7	0.00108 ± 0.00002	0.094 ± 0.002	0.76
	1	462	20590	89.1 ± 1.9	0.00089 ± 0.00002	0.080 ± 0.002	0.63
	2	233	9377	80.5 ± 1.8	0.00087 ± 0.00003	0.070 ± 0.003	0.75
	3	128	4224	66.0 ± 1.5	0.00096 ± 0.00005	0.063 ± 0.004	1.09
	4	82	2035	49.6 ± 1.4	0.00121 ± 0.00009	0.060 ± 0.006	1.54

Subject	layer	nodes	edges	$\langle k \rangle \pm$ SEM	$\langle w \rangle \pm$ SEM	$\langle s \rangle \pm$ SEM	η
	0	1014	46025	90.8 ± 1.7	0.00099 ± 0.00001	0.090 ± 0.002	0.73
	1	462	21758	94.2 ± 1.9	0.00081 ± 0.00002	0.077 ± 0.002	0.69
No. 35	2	233	10004	85.9 ± 1.8	0.00078 ± 0.00003	0.067 ± 0.003	0.77
	3	128	4385	68.5 ± 1.5	0.00090 ± 0.00005	0.062 ± 0.004	1.30
	4	82	2017	49.2 ± 1.4	0.00121 ± 0.00010	0.060 ± 0.006	1.55
	0	1014	37237	73.4 ± 1.5	0.00086 ± 0.00001	0.063 ± 0.001	0.68
	1	462	18008	78.0 ± 1.7	0.00067 ± 0.00002	0.052 ± 0.001	0.68
No. 36	2	233	8642	74.2 ± 1.7	0.00060 ± 0.00002	0.045 ± 0.002	0.77
	3	128	3776	59.0 ± 1.6	0.00068 ± 0.00004	0.040 ± 0.003	0.96
	4	82	1781	43.4 ± 1.5	0.00088 ± 0.00007	0.038 ± 0.004	1.14
	0	1014	45512	89.8 ± 1.8	0.00086 ± 0.00001	0.077 ± 0.001	0.65
	1	462	21300	92.2 ± 1.9	0.00071 ± 0.00002	0.066 ± 0.002	0.56
No. 37	2	233	9777	83.9 ± 1.8	0.00069 ± 0.00002	0.058 ± 0.002	0.69
	3	128	4281	66.9 ± 1.5	0.00080 ± 0.00005	0.054 ± 0.004	0.95
	4	82	2020	49.3 ± 1.3	0.00107 ± 0.00009	0.053 ± 0.005	1.19
	0	1014	40209	79.3 ± 1.6	0.00101 ± 0.00001	0.080 ± 0.001	0.74
	1	462	19094	82.7 ± 1.8	0.00081 ± 0.00002	0.067 ± 0.002	0.73
No. 38	2	233	9038	77.6 ± 1.7	0.00075 ± 0.00003	0.059 ± 0.003	0.73
	3	128	3985	62.3 ± 1.6	0.00084 ± 0.00005	0.052 ± 0.004	0.94
	4	82	1892	46.1 ± 1.4	0.00111 ± 0.00009	0.051 ± 0.006	1.00
	0	1014	34947	68.9 ± 1.5	0.00113 ± 0.00002	0.078 ± 0.001	0.76
	1	462	16844	72.9 ± 1.8	0.00089 ± 0.00002	0.065 ± 0.002	0.72
No. 39	2	233	8120	69.7 ± 1.8	0.00082 ± 0.00003	0.057 ± 0.003	0.72
	3	128	3674	57.4 ± 1.6	0.00092 ± 0.00005	0.053 ± 0.004	0.79
	4	82	1818	44.3 ± 1.5	0.00117 ± 0.00010	0.052 ± 0.006	0.98
	0	1014	38384	75.7 ± 1.5	0.00136 ± 0.00002	0.103 ± 0.002	0.72
	1	462	18201	78.8 ± 1.8	0.00112 ± 0.00002	0.088 ± 0.002	0.66
No. 40	2	233	8652	74.3 ± 1.8	0.00106 ± 0.00004	0.078 ± 0.003	0.65
	3	128	3886	60.7 ± 1.6	0.00119 ± 0.00007	0.072 ± 0.005	0.70
	4	82	1896	46.2 ± 1.5	0.00151 ± 0.00012	0.070 ± 0.007	0.80
	0	1014	35161	69.4 ± 1.5	0.00131 ± 0.00002	0.091 ± 0.002	0.70
	1	462	16812	72.8 ± 1.7	0.00104 ± 0.00002	0.076 ± 0.002	0.73
No. 41	2	233	8086	69.4 ± 1.7	0.00095 ± 0.00004	0.066 ± 0.003	0.70
	3	128	3734	58.3 ± 1.6	0.00104 ± 0.00006	0.061 ± 0.005	0.97
	4	82	1833	44.7 ± 1.5	0.00131 ± 0.00011	0.058 ± 0.006	1.15
	0	1014	40731	80.3 ± 1.5	0.00122 ± 0.00002	0.098 ± 0.002	0.75
	1	462	19399	84.0 ± 1.7	0.00099 ± 0.00002	0.083 ± 0.002	0.66
No. 42	2	233	9190	78.9 ± 1.7	0.00093 ± 0.00003	0.074 ± 0.003	0.78
	3	128	4132	64.6 ± 1.5	0.00104 ± 0.00006	0.067 ± 0.005	0.92
	4	82	1998	48.7 ± 1.4	0.00136 ± 0.00011	0.066 ± 0.007	1.13
	0	1014	37845	74.6 ± 1.5	0.00112 ± 0.00002	0.084 ± 0.001	0.61
	1	462	17927	77.6 ± 1.7	0.00090 ± 0.00002	0.070 ± 0.002	0.56
No. 43	2	233	8447	72.5 ± 1.7	0.00083 ± 0.00003	0.060 ± 0.002	0.62
	3	128	3841	60.0 ± 1.5	0.00090 ± 0.00005	0.054 ± 0.004	0.84
	4	82	1838	44.8 ± 1.4	0.00113 ± 0.00010	0.050 ± 0.005	1.06
	0	1014	38771	76.5 ± 1.7	0.00128 ± 0.00002	0.098 ± 0.001	0.60
	1	462	18702	81.0 ± 1.9	0.00097 ± 0.00002	0.079 ± 0.002	0.64
Frankie	2	233	8846	75.9 ± 1.8	0.00087 ± 0.00003	0.066 ± 0.003	0.75
	3	128	4006	62.6 ± 1.6	0.00097 ± 0.00006	0.061 ± 0.004	0.95
	4	82	1975	48.2 ± 1.5	0.00118 ± 0.00010	0.057 ± 0.006	1.11

A: Overview of the 44 connectomes in the HCP dataset. Number of nodes, number of edges, average degree, average weight, average strength and corresponding ±1 standard error interval around the mean (SEM), and exponent of the strength-degree power-law relationship, $s \propto k^{\eta}$.

I. FLOW OF AVERAGES

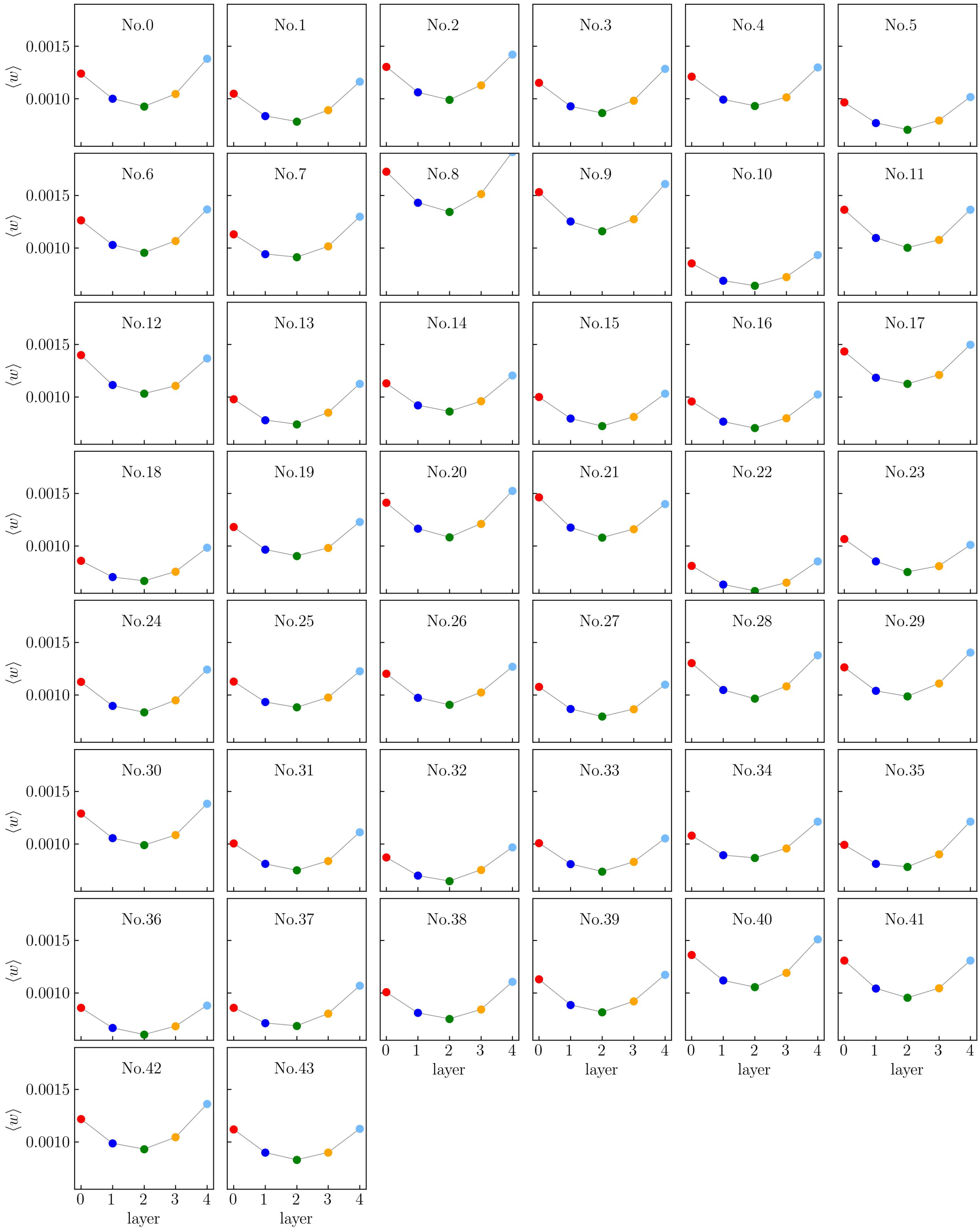

A: Evolution of average weight in consecutive layers.

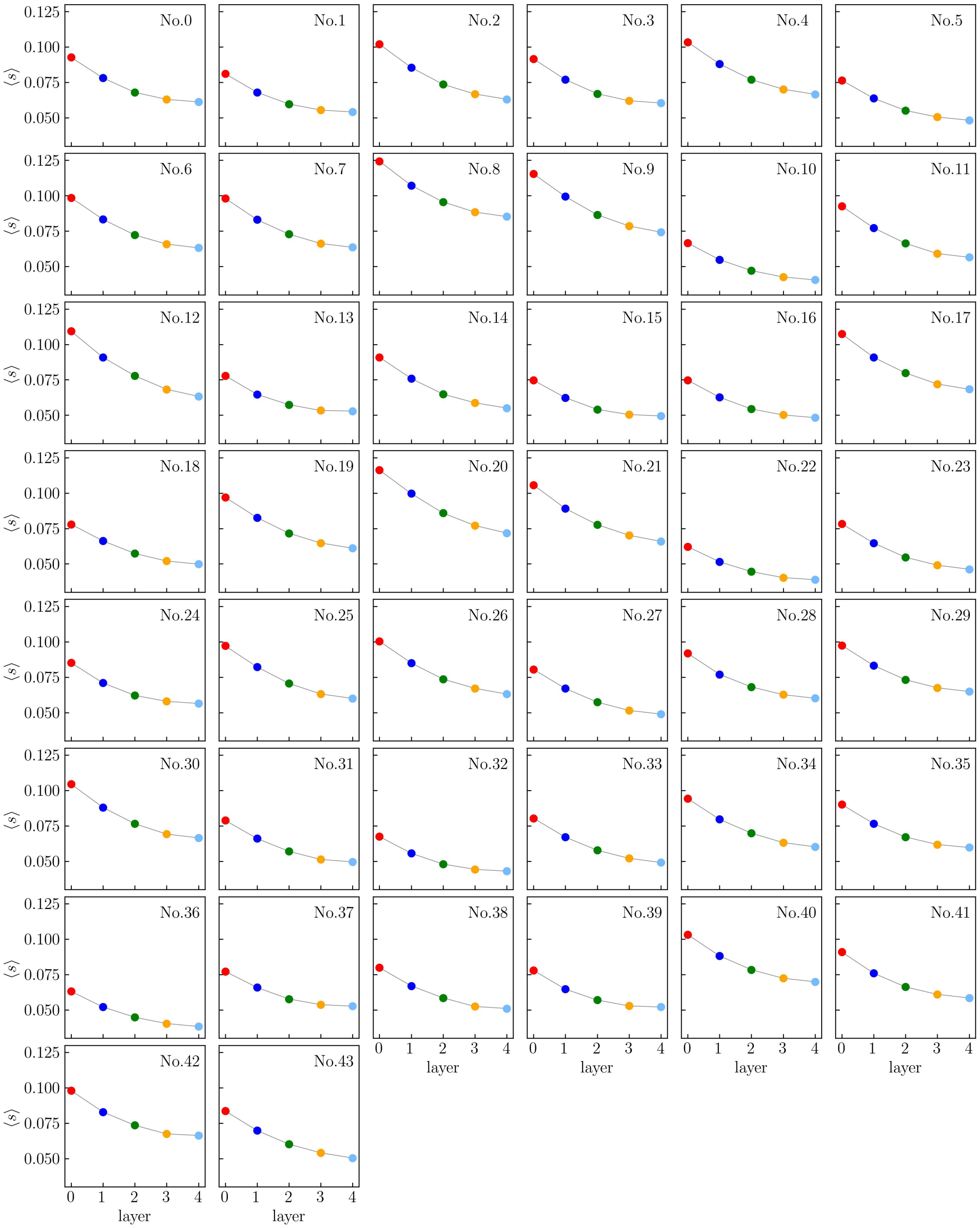

B: Evolution of average strength in consecutive layers.

II. SELF-SIMILARITY

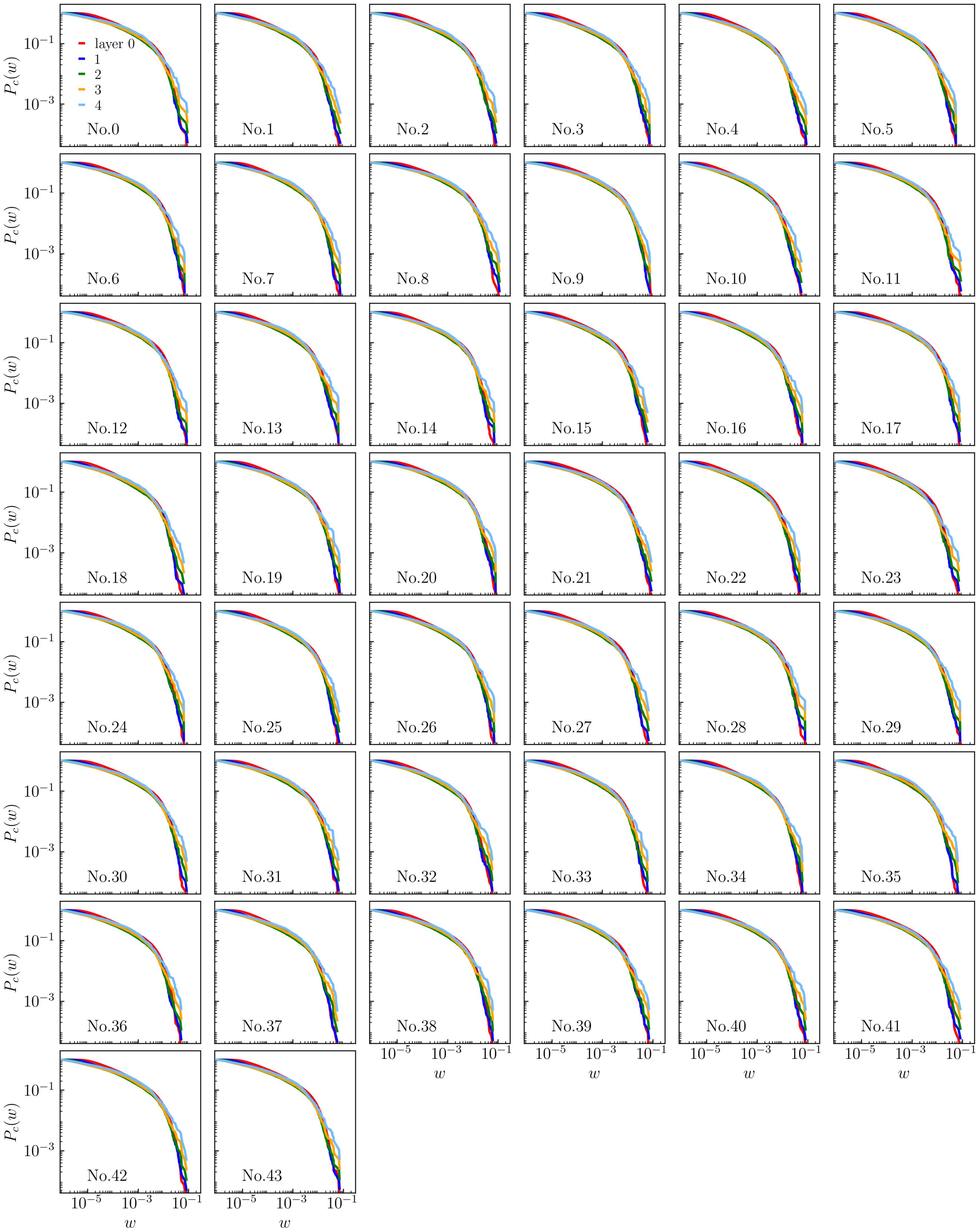

C: Complementary cumulative weight distribution in consecutive layers..

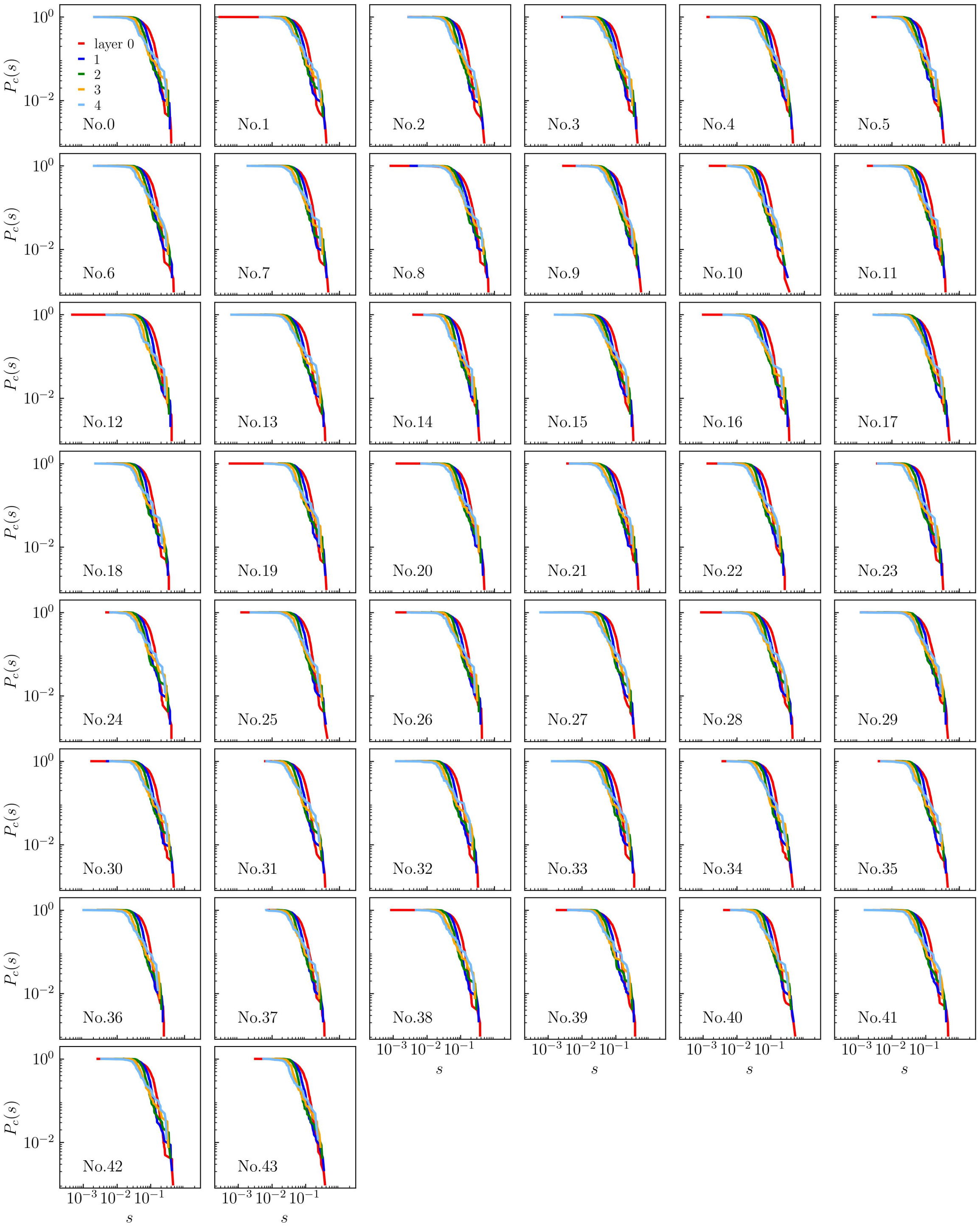

D: Complementary cumulative strength distribution in consecutive layers.

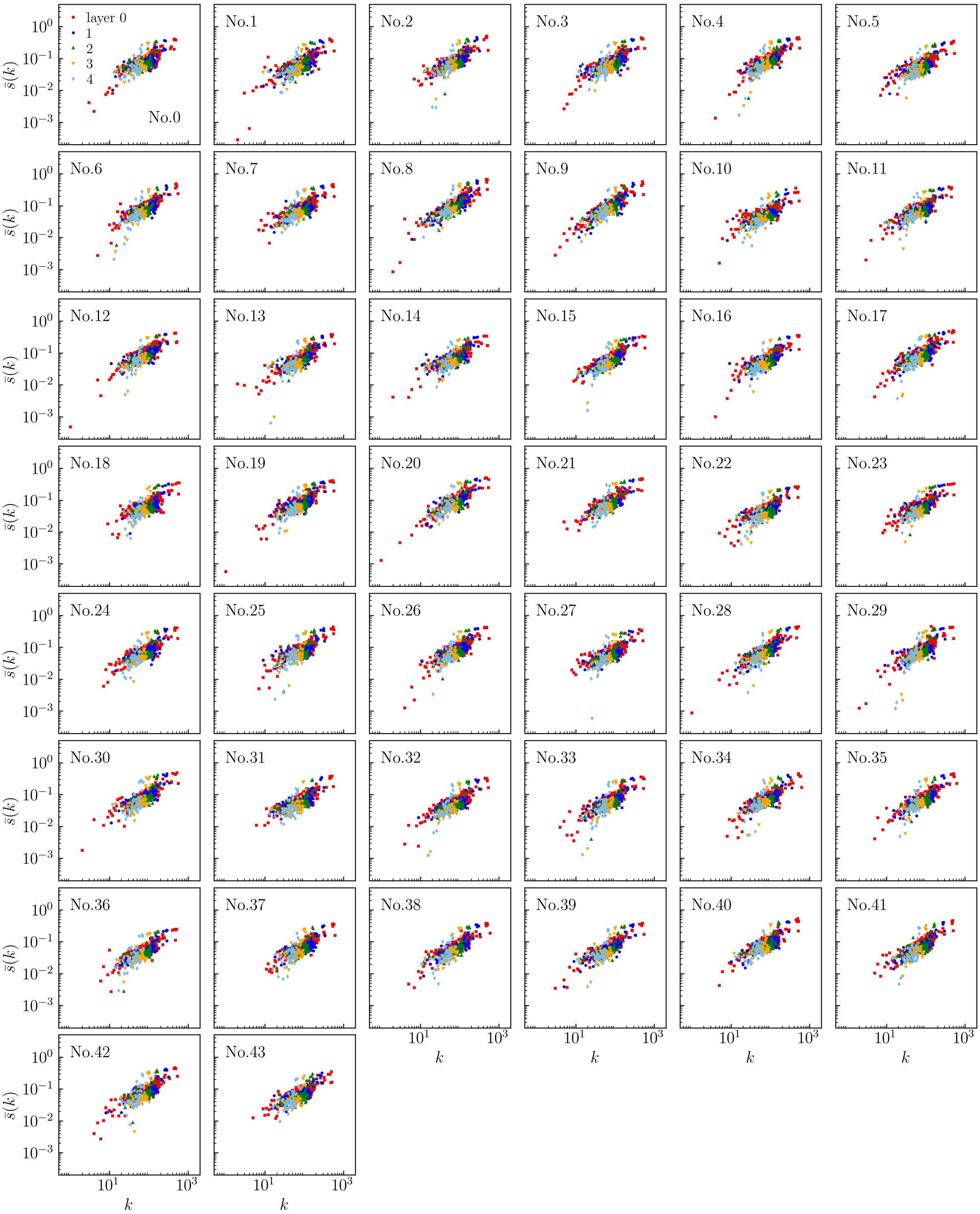

E: Strength-degree correlation in consecutive layers.

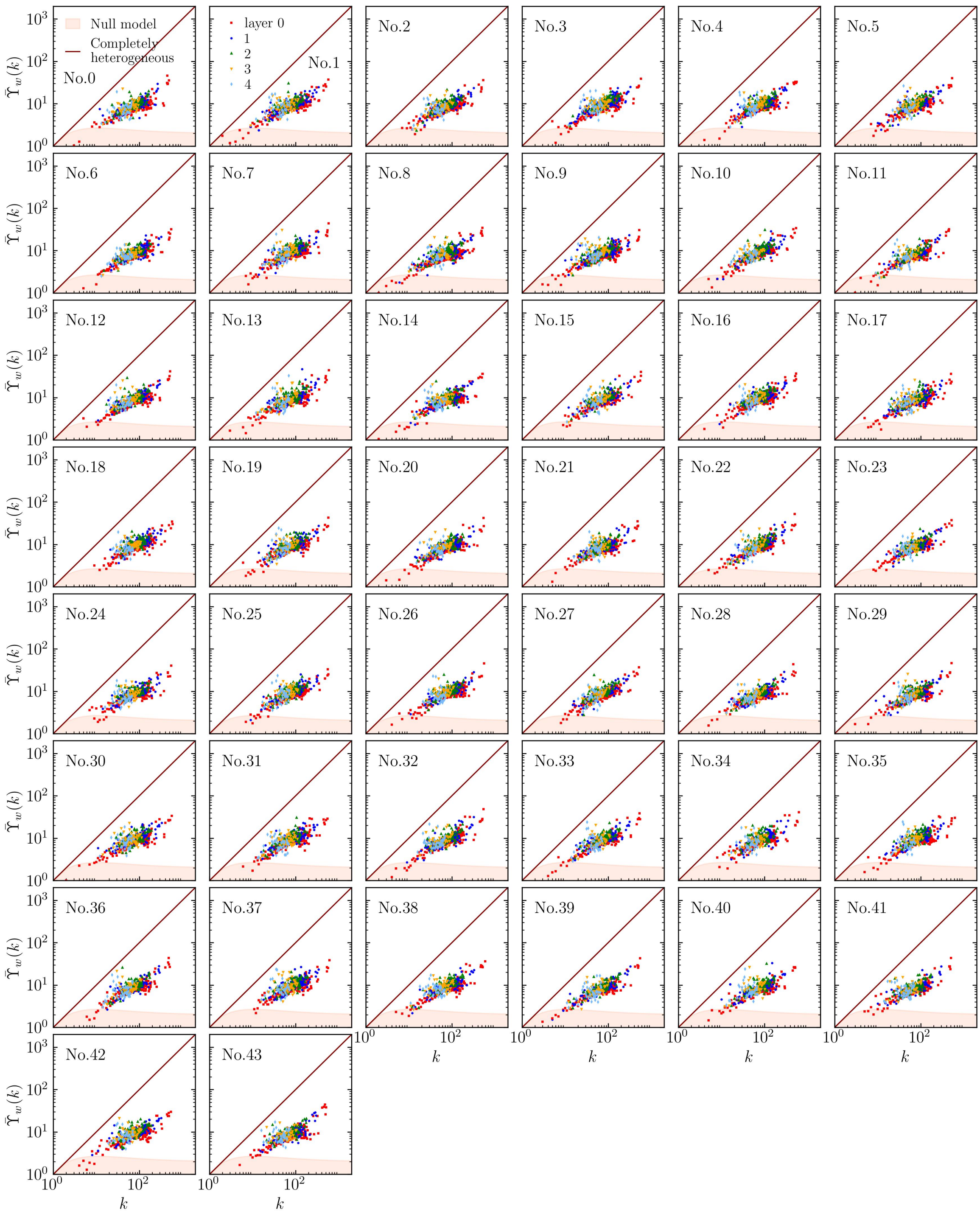

F: Disparity measure in consecutive layers.

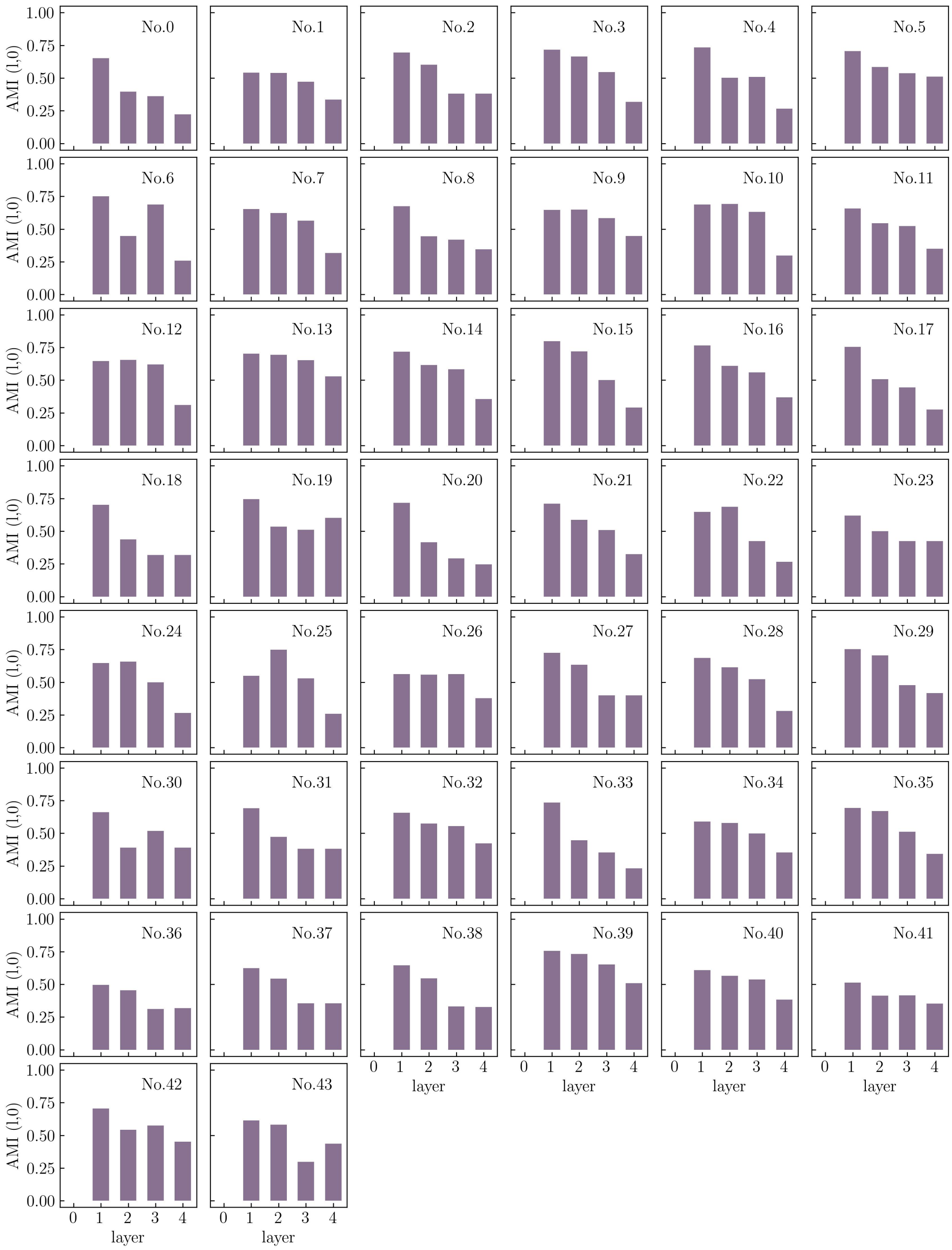

G: Adjusted Mutual Information between communities in layer l (x-axis) and communities in layer 0.

III. WEAK TIES HYPOTHESIS

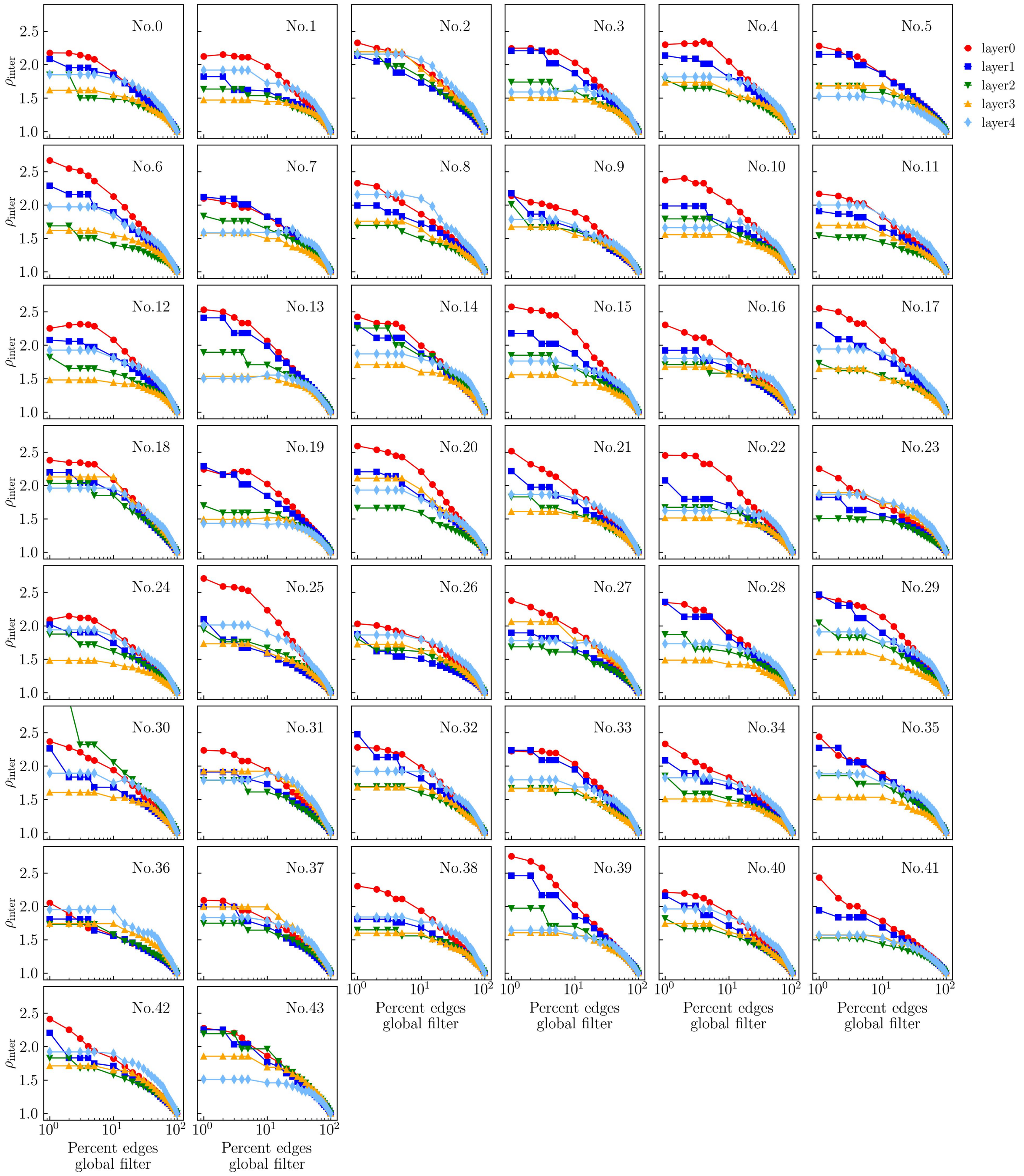

H: Normalized density of intermodular connections versus the percent of edges considered. The x axis X% means we are taking into account the X% of connections with lowest weight. X-axis in log scale.

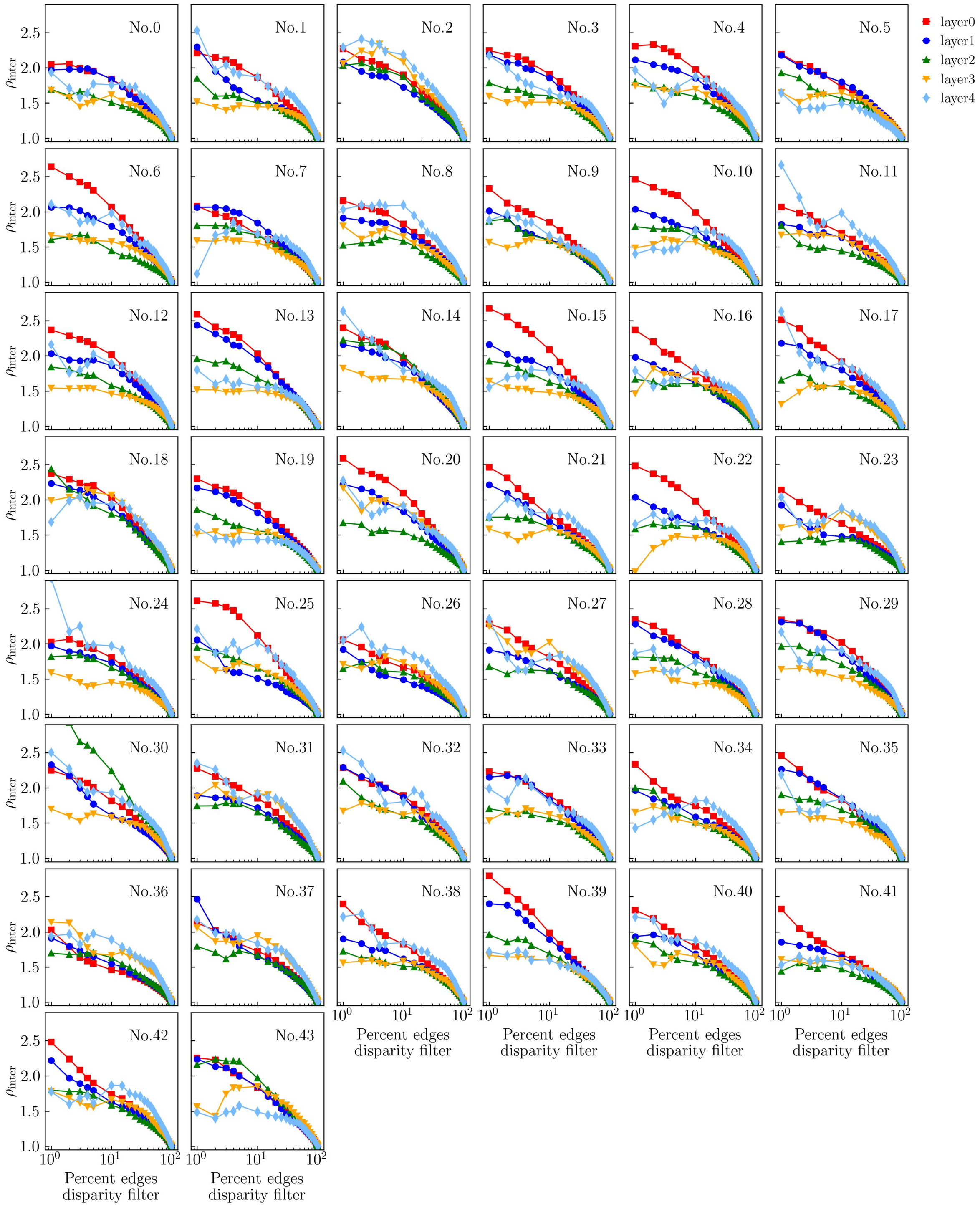

I: Normalized density of intermodular connections versus the percent of edges considered. The x axis X% means we are taking into account the X% of connections with lowest $1-\alpha$. X-axis in logscale.

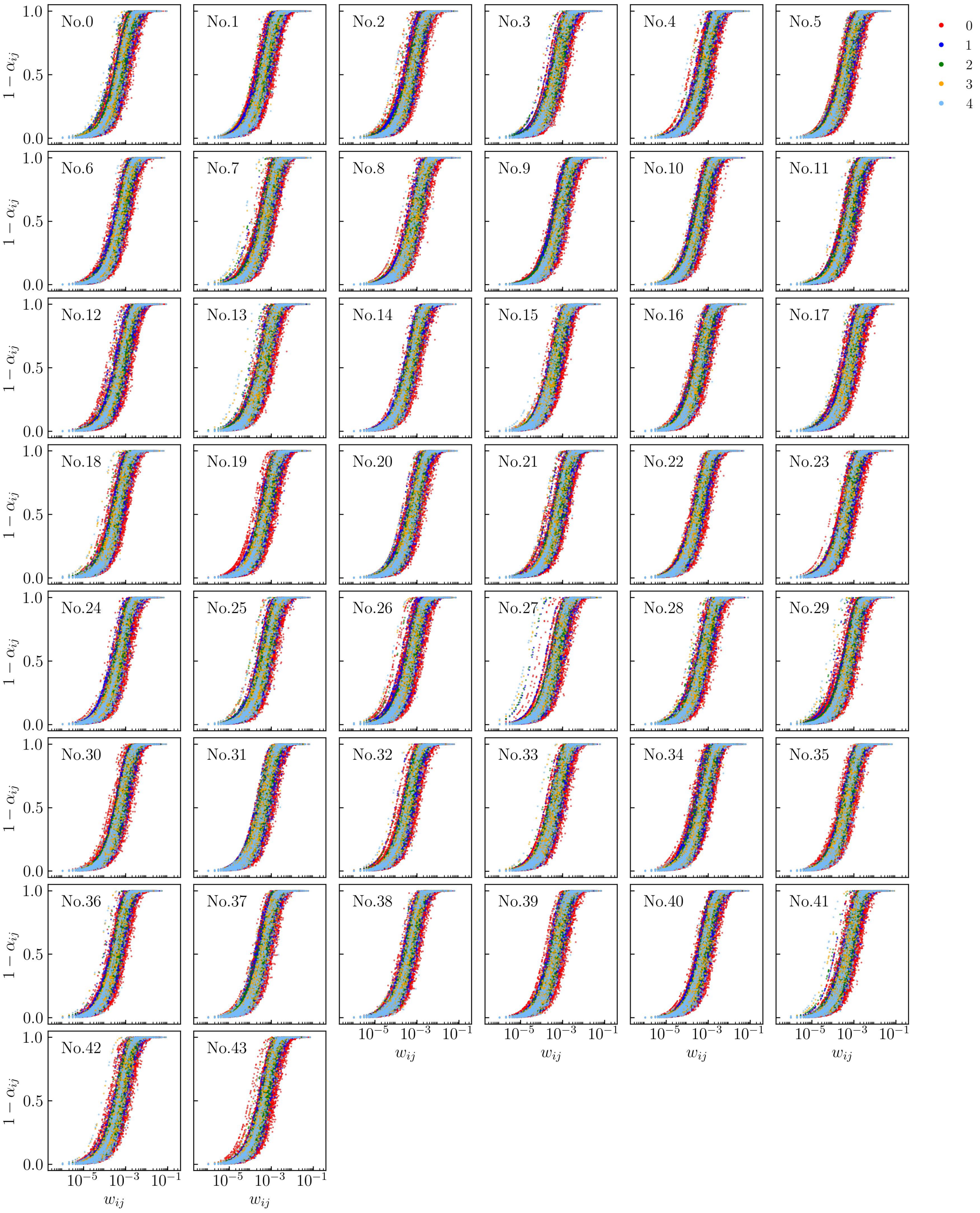

J: Relationship between weight and the confidence of the edge, $1-\alpha$. Legend corresponds to the layer.

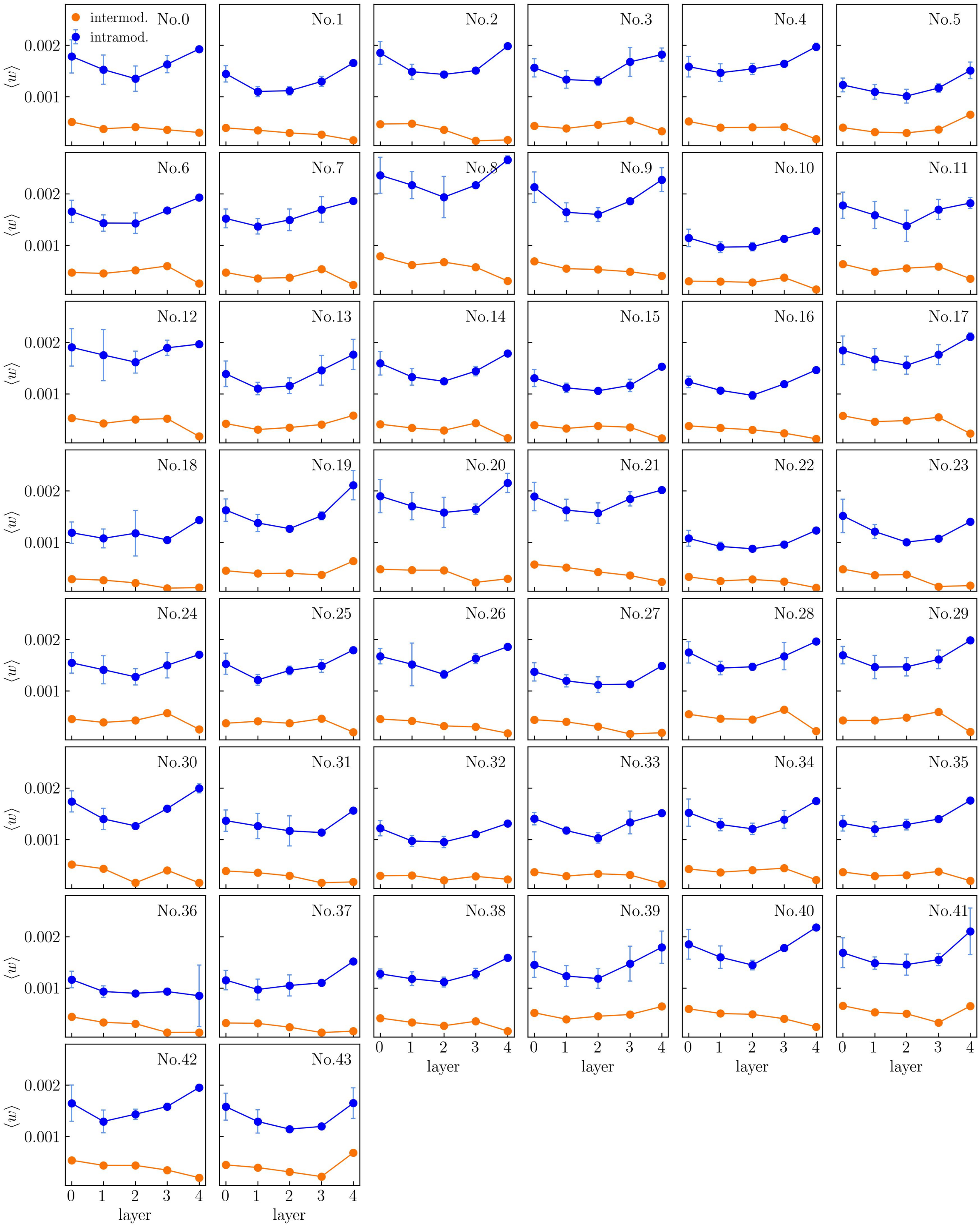

K: Average weight inside modules (intramodular links) compared to average weight outside modules (intermodular links), when modules are computed using the Louvain algorithm. The average weight inside modules is computed as the mean of the average weights in the different modules.

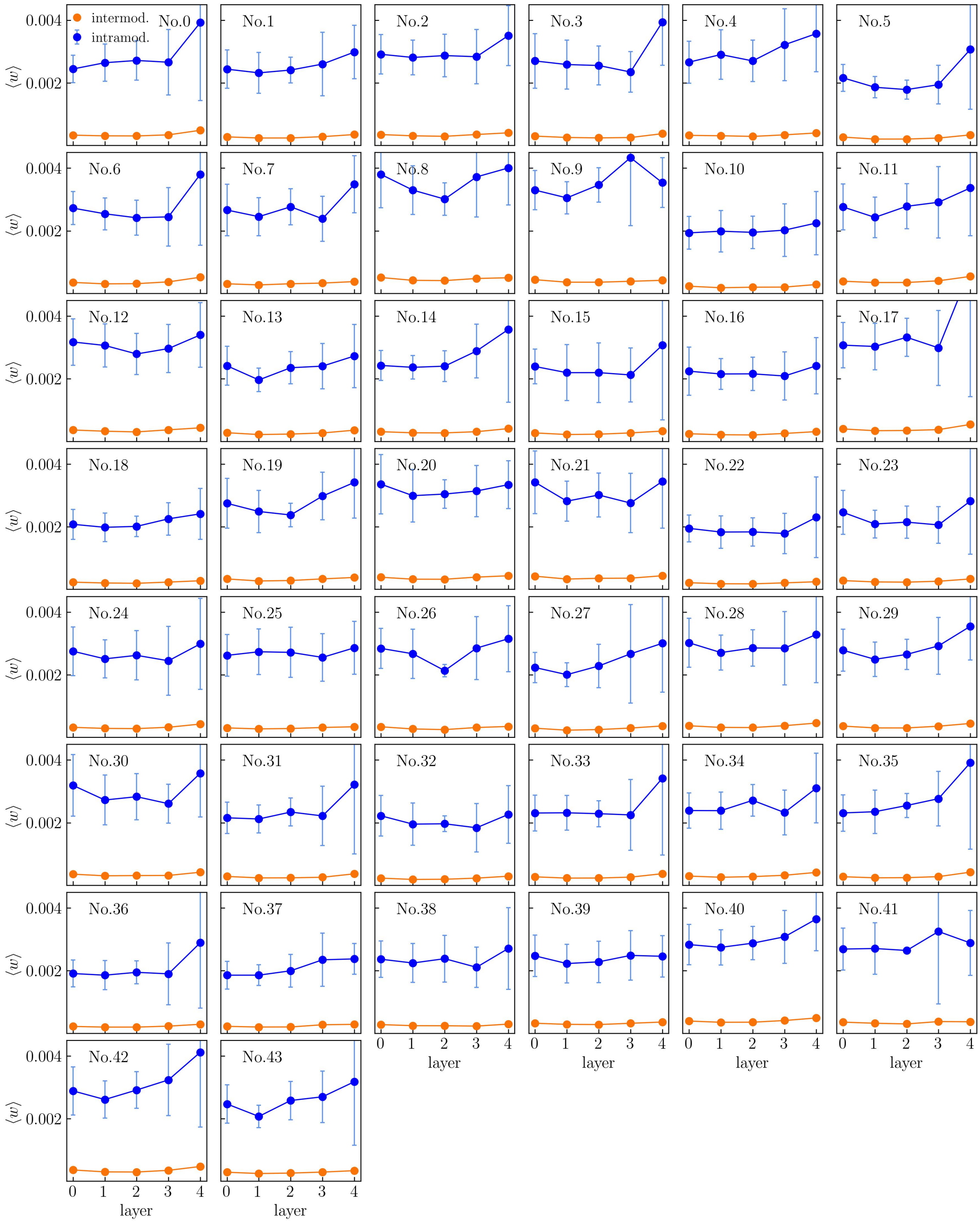

L: Average weight inside modules (intramodular links) compared to average weight outside modules (intermodular links), when modules are computed using the weighted Louvain algorithm. The average weight inside modules is computed as the mean of the average weights in the different modules.

IV. FRANKIE

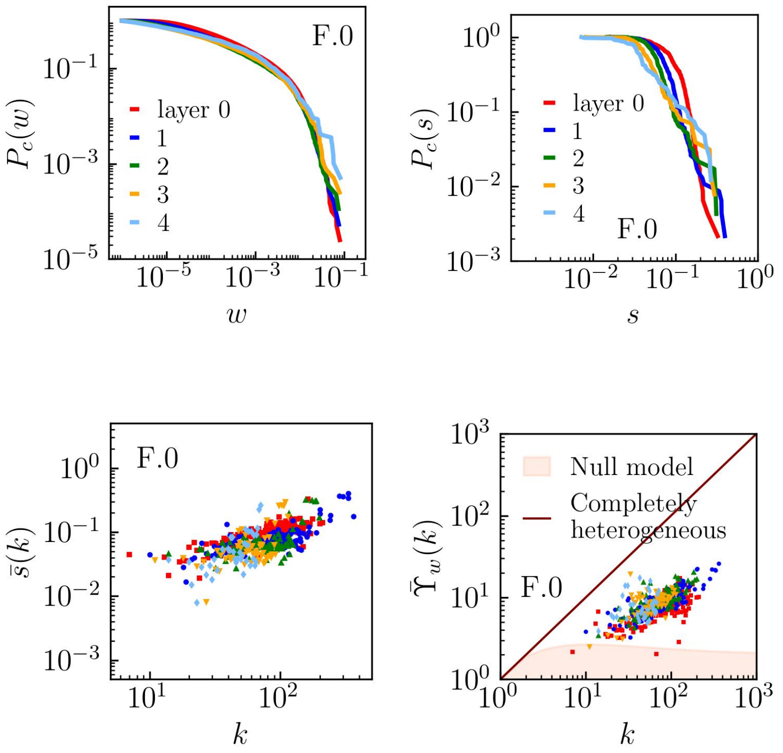

M: Weighted network properties for the group-representative connectome of the dataset, here referred as Frankie.

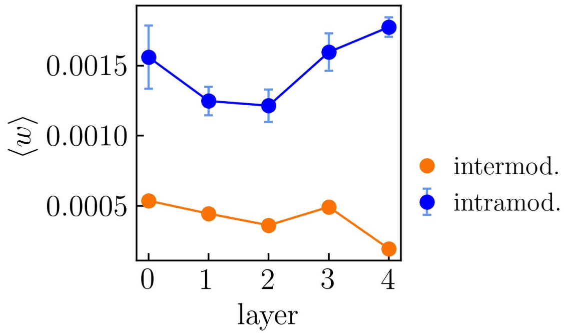

N: Average weight inside modules (intramodular links) compared to average weight outside modules (intermodular links), when modules are computed using the unweighted Louvain algorithm. The average weight inside modules is computed as the mean of the average weights in the different modules.

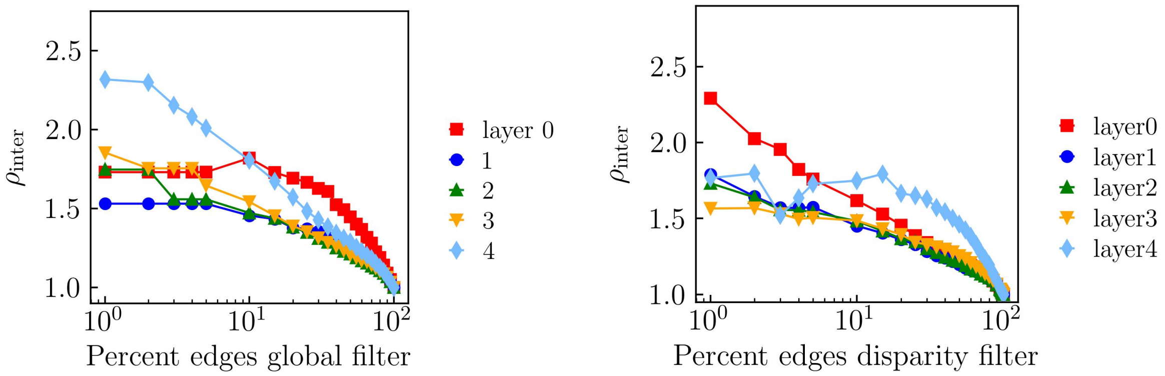

O: A) Normalized density of intermodular connections. The x axis X% means we are taking into account the X% of connections with lowest weight. X-axis in log scale. B) Same as A) but the x axis X% means we are taking into account the X% of connections with lowest $1 - \alpha$.

V. GR MODEL

A. Flow of averages

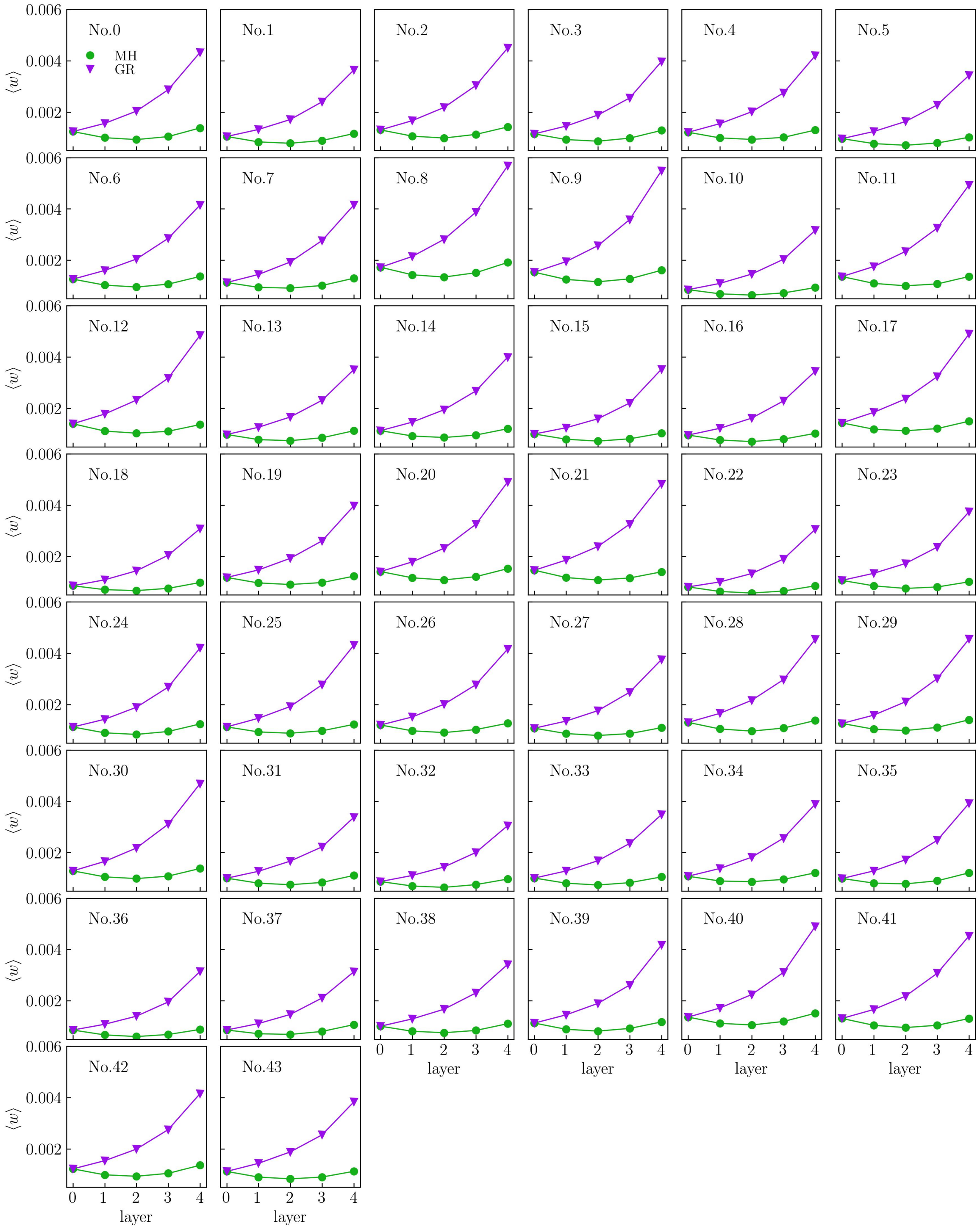

P: Comparison of the evolution of average weight in consecutive layers for the experimental data (in green) and the GR model (in purple).

B. Self-similarity

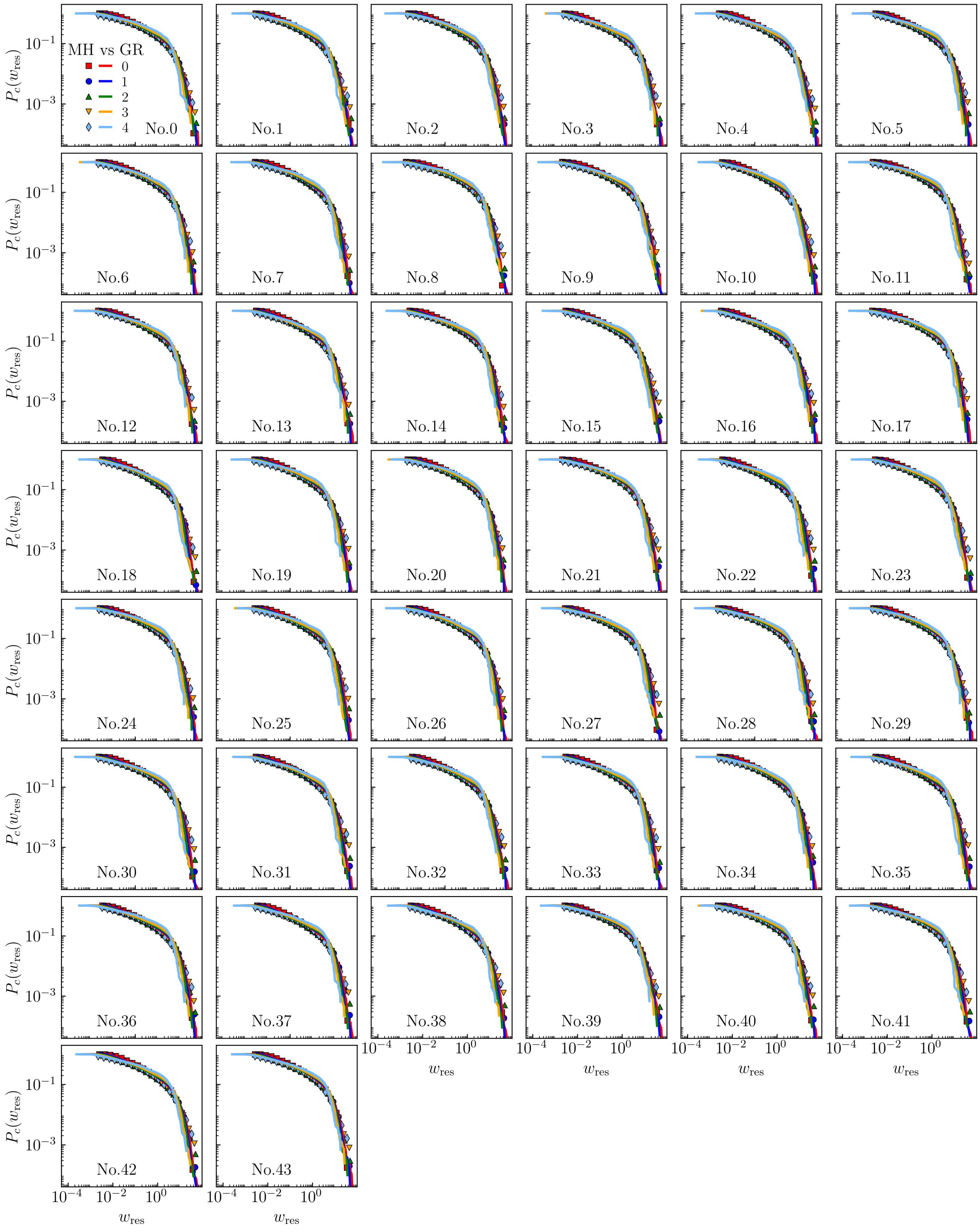

Q: Comparison of the complementary cumulative weight distributions obtained from the experimental data (points) and our model (solid lines) for the five layers considered. Results rescaled by the average weight of each layer.

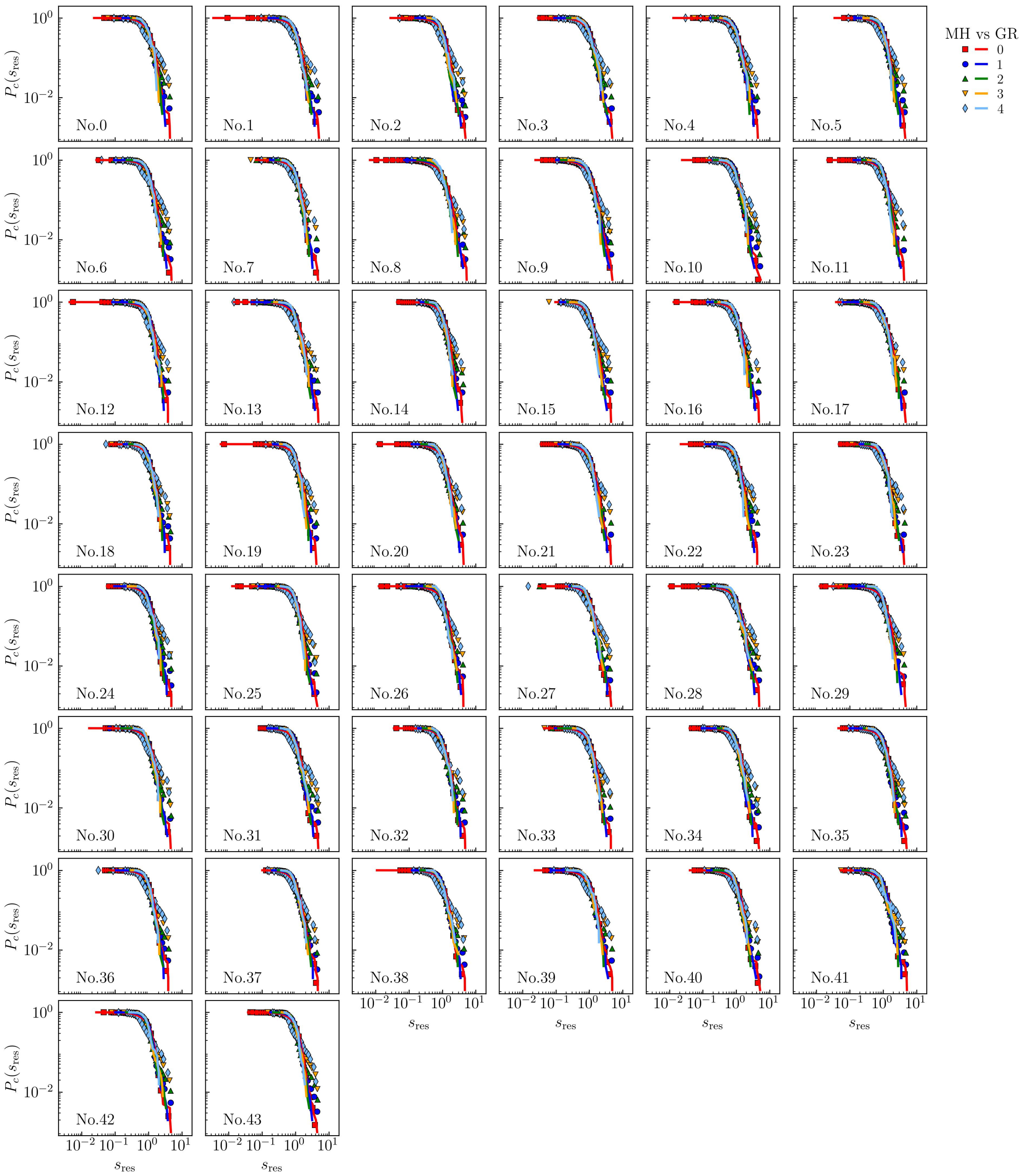

R: Comparison of the complementary cumulative strength distributions obtained from the experimental data (points) and our model (solid lines) for the five layers considered. Results rescaled by the average strength of each layer.

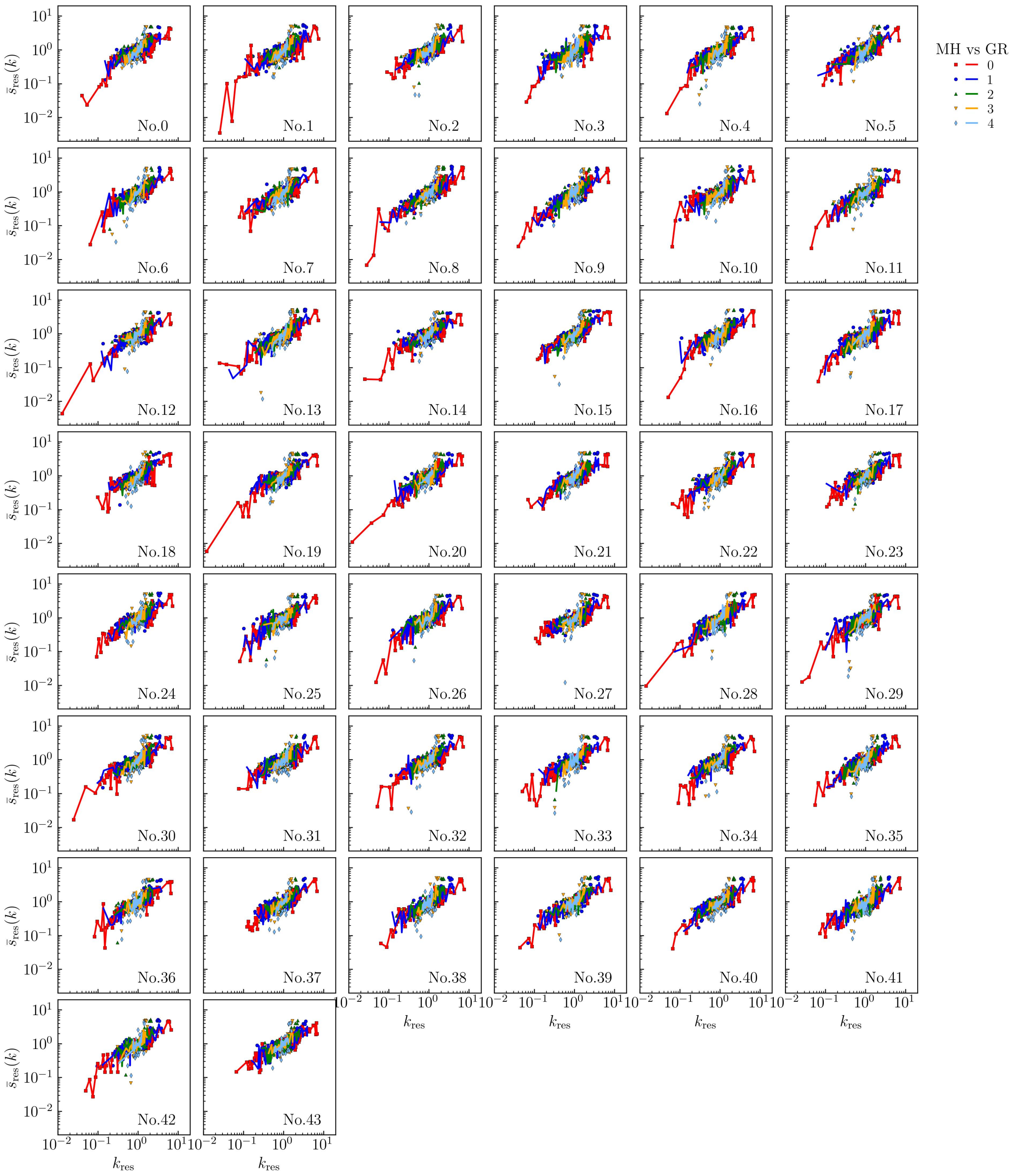

S: Comparison of the strength-degree relationship obtained from the experimental data (points) and our model (solid lines) for the five layers considered. Results rescaled by the average strength and degree of each layer.

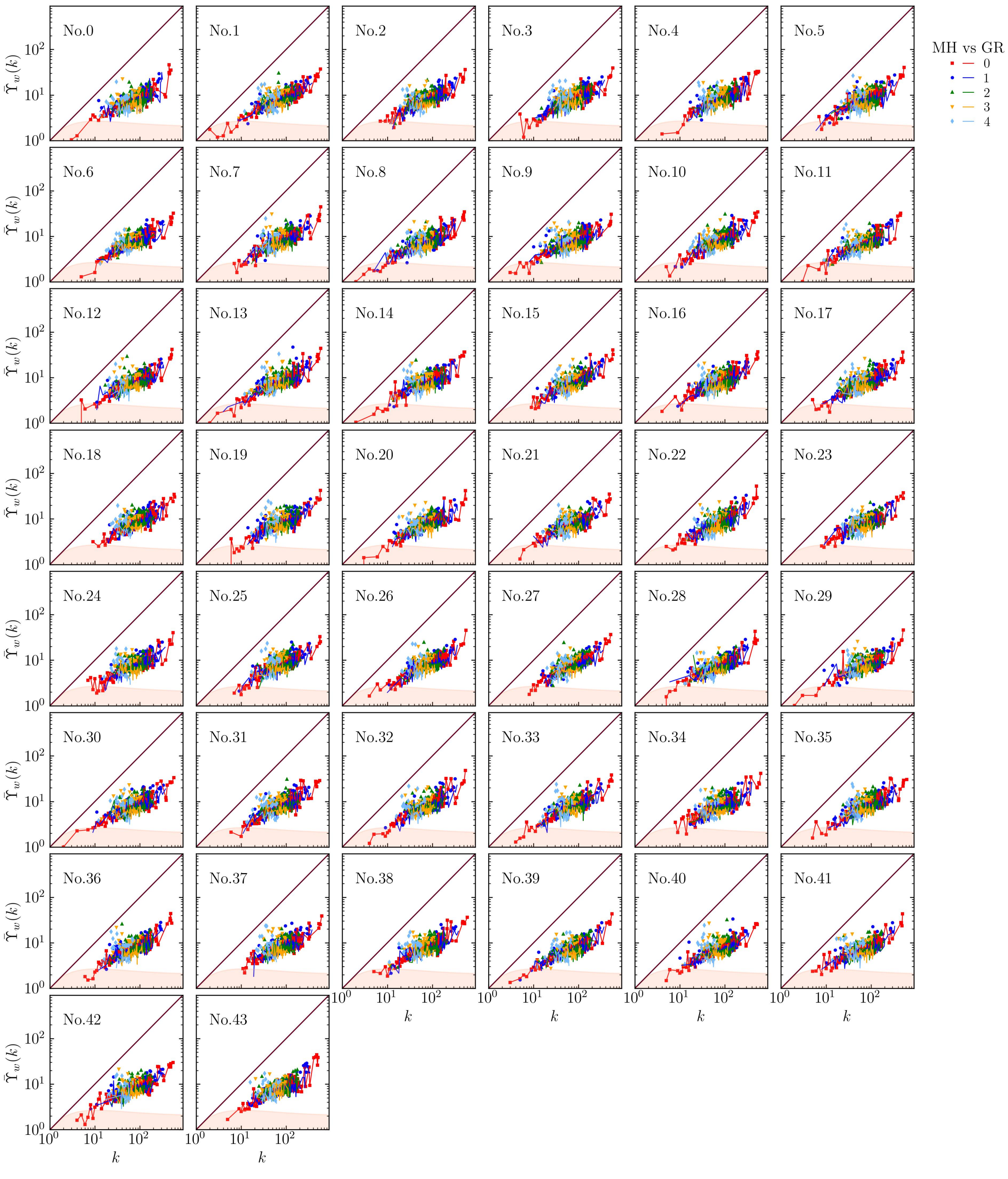

T: Comparison of the disparity measure obtained from the experimental data (points) and our model (solid lines) for the five layers considered.

C. Weak ties in GR model

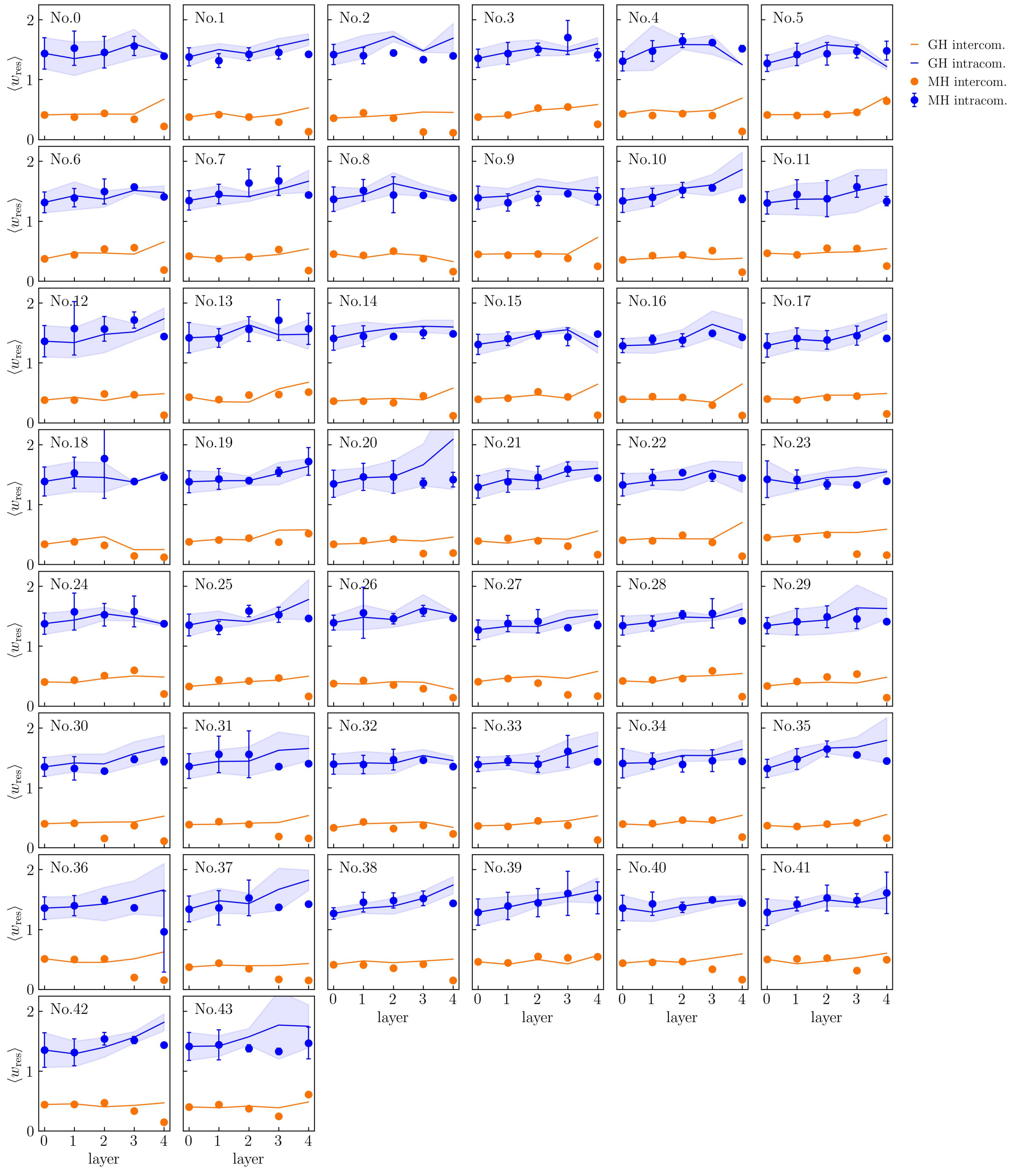

U: Comparison between results of the renormalized networks (lines and dashed area) and the experimental connectomes (dots and errorbars). Average weight inside modules (intramodular links) compared to average weight outside modules (intermodular links), when modules are computed using the Louvain algorithm. The average weight inside modules is computed as the mean of the average weights in the different modules. Results rescaled by the average weight of each layer.

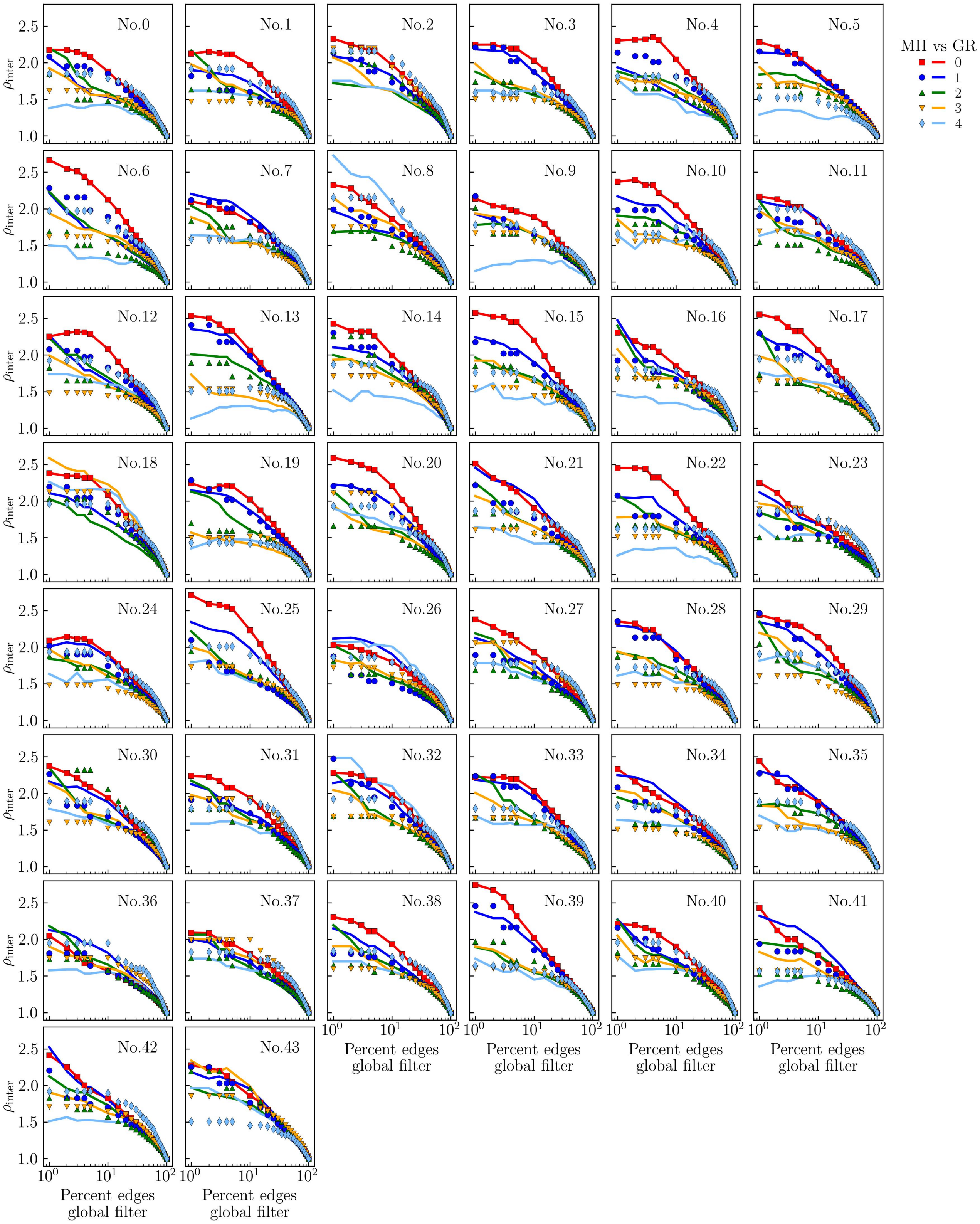

V: Comparison between results of the renormalized layers using the GR model (lines) and the experimental results (points). Normalized density of intermodular connections. The x axis X% means we are taking into account the X% of connections with lowest weight. X-axis in logscale.

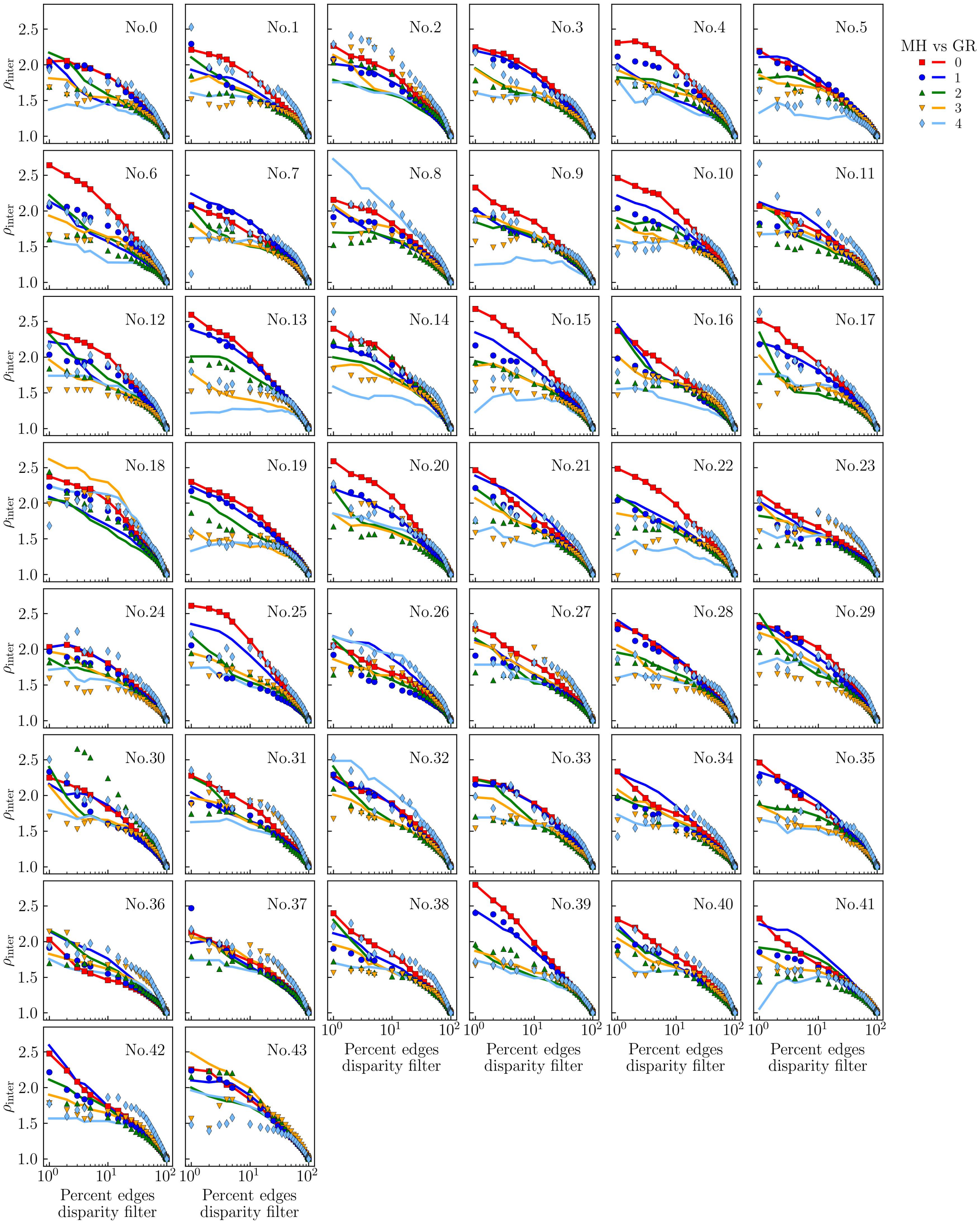

W: Comparison between results of the renormalized layers using the GR model (lines) and the experimental results (points). Normalized density of intermodular connections. The x axis X% means we are taking into account the X% of connections with lowest $1-\alpha$. X-axis in logscale.

VI. NULL MODELS

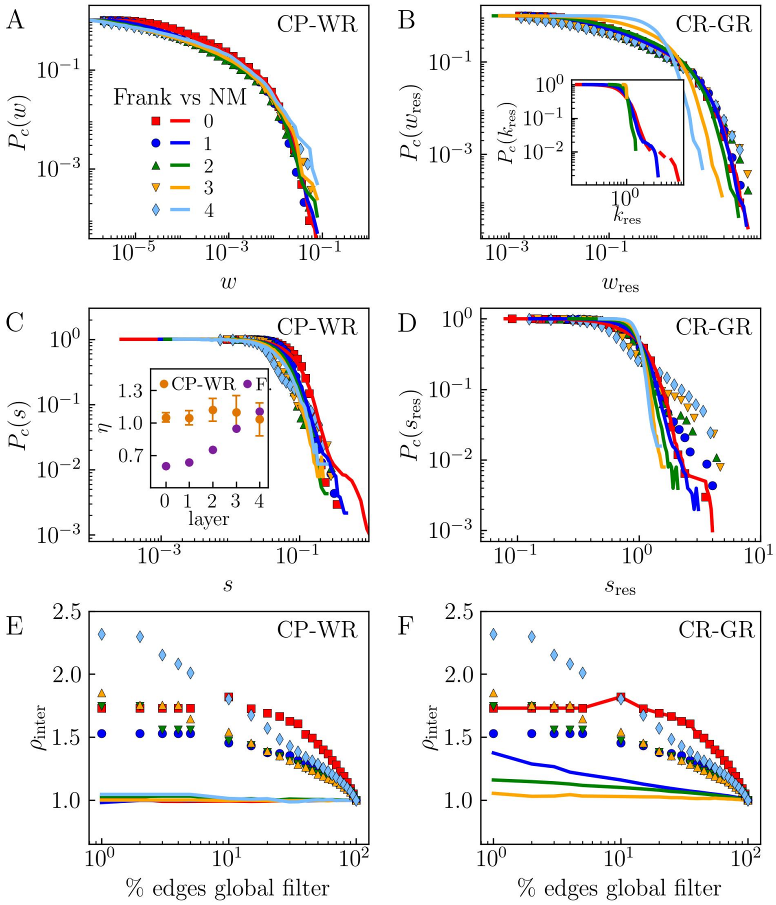

X: Null models for group-representative HCP. In all plots the dots correspond to experimental data and the curves to the null models. A, B) Complementary cumulative weight distributions. Inset in B: complementary cumulative degree distributions. C, D) Complementary cumulative strength distributions. Inset in C: exponent of the $s \sim k^{\eta}$ relationship. The mean and standard deviation of the ensemble is represented by the solid line and the shadowed area. E, F) Normalized density of intermodular links versus percent of edges considered by global thresholding. In the CR-GR, weight and strength have been rescaled by the average weight and strength of the respective layer.
